# Local Delivery of Therapeutics to the Inner Ear: The State of the Science

**DOI:** 10.3389/fncel.2019.00418

**Published:** 2019-10-09

**Authors:** Caroline R. Anderson, Carol Xie, Matthew P. Su, Maria Garcia, Helen Blackshaw, Anne G. M. Schilder

**Affiliations:** ^1^evidENT, Ear Institute, University College London, London, United Kingdom; ^2^NIHR University College London Hospitals Biomedical Research Centre, London, United Kingdom

**Keywords:** drug delivery, inner ear, novel therapeutics, systematic review, intratympanic, intracochlear

## Abstract

**Background:** Advances in the understanding of the genetic and molecular etiologies of inner ear disorders have enabled the identification of therapeutic targets and innovative delivery approaches to the inner ear. As this field grows, the need for knowledge about effective delivery of therapeutics to the inner ear has become a priority. This review maps all clinical and pre-clinical research published in English in the field to date, to guide both researchers and clinicians about local drug delivery methods in the context of novel therapeutics.

**Methods:** A systematic search was conducted using customized strategies in Cochrane, pubmed and EMBASE databases from inception to 30/09/2018. Two researchers undertook study selection and data extraction independently.

**Results:** Our search returned 12,200 articles, of which 837 articles met the inclusion criteria. 679 were original research and 158 were reviews. There has been a steady increase in the numbers of publications related to inner ear therapeutics delivery over the last three decades, with a sharp rise over the last 2 years. The intra-tympanic route accounts for over 70% of published articles. Less than one third of published research directly assesses delivery efficacy, with most papers using clinical efficacy as a surrogate marker.

**Conclusion:** Research into local therapeutic delivery to the inner ear has undergone a recent surge, improving our understanding of how novel therapeutics can be delivered. Direct assessment of delivery efficacy is challenging, especially in humans, and progress in this area is key to understanding how to make decisions about delivery of novel hearing therapeutics.

## Introduction

### Rationale

Recent advances in the understanding of the varied genetic and molecular etiologies of inner ear dysfunction have enabled the identification of multiple potential therapeutic targets (Müller and Barr-Gillespie, 2015; Mittal et al., 2017). This has accelerated the development of a range of novel therapeutics, from small molecule drugs to gene and cell therapies, several of which are starting to enter the clinical trial domain (Schilder et al., 2018)^1^,^2^. Whilst significant progress has been achieved with regards to therapeutic identification and development, the most effective mechanisms of therapeutic delivery to the inner ear have yet to be determined. The importance of delivery cannot be understated; the success of any novel therapeutic depends on selection of the most suitable method for the pharmacokinetic profile of the individual agent, and the balance of risks associated with delivery against the potential benefit of the treatment (Salt and Plontke, 2009; Plontke and Salt, 2018).

The inner ear poses a unique pharmacokinetic and pharmacodynamic challenge due to its anatomical location, epithelial barriers and the relatively unstirred nature of the perilymph (Salt, 2002). Local delivery is an attractive option as it overcomes concerns regarding toxicity or side effects associated with systemic administration (Plontke et al., 2014; Salt and Plontke, 2018), allowing higher concentrations to reach the inner ear (Liu et al., 2018). It can be broadly divided into intra-tympanic or intra-cochlear routes, each with many options for delivery method and therapeutic formulation (Liu et al., 2018; Peppi et al., 2018; Salt and Plontke, 2018). Middle ear approaches, such as transtympanic injection, rely on simple diffusion through the epithelial barriers of the round and/or oval window (Salt and Plontke, 2018). This can lead to the formation of concentration gradients, with variable concentrations reaching more apical regions of cochlea, and potentially insufficient levels of the therapeutic reaching the basal regions (Salt et al., 2007; Liu et al., 2014; Li et al., 2018; Salt and Plontke, 2018). Delivery using sustained release formulations, magnetically targeted delivery, and nanoparticles (Pyykkö and Jing Zou, 2013; Shapiro et al., 2014; Pyykkö et al., 2016) aims to overcome these problems, but they remain a significant concern. Intra-cochlear therapeutic delivery offers the best control of delivery at the cost of the highest risk to hearing, although the problem of base-apex gradient formation remains (Hahn et al., 2012; Salt et al., 2017). Cochlear implant (CI) associated delivery presents a unique opportunity to develop this route for a subset of patients (Hochmair et al., 2006; Budenz et al., 2012; Roemer et al., 2016; Plontke et al., 2017).

At present there is a limited understanding of how delivery method, therapeutic agent and formulation, and disease process interact. This makes choosing a delivery method for a given therapeutic a major challenge faced by all involved in the development, production and administration of novel therapeutics, including discovery scientists, clinicians, industry, regulators, and patients. There is little to guide these stakeholders as to the best delivery method for a given therapeutic in a given patient group. A systematic review of the available literature is therefore necessary in order to collate and make accessible the currently available information.

### Aims

The aims of this paper are to map all research undertaken so far in the field of local delivery of therapeutics to the inner ear, and to signpost to information to support decisions about delivery methods in the context of novel therapeutics.

### Objectives

- To identify all relevant literature on local delivery of therapeutics to the inner ear.
- To understand the ways in which delivery methods are tested, and delivery efficacy assessed.
- To understand how the field of local therapeutic delivery to the inner ear has evolved, by exploring trends in publication type, experimental design, therapeutic classes and delivery methods.
- To identify which local delivery routes and methods have been tested in the pre-clinical and clinical research settings.
- To create searchable tables of the current literature on local delivery of therapeutics to the inner ear to signpost interested parties to the work that is most relevant to them.

## Methods

### Study Design

A systematic review of the published scientific literature.

### Systematic Review Protocol

The review protocol was registered with PROSPERO and is available from http://www.crd.york.ac.uk/PROSPERO/display_record.php?ID=CRD42018105903.

### Search Strategy

The search strategy was designed using key words for inner ear therapeutic delivery and customized for each database (available in Supplementary Material Section 1) with assistance provided by the University College London Ear Institute & Action on Hearing Loss Libraries. No restriction was placed on study design. The search was restricted to English language articles.

### Data Sources, Studies Sections, and Data Extraction

Pubmed, Embase and the Cochrane library were searched from inception to May 30th, 2017. The search was updated on September 30th 2018 and the results combined with the initial search.

### Study Selection

Titles and abstracts were screened for inclusion independently by two review authors, with discrepancies resolved by full text review and discussion within the study team.

All studies and reviews of local delivery of therapeutics to the inner ear in human or animal models, where the architectural structure of the inner ear was maintained, were included. Studies that included only systemic therapeutic administration, data on *in vitro* experiments, animal explants, mechanical models and models utilizing computer-generated data were excluded. Inclusion and exclusion criteria are summarized in Table 1.

**Table 1:** Inclusion and exclusion criteria.

**Inclusion criteria**	**Exclusion criteria**
Any study design	Studies reporting only intra-uterine delivery to otocyst
Local delivery of therapeutic to inner ear target	Studies reporting only *in vitro* work
Intact inner ear architecture and anatomy	Studies reporting only explant culture delivery
	Mechanical models
	Computer generated data
	Letters to the editor/Editorials/Expert opinion without references
	Articles not in English

### Data Extraction

Two authors carried out full text review and data extraction independently, with discrepancies resolved by discussion within the study team. During full text review, papers were identified as original research or review articles. Data extracted from review articles was limited to the type of review; narrative, systematic, or systematic with meta-analysis. Data extracted from original research articles was year of publication, experimental model (animal or human, living or temporal bone), method of assessment of efficacy of delivery, delivery method(s), therapeutic agent, therapeutic formulation, and underlying disorder targeted or treated.

### Data Categorization

Extracted data was grouped as shown in Table 2.

**Table 2:**
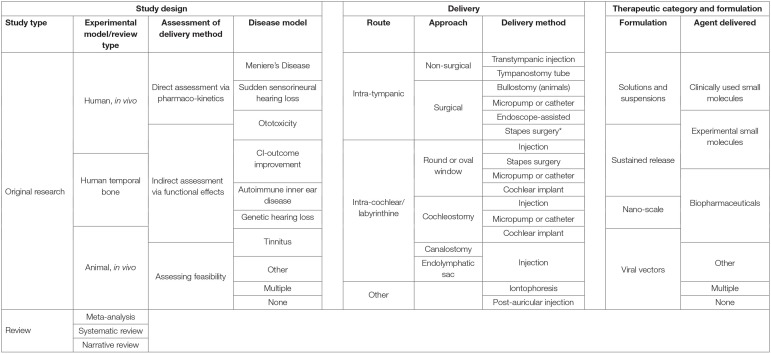
Categorization of extracted data in local therapeutic delivery to the inner ear.

*Stapedectomy, followed by placing therapy on oval window.

Categorization of the method of assessment of delivery efficacy was designed to aid understanding of how research is designed. Studies directly investigating the efficacy of delivery (e.g., by concentration in perilymph, or presence or absence of a delivered substance in the target tissue) were classified as “direct assessment of delivery efficacy via pharmacokinetics.” Studies investigating the functional, toxic or clinical effects of a therapeutic agent delivered to the inner ear were classified as “indirect assessment of delivery efficacy via functional effects” (as an indirect marker of delivery method efficacy). Studies focusing on the practicalities and/or adverse effects of a proposed delivery method or therapeutic formulation, without measuring the efficacy of delivery, were classified as “assessing feasibility.”

**Figure 1:**
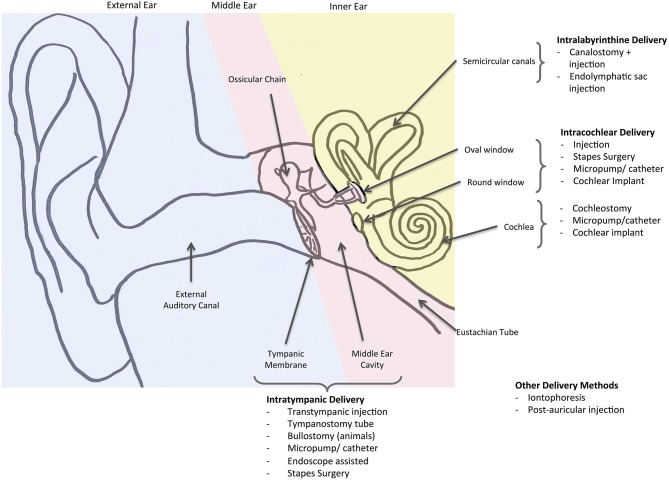
Schematic figure summarizing the structure of the inner ear and delivery routes.

Delivery methods were categorized by route, approach, and method, as shown in Table 2, and in schematic form in Figure 1.

Formulation was defined as the combination of therapeutic and any other substance that affects the pharmacokinetic profile of that therapeutic (Kulkarni and Shaw, 2016). They were categorized as solutions and suspensions, sustained release formulations, nano-scale formulations and viral vectors (Fukui and Raphael, 2013; Kulkarni and Shaw, 2016; Pyykkö et al., 2016; Kim, 2017; Liu et al., 2018). These categories are defined with examples in Table 3. Therapeutics were categorized as clinically used small molecules, experimental small molecules, biopharmaceuticals, other, multiple, and none, as defined in Table 4 (Nakagawa and Ito, 2005; Budenz et al., 2012; Fukui and Raphael, 2013; Liu, 2014)^3^.

**Table 3:** Categorization of formulations used in therapeutic delivery to the inner ear with definitions and examples.

**Formulation category**	**Definition**	**Examples**
Solutions and suspensions	Soluble therapeutic agent dissolved in solvent or a less soluble agent suspended as particles in the solvent (Kulkarni and Shaw, 2016)	Dexamethasone suspension in 0.9% saline; dexamethasone-phosphate solution in saline
Sustained release formulations	Combination of therapeutic with any substance designed to prolong the exposure of the therapeutic agent to the inner ear (Liu et al., 2013)	Hydrogels, polymers, poloxamers, gelfoam microwick®
Nano-scale formulations	Particles 1–100 nm in size in at least one dimension (Pyykkö et al., 2016). Includes nano-scale formulations placed in a sustained release formulation	Nanoparticles, magnetic nanoparticles, liposomes, polymersomes
Viral vectors	Viruses used to deliver normal genes into cells, in place of missing or faulty genes (Fukui and Raphael, 2013)	Adeno-associated virus (AAV), adenovirus

**Table 4:** Categorization of therapeutics locally delivered to the inner ear with definitions.

**Therapeutic category**	**Subcategory**	**Class**	**Definition**
Clinically used small molecules		Corticosteroids Aminoglycosides	Low molecular weight drugs (900 daltons), produced by chemical synthesis and delivered to the inner ear in clinical practice^3^
Experimental small molecules	Therapeutic agents	Local anesthetics Bisphosphonates	Low molecular weight drugs (900 daltons), produced by chemical synthesis and ***not*** delivered to the inner ear in clinical practice^3^
		Antioxidants	
		Antivirals	
		Apoptosis inhibitors	
		NMDA receptor antagonists	
	Toxic agents		Low molecular weight drugs (900 daltons), produced by chemical synthesis^3^. Designed to impair inner ear function thus allowing assessment of delivery
	Contrast media, dyes and fluorescently tagged molecules		Low molecular weight drugs (900 daltons), produced by chemical synthesis^3^. Used for assessment of delivery rather than for therapeutic benefit
Biopharmaceuticals	Protein based therapies	Neurotrophins	Proteins with a biological origin that induce the survival, development and function of neurons^3^
		Monoclonal antibodies	Laboratory produced antibodies, with a biological origin, designed to recognize and bind specific receptors (Liu, 2014)
	Gene correction therapies		Therapies with a biological origin that deliver normal genes into cells, in place of missing or faulty genes (Fukui and Raphael, 2013)
	Cell therapies		Cell transplantation to the inner ear for either regeneration or drug delivery (Nakagawa and Ito, 2005)
Other			
Multiple			Studies using combinations of above classes
None			Empty vehicle delivery, or feasibility studies

This categorization was designed to allow combinations of delivery methods, therapeutic agents and formulations to be considered together. For example, a study evaluating transtympanic injection of a liquid form drug and a study evaluating transtympanic injection of a gel containing nanoparticles containing the same drug would both be classed broadly as intra-tympanic injections, but would be differentiated in the formulation category to allow for the fact that this may alter delivery efficacy.

### Data Analysis

Data was managed in Microsoft Excel and analyzed in R. The annual number of publications in the field was counted as a whole and for each subcategory. Total numbers of publications across each subcategory were counted and displayed as both a total number and a list of references for those publications.

## Results

### Search Results

**Figure 2:**
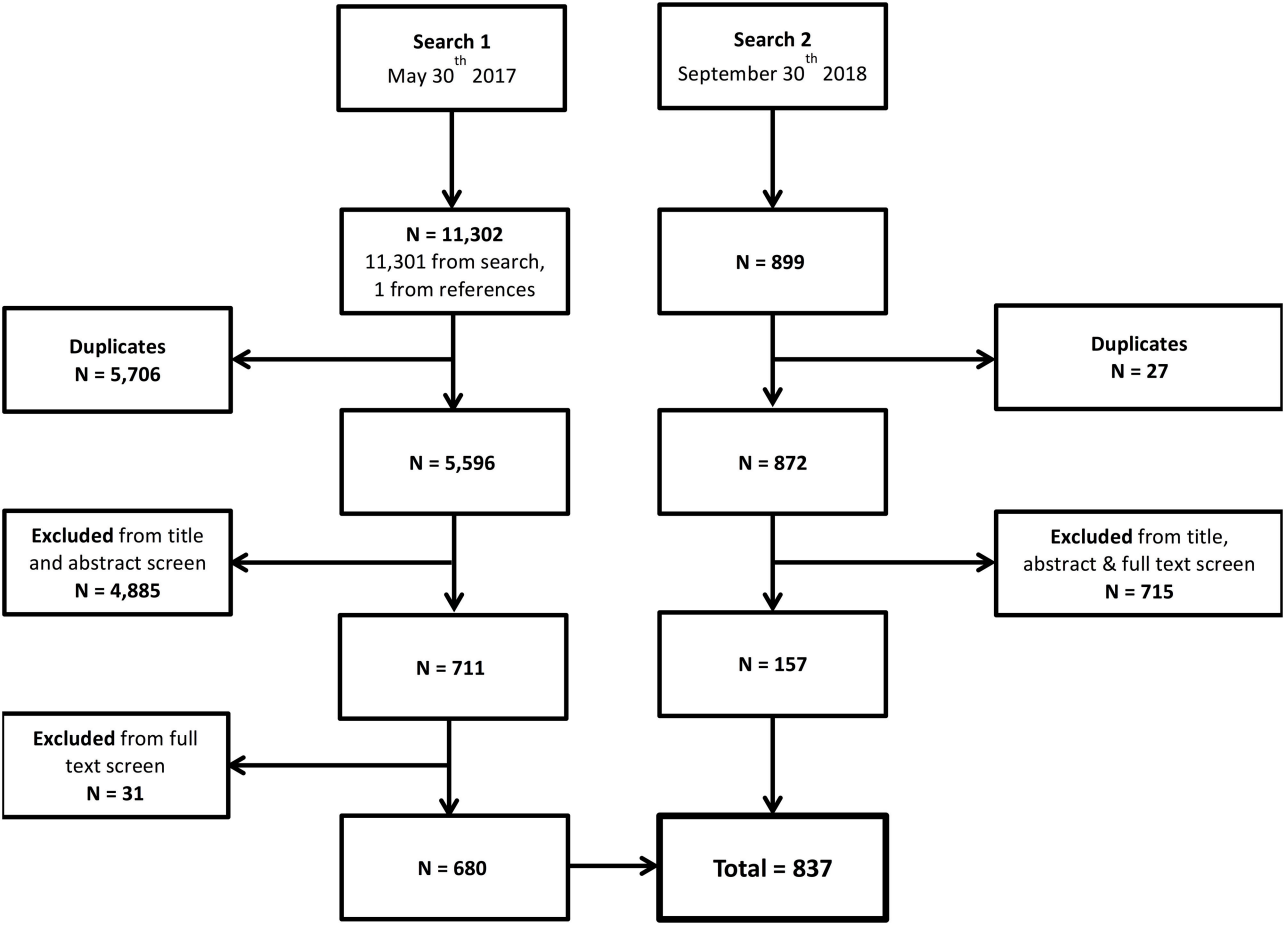
Flow diagram of search results.

Combining the searches from May 30th, 2017 and September 30th 2018 yielded a total of 12,201 records. Removing duplicates left 6,468 unique entries. Title and abstract screening excluded 5,600 articles, with a further 31 excluded during full text screening, leaving a total of 837 papers included in this review (Figure 2).

### Numbers of Publications

Of the 837 papers, 679 were original research papers and 158 were reviews. Review papers were primarily narrative reviews (131/158, 82.9%). Systematic reviews accounted for 18 papers, and meta-analysis for 9 papers (Table 5). Of the original research papers, 315 (46.4%) studied humans and 364 (53.6%) studied animals.

**Table 5:** Study design and assessment of delivery method.

**Study type**	**Assessment of delivery method**	**Human**	**Animal**	**Both**	**Total**
Original research	Direct assessment	11	177	0	188
	Indirect assessment	296	158	0	454
	Feasibility	8	29	0	37
	Total	315	364	0	679
Meta-analysis		9	0	0	9
Systematic review		18	0	0	18
Narrative review		35	29	68	132

Eight studies (1.2%) directly assessed delivery efficacy via pharmacokinetics to the live human inner ear, with a further three directly assessing delivery efficacy in human temporal bones. Animal experimental models were used in 177 (26.0%) papers to directly assess delivery efficacy via pharmacokinetics. The majority of papers used functional effects as an indirect marker of delivery efficacy; 296 (43.6%) studies in humans and 158 (23.2%) in animals. Feasibility of delivery methods, without assessment of delivery efficacy, was assessed in 8 (1.2%) human studies and 29 (4.3%) animal studies.

The most common disease studied was Meniere's disease (or endolymphatic hydrops) with 164 (24.2%) original research papers looking at local therapeutic delivery in this context. Next was idiopathic sudden sensorineural hearing loss (ISSNHL) in 98 (14.4%) studies, followed by ototoxicity with 87 (12.8%) studies.

Four hundred and eighty two (70.9%) original studies investigated intra-tympanic delivery methods, 175 (25.7%) intra-cochlear delivery methods, 19 (2.8%) a combination of the two, and 3 (0.4%) other delivery methods.

Solution and suspension formulations were used in 472 (69.5%) papers, sustained release formulations for 99 (14.6%), nanoscale in 41 (6.0%), and viral vectors in 50 (7.4%). Seventeen papers studied other or multiple formulations.

Clinically used small molecules were studied in 340 (50.1%) publications, experimental small molecules in 106 (15.6%), and biopharmaceuticals in 143 (21.1%). Fifty papers studied combinations or empty formulations.

### Trends in Publications

**Figure 3:**
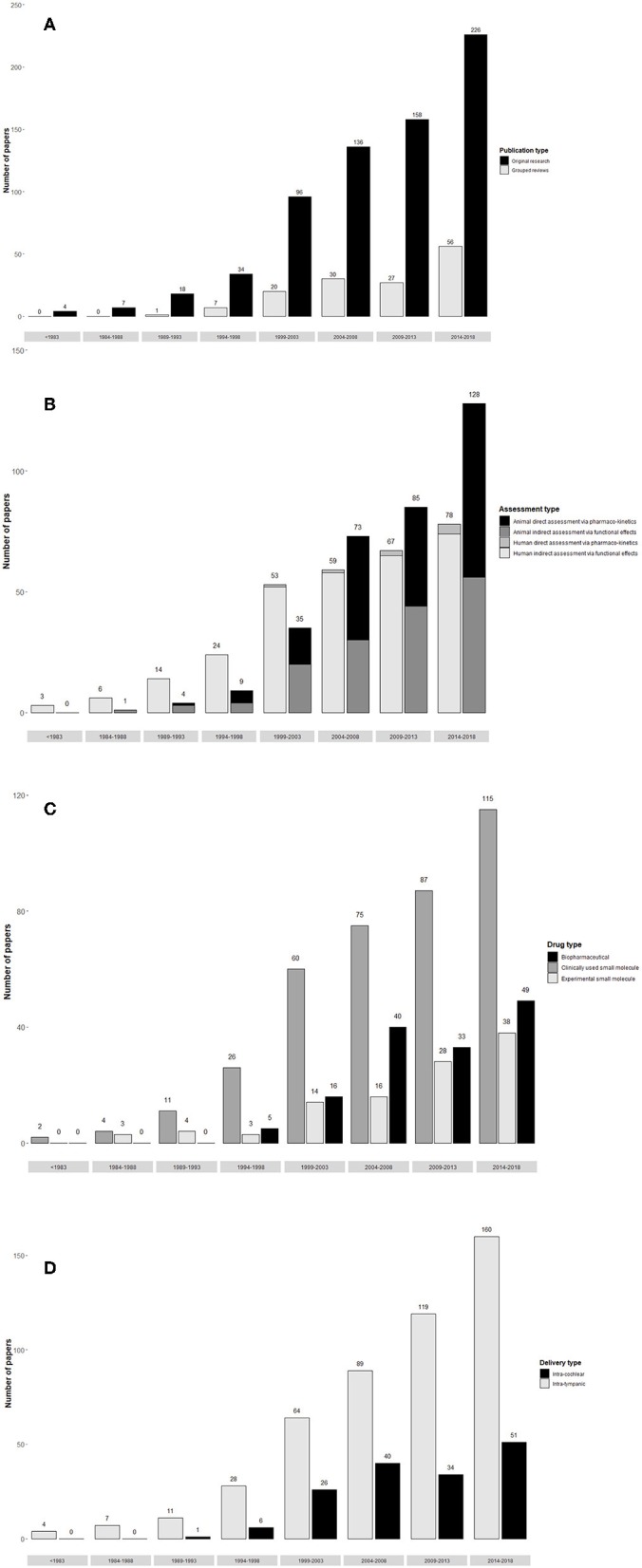
Trends in publication of studies on local delivery to the inner ear. **(A)** Publications per 5 years by study type (original research or review). **(B)** Original research publications of per 5 years by method of assessment of delivery efficacy (direct assessment via pharmacokinetics or indirect assessment via functional effects, excluding feasibility studies where delivery efficacy is not assessed). **(C)** Original research publications per 5 years by intratympanic and intracochlea delivery routes in both humans and animals (excluding “other” delivery routes). **(D)** Original research publications per 5 years by therapeutic class (clinically used small molecule, experimental small molecule, or biopharmaceutical) in both humans and animals. [n.b. **(A–D)** runs from top to bottom of figure panel].

The annual number of publications in the field of local therapeutic delivery to the inner ear has increased steadily over the last three decades, with a sharp increase over the last 5 years (Figure 3A). Of the 679 original research articles 226 have been published in the last 5 years. Remarkably, there have been 56 reviews published in the last 5 years.

Trends in experimental design are seen in Figure 3B. There has been a sharp increase in the number of animal studies published, while human studies have increased more gradually. The early human work indirectly assessing delivery via functional effects relates almost exclusively to transtympanic injections of corticosteroids and aminoglycosides. Direct assessment of delivery methods via pharmacokinetics accounts for around half of animal studies year on year.

Trends in delivery routes are seen in Figure 3C. Transtympanic delivery methods outnumber intracochlear methods year on year.

The trend in category of therapeutic administered is shown in Figure 3D. Clinically used small molecules (corticosteroids and aminoglycosides) account for the majority of publications each year, the majority of which are case series. The number of papers delivering experimental small molecules and biopharmaceuticals has increased steadily, reflecting development in the field.

### Delivery Methods

The delivery methods used in original research papers identified by this study are seen in Table 6, with numbers of publications organized by therapeutic formulation and experimental model. An identical table is found in Supplementary Material giving the reference numbers of papers referred to within each cell of the table (Supplementary Table 1).

**Table 6:** Total numbers of original studies for each delivery method arranged by formulation, in humans (living and temporal bone models), and animals.

**Delivery**			**Formulation**									**Total**
**Route**	**Approach**	**Delivery method**	**Solution**		**Sustained release**		**Nano-scale**		**Viral vector**		**Other/multiple**	
			**Human**	**Animal**	**Human**	**Animal**	**Human**	**Animal**	**Human**	**Animal**		
Intra-tympanic	Non-surgical	Transtympanic Injection	203	63	7	14	–	10	–	–	2	299
		Tympanostomy Tube	6	–	11	–	–	–	–	–	–	17
	Surgical	Bullostomy (animal only)	–	1	–	2	–	2	–	–	–	5
		Micropump or catheter	27	7	–	–	–	2	–	–	2	38
		Endoscope-assisted	–	–	–	–	–	–	–	–	–	0
		Stapes surgery	–	1	–	2	–	–	–	–	–	3
	Other		2	15	10	30	1	16	–	–	4	78
	Multiple		27	4	4	1	–	1	–	–	5	42
Intra-cochlear/labyrinthine	Round or oval window	Injection	1	17	–	1	–	1	–	26	–	46
		Stapes surgery	–	–	–	–	–	–	–	–	–	0
		Micropump or catheter	–	32	–	–	–	–	–	–	–	32
		Cochlear implant	–	2	–	7	–	–	–	–	–	9
	Cochleostomy	Injection	–	20	–	3	–	2	–	15	–	40
		Micropump or catheter	–	14	–	1	–	1	–	1	–	17
		Cochlear implant	–	–	–	1	–	–	–	–	–	1
	Canalostomy	Injection	5	4	–	–	–	–	–	8	–	17
	Endolymphatic sac	Injection	–	–	–	–	–	–	–	–	–	0
	Other		–	–	–	1	–	–	–	–	–	1
	Multiple		4	8	–	–	–	–	–	–	–	12
Other		Iontophoresis	1	1	–	–	–	–	–	–	–	2
		Post-auricular injection	1	–	–	–	–	–	–	–	–	1
Combination				6	–	4	–	5	–	–	4	19

*This table is mirrored by Supplementary Table 1, in which cells contain reference numbers of each study in place of total numbers*.

### Reference Tables

Cross-referenced tables have been formed to allow identification of papers looking at any combination of delivery method, therapeutic class/formulation, and disease model. Reviews are tabulated separately. All are available in Supplementary Material.

## Discussion

### Summary of Main Findings

In this review we provide a comprehensive overview of all research activity in the field of local therapeutic delivery to the inner ear, and show how this activity has increased over the last two decades. Though our categorization systems, we reveal how delivery methods are developed and assessed, and our cross referenced tables highlight the complexity in the way that delivery methods combine with formulations and therapeutic agents.

Categorization of the studies by method of assessment of delivery has exposed for the first time a relative lack of research into direct delivery efficacy, especially in humans, with most studies looking at clinical efficacy. Direct assessment of delivery efficacy is critical to understanding how to deliver therapeutics. Without this understanding, it becomes impossible to tell if an absence of a clinical effect is due to therapeutic failure or delivery failure, which has significant implications for both pre-clinical work and trials of novel therapeutics. Given the difficulties in assessing delivery directly in humans (found in this review to be limited to imaging of compounds with a contrast effect, or tissue/perilymph sampling alongside operative management of a pathological ear), more work is needed into understanding how the animal work in this area translates to humans. Computer modeling has potential to assist with this but at present is limited to interpretation of experimental data (Salt, 2002).

Original research outputs have increased steeply over the last decade, reflecting increased interest and funding into hearing. Animal models have been used in over half of the original research studies included in this systematic review, with a growing trend. This reflects the increasing number of novel therapeutics in pre-clinical development, and to some extent, the challenges of locally delivering therapeutics in humans. Whilst animal models play a vital role in the development of delivery methods, and the testing of novel inner ear therapeutics (Frisina et al., 2018), their applicability to humans is unclear (Denayer et al., 2014; Le Prell et al., 2016; Frisina et al., 2018). There remain significant challenges in translating from animals to humans due to differences in anatomy (thinner bone overlying cochlea; Mikulec et al., 2009), size (smaller cochlea allows greater diffusion; Salt, 2008), and disease models (ISSNHL difficult to replicate).

There is a significant body of literature on aminoglycosides and steroids as intra-tympanic treatments for Meniere's disease and ISSNHL, respectively. Whilst this is expected for treatments in clinical use, the large number of papers that continue to be published investigating the efficacy of these drugs suggests that either published information is not of high enough quality to draw conclusions, or is not being disseminated widely enough. In either case, it emphasizes the need for appropriately powered studies with accessible results as novel therapeutics move to trials and beyond.

This review has gone beyond previous systematic reviews in the field, which have focused on assessing the efficacy of single delivery techniques and therapeutic agents in humans. Here we have mapped the large number of delivery methods available, which have been tested with a range of therapeutic agents and formulations, in order to signpost readers from different backgrounds to the information most relevant to them. Intra-tympanic delivery routes dominate the field. This in large part reflects clinical acceptance of intra-tympanic injections resulting from the work described above. Furthermore, small molecules account for the majority of therapeutics delivered locally to the inner ear (447/680, 65.7%), and these agents are mostly suitable for intra-tympanic administration. Intra-cochlear delivery, whilst currently almost essential for cell and gene based therapeutic modalities, carries much higher risks, and has only recently started to be tested in humans (Nakagawa and Ito, 2005)^1^. The observed imbalance in research across these delivery routes therefore indicates how the field has developed, and is developing, rather than representing a gap in research into intra-cochlear delivery methods.

### Limitations

Some limitations of this study merit discussion. Firstly, we did not set out to extract data on the methodological quality, or to extract and meta-analyze results of individual publications. We did not therefore aim to make recommendations on which delivery method should be used for a given experimental model, therapeutic, or disease, although we do enable studies containing this information to be identified.

Secondly, we did not include gray literature in this paper. In a fast moving field, this inevitably means we have not captured ongoing, or very recently completed studies.

Finally, the extraction of data about delivery method and formulation is highly complex, with multiple sub-categories required (e.g., intra-tympanic injection, through tympanostomy tube, of hydrogel) to ensure the exact nature of a delivery method was captured. This opens the possibility of misclassification, which we mitigated by ensuring two authors extracted all data independently, and that any discrepancies or difficult classifications were discussed within the review team.

## Conclusions

Research into local therapeutic delivery to the inner ear has undergone a recent surge, improving our understanding of how novel therapeutics can be delivered. The way in which a therapeutic should be delivered, however, remains somewhat elusive, limited by the difficulty in directly researching the efficacy of delivery to the human inner ear.

The majority of research so far focuses on clinical efficacy of administered therapies. Our knowledge about the efficacy of delivery methods in humans remains limited, and this has the potential to limit how we assess the effectiveness of novel therapeutics.

As the field of inner ear therapeutics develops, it is crucial that research into delivery methods considers both the relationships between therapeutic agent, formulation, delivery method and disease, and the translational challenges that exist when moving from animal to human work. Collaboration between lab scientists, computer scientists, clinicians, industry, and patients will be key to overcoming these challenges, and tailoring delivery methods to novel therapeutics to maximize the chances of success in clinical trials.

## Data Availability

All datasets generated for this study are included in the manuscript/Supplementary Files.

## Author Contributions

CA, CX, HB, and AS designed the study. CX ran the systematic searches. CA, CX, MS, MG, and HB determined study inclusion/exclusion and extracted data. CA, CX, and MS performed data analysis. CA, CX, MS, MG, HB, and AS wrote the manuscript.

### Conflict of Interest Statement

The authors declare that the research was conducted in the absence of any commercial or financial relationships that could be construed as a potential conflict of interest.

## Footnotes

^1^*Safety, Tolerability and Efficacy for CGF166 in Patients With Unilateral or Bilateral Severe-to-profound Hearing Loss—Full Text View—**ClinicalTrials.gov*. Available online at: https://clinicaltrials.gov/ct2/show/NCT02132130 (accessed July 20, 2018).

^2^*ISRCTN-ISRCTN59733689: A First-in-Human Study of the Safety and Efficacy of a New Drug, a Gamma Secretase Inhibitor, to Treat People With Sensorineural Hearing Loss*. Available online at: http://www.isrctn.com/ISRCTN59733689 (accessed December 23, 2018).

^3^*Documentation and Sources-DrugBank*. Available online at: https://www.drugbank.ca/documentation#drug-cards (accessed January 26, 2019).
