# Supplementary material for: Local Delivery of Therapeutics to the Inner Ear: The State of the Science
Source: Front Cell Neurosci. 2019 Oct 9;13:418. doi: 10.3389/fncel.2019.00418 (PMC6794458; doi:10.3389/fncel.2019.00418)
Supplement: Supplementary file 1 [file Table_1.DOCX]

Supplementary Material

# Supplementary Data

Search Strategies

**EMBASE**:

1. exp inner ear/

2. (inner adj3 ear).mp. [mp=title, abstract, heading word, drug trade name, original title, device manufacturer, drug manufacturer, device trade name, keyword, floating subheading word]

3. (cochlea or cochlear).mp. [mp=title, abstract, heading word, drug trade name, original title, device manufacturer, drug manufacturer, device trade name, keyword, floating subheading word

4. (cochleas or cochleas or cochleae).mp. [mp=title, abstract, heading word, drug trade name, original title, device manufacturer, drug manufacturer, device trade name, keyword, floating subheading word]

5. hair cell$.mp. [mp=title, abstract, heading word, drug trade name, original title, device manufacturer, drug manufacturer, device trade name, keyword, floating subheading word]

6. drug therapy/ or drug tissue level/ or drug transport/ or drug vehicle/

7. drug delivery device/ or drug delivery system/

8. (drug$ adj3 deliver$).mp. [mp=title, abstract, heading word, drug trade name, original title, device manufacturer, drug manufacturer, device trade name, keyword, floating subheading word]

9. (drug$ adj3 carr$).mp. [mp=title, abstract, heading word, drug trade name, original title, device manufacturer, drug manufacturer, device trade name, keyword, floating subheading word]

10. biological therapy/

11. gene therapy/

12. cell therapy/

13. (gene$ adj3 therap$).mp. [mp=title, abstract, heading word, drug trade name, original title, device manufacturer, drug manufacturer, device trade name, keyword, floating subheading word]

14. (cell$ adj3 therap$).mp. [mp=title, abstract, heading word, drug trade name, original title, device manufacturer, drug manufacturer, device trade name, keyword, floating subheading word

15. (tissue$ adj3 therap$).mp. [mp=title, abstract, heading word, drug trade name, original title, device manufacturer, drug manufacturer, device trade name, keyword, floating subheading word

16. (intra-tympanic or intratympanic).mp. [mp=title, abstract, heading word, drug trade name, original title, device manufacturer, drug manufacturer, device trade name, keyword, floating subheading word]

17. (trans-tympanic or transtympanic).mp. [mp=title, abstract, heading word, drug trade name, original title, device manufacturer, drug manufacturer, device trade name, keyword, floating subheading word]

18. (intra-cochlea$ or intracochlea$).mp. [mp=title, abstract, heading word, drug trade name, original title, device manufacturer, drug manufacturer, device trade name, keyword, floating subheading word]

19. 1 or 2 or 3 or 4 or 5

20. 6 or 7 or 8 or 9 or 10 or 11 or 12 or 13 or 14 or 15 or 16 or 17 or 18

21. 19 and 20

22. hearing impairment/dt [Drug Therapy]

23. perception deafness/dt [Drug Therapy]

24. Meniere disease/dt [Drug Therapy]

25. vestibular disorder/dt [Drug Therapy]

26. sudden deafness/dt [Drug Therapy]

27. tinnitus/dt [Drug Therapy]

28. 22 or 23 or 24 or 25 or 26 or 27

29. 21 or 28

30. limit 29 to english language

**PubMed:**

1. exp Ear, Inner/

2. (inner adj3 ear).mp. [mp=title, abstract, original title, name of substance word, subject heading word, keyword heading word, protocol supplementary concept word, rare disease supplementary concept word, unique identifier, synonyms]

3. (cochlea or cochlear).mp. [mp=title, abstract, original title, name of substance word, subject heading word, keyword heading word, protocol supplementary concept word, rare disease supplementary concept word, unique identifier, synonyms]

4. (cochleas or cochlears or cochleae).mp. [mp=title, abstract, original title, name of substance word, subject heading word, keyword heading word, protocol supplementary concept word, rare disease supplementary concept word, unique identifier, synonyms]

5. hair cell$.mp. [mp=title, abstract, original title, name of substance word, subject heading word, keyword heading word, protocol supplementary concept word, rare disease supplementary concept word, unique identifier, synonyms]

6. Pharmaceutical Preparations/ or Delayed-Action Preparations/ or Nanostructures/ or Nanoparticles/ or Polymers/ or Drug Carriers/ or Drug Delivery Systems/ or drug delivery.mp. or Liposomes/

7. (drug$ adj3 deliver$).mp. [mp=title, abstract, original title, name of substance word, subject heading word, keyword heading word, protocol supplementary concept word, rare disease supplementary concept word, unique identifier, synonyms]

8. (drug$ adj3 carr$).mp. [mp=title, abstract, original title, name of substance word, subject heading word, keyword heading word, protocol supplementary concept word, rare disease supplementary concept word, unique identifier, synonyms]

9. biological therapy/ or "cell- and tissue-based therapy"/ or cell transplantation/ or tissue transplantation/ or genetic therapy/ or rnai therapeutics/ or targeted gene repair/

10. (gene$ adj3 therap$).mp. [mp=title, abstract, original title, name of substance word, subject heading word, keyword heading word, protocol supplementary concept word, rare disease supplementary concept word, unique identifier, synonyms]

11. (cell$ adj3 therap$).mp. [mp=title, abstract, original title, name of substance word, subject heading word, keyword heading word, protocol supplementary concept word, rare disease supplementary concept word, unique identifier, synonyms]

12. (tissue$ adj3 therap$).mp. [mp=title, abstract, original title, name of substance word, subject heading word, keyword heading word, protocol supplementary concept word, rare disease supplementary concept word, unique identifier, synonyms]

13. (intra-tympanic or intratympanic).mp. [mp=title, abstract, original title, name of substance word, subject heading word, keyword heading word, protocol supplementary concept word, rare disease supplementary concept word, unique identifier, synonyms]

14. (trans-tympanic or transtympanic).mp. [mp=title, abstract, original title, name of substance word, subject heading word, keyword heading word, protocol supplementary concept word, rare disease supplementary concept word, unique identifier, synonyms]

15. (intra-cochlea$ or intracochlea$).mp. [mp=title, abstract, original title, name of substance word, subject heading word, keyword heading word, protocol supplementary concept word, rare disease supplementary concept word, unique identifier, synonyms]

16. exp Hearing/de, pd [Drug Effects, Pharmacology]

17. Ear, Inner/de, pd [Drug Effects, Pharmacology]

18. Cochlear Nerve/de, pd [Drug Effects, Pharmacology]

# Supplementary Figures and Tables

Table 1: **References** of original studies for each delivery method arranged by category of formulation, in human and animal models.

| **Delivery** | | | **Formulation** | | | | | | | |  | **Total Number** |
| --- | --- | --- | --- | --- | --- | --- | --- | --- | --- | --- | --- | --- |
| **Route** | **Approach** | **Delivery Method** | **Solution** | | **Sustained Release** | | **Nano-scale** | | **Viral Vector** | | **Other/ Multiple** |  |
|  |  |  | **Human** | **Animal** | **Human** | **Animal** | **Human** | **Animal** | **Human** | **Animal** |  |  |
| Intra-tympanic | Non-surgical | Transtympanic Injection | 7 8 11 19 20 26 32 37 42 43 45 46 47 48 49 59 60 61 62 76 77 78 97 98 99 111 112 113 114 115 132 133 134 135 136 137 138 139 151 152 153 154 155 156 157 158 170 171 172 173 175 176 190 191 192 193 195 196 197 198 199 200 201 217 218 219 220 222 223 224 226 227 228 252 253 255 256 257 258 259 260 261 262 263 264 288 290 291 292 293 294 319 320 321 322 323 324 325 346 348 350 351 352 353 355 356 357 360 386 387 390 391 392 393 394 395 397 398 399 400 401 402 404 405 406 407 408 419 420 421 425 439 441 444 445 446 447 448 449 450 452 462 463 464 465 466 467 468 469 488 489 490 491 492 493 494 495 496 497 498 500 501 527 528 529 533 534 535 536 537 581 582 583 585 586 588 589 590 591 593 594 595 596 598 600 601 604 606 610 647 648 652 653 654 655 656 659 661 662 663 665 667 670 | 9 15 174 194 221 225 229 254 265 289 347 349 354 358 359 361 388 389 396 403 409 416 418 422 423 424 440 442 443 451 499 530 531 532 539 584 587 592 597 599 602 603 605 607 608 609 611 612 649 650 651 657 658 664 666 668 669 674 672 673 674 675 676 | 417 460 461 485 575 576 577 | 54 71 74 318 345 383 384 385 486 519 523 524 574 660 | - | 487 525 526 568 578 579 643 644 645 646 | - | - | 437 538 | 299 |
|  |  | Tympanostomy Tube | 16 38 79 118 267 471 | - | 146 209 210 230 266 282 326 327 362 363 427 | - | - | - | - | - | - | 17 |
|  | Surgical | Bullostomy (animals) | - | 382 | - | 518 637 | - | 570 640 | - | - | - | 5 |
|  |  | Micropump or Catheter | 1 10 18 23 24 29 31 55 56 92 93 94 105 106 107 108 125 126 169 187 211 311 338 339 435 481 634 | 12 109 147 283 312 375 567 | - | - | - | 457 566 | - | - | 89 414 | 38 |
|  |  | Endoscope-assisted | - | - | - | - | - | - | - | - | - | 0 |
|  |  | Stapes Surgery | - | 571 | 3 | 2 480 | - | - | - | - | - | 4 |
|  | Other | | 3 73 | 36 57 95 148 213 214 215 216 315 317 459 572 573 641 642 | 4 5 6 53 127 343 456 458 563 569 | 52 128 168 188 247 249 280 285 286 287 309 310 313 314 340 341 344 377 378 379 432 433 483 517 520 521 522 564 565 632 | 376 | 212 248 250 284 380 381 431 479 482 484 561 633 635 636 638 639 | - | - | 103 281  342 436 | 78 |
|  | Multiple | | 25 35 50 58 75 90 91 96 116 117 129 131 149 150 189 202 203 231 232 233 316 328 410 415 470 502 580 | 110 251 426 438 | 34 70 72 186 | 562 | - | 337 | - | - | 41 63 130 246 434 | 42 |
| Intra-Cochlear/ Labyrinthine | Round or Oval Window | Injection | 628 | 83 142 164 182 183 206 243 430 514 553 554 555 559 560 626 627 629 | - | 370 | - | 511 | - | 44 66 67 82 161 162 163 205 240 241 242 273 300 334 429 478 512 548 549 550 551 552 558 624 625 631 | - | 46 |
|  |  | Stapes Surgery | - | - | - | - | - | - | - | - | - | 0 |
|  |  | Micropump or Catheter | - | 28 30 33 39 69 85 86 87 102 121 122 123 124 143 144 145 166 167 185 207 208 244 245 276 279 302 303 373 515 516 556 557 | - | - | - | - | - | - | - | 32 |
|  |  | Cochlear Implant | - | 120 268 | - | 236 365 474 475 505 543 620 | - | - | - | - | - | 9 |
|  | Cochleostomy | Injection | - | 88 101 180 181 204 237 239 272 298 299 333 369 411 476 509 510 545 546 621 622 | - | 454 544 623 | - | 308 506 | - | 64 81 160 238 270 271  336 367 374 368 412 413 455 507 508 | - | 40 |
|  |  | Micropump or Catheter | - | 51 68 84 274 275 277 278 304 305 306 307 335 366 372 477 | - | - | - | 65 | - | 40 | - | 17 |
|  |  | Cochlear Implant | - | - | - | 504 | - | - | - | - | - | 1 |
|  | Canalostomy | Injection | 14 17 22 27 80 | 21 234 295 616 | - | - | - | - | - | 329 472 613 614 615 677 678 679 | - | 17 |
|  | Endolymphatic Sac | Injection | - | - | - | - | - | - | - | - | - | 0 |
|  | Other | | - | - | - | 630 | - | - | - | - | - | 1 |
|  | Multiple | | 141 296 371 513 | 165 184 297 301 332 428 542 547 | - | - | - | - | - | - | - | 12 |
| Other |  | Iontophoresis | 13 | 159 | - | - | - | - | - | - |  | 2 |
|  |  | Post-auricular injection | 453 | - | - | - | - | - | - | - |  | 1 |
| Combination |  |  | - | 119 140 503 540 617 618 | - | 177 178 235 473 | - | 100 269 330 364 619 | - | - | 104 179 331 541 | 19 |

Table 2: **References** of original studies for each delivery method arranged by method of assessment of delivery, in human and animal models (includes multiples).

| **Delivery Route** | **Approach** | **Delivery Method** | **Direct assessment of efficacy via pharmacokinetics** | | **Indirect assessment of efficacy via functional effects** | | **Feasibility** | | **Total Number** |
| --- | --- | --- | --- | --- | --- | --- | --- | --- | --- |
|  |  |  | **Human** | **Animal** | **Human** | **Animal** | **Human** | **Animal** |  |
| Intra-tympanic | Non-surgical | Transtympanic Injection | 264 360 401 601 604 606 670 | 74 225 254 318 345 349 383 384 385 388 403 416 440 442 486 487 525 526 568 579 602 644 645 646 649 651 664 668 672 676 | 7 8 11 19 20 26 32 37 42 43 45 46 47 48 49 59 60 61 62 76 77 78 97 98 99 111 112 113 114 115 132 133 134 135 136 137 138 139 151 152 153 154 155 156 157 158 170 171 172 173 175 176 190 191 192 193 195 196 198 199 200 201217 218 219 220 222 223 224 226 227 228 252 253 255 256 257 258 259 260 261 262 263 288 290 291 292 293 294 319 320 321 322 323 324 325 346 348 350 351 352 353 355 356 357 386 387 390 391 392 393 394 395 397 398 399 400 402 404 405 406 407 408 417 419 420 421 425 437 439 441 444 445 446 447 448 449 450 452 460 461 462 463 464 465 466 467 468 469 485 488 489 490 491 492 493 494 495 496 497 498 500 501 527 528 529 533 534 535 536 537 575 577 581 582 583 585 586 588 589 590 591 593 595 596 598600 610 647 648 652 653 654 655 656 659 661 662 663 665 667 | 9 15 54 71 174 194 221 265 289 347 354 358 359 361 389 396 409 418 422 423 424 443 499 523 524 530 531 532 538 539 574 578 584 587 592 597 599 603 605 607 608 609 611 612 650 657 658 660 669 671 673 675 | 197 576 594 | 229 451 519 643 666 674 | 299 |
|  |  | Tympanostomy Tube | - | - | 16 38 79 118 146 209 210 230 266 267 282 326 327 362 363 427 471 | - | - | - | 17 |
|  | Surgical | Bullostomy (animals) | - | 637 640 | - | 382 518 570 | - | - | 5 |
|  |  | Micropump or Catheter | -- | 283 375 559 560 566 | 1 10 18 23 24 29 31 55 56 92 93 94 105 106 107 108 125 126 169 187 311 338 339 435 481 634 | 12, 89 147 312 414 | 211 | 109 457 567 | 38 |
|  |  | Endoscope-assisted | - | - | - | - | - | - | 0 |
|  |  | Stapes Surgery | - | 480 571 | - | - | - | 2 | 3 |
| Intra-Cochlear/ Labyrinthine | Round or Oval Window | Injection | - | 44 66 162 163 164 182 183 206 240 241 243 273 300 334 429 430 511 514 548 550 555 558 624 626 627 629 | 628 | 67 82 142 161 205 242 370 478 512 549 551 553 554 631 | - | 83 552 625 | 46 |
|  |  | Stapes Surgery | - | - | - | - | - | - | 0 |
|  |  | Micropump or Catheter | - | 28 30 121 185 207 276 373 515 516 | - | 33 39 69 85 86 102 122 123 124 143 144 145 166 167 208 244 245 279 302 303 557 | - | 87 556 | 32 |
|  |  | Cochlear Implant | - | 120 365 474 475 505 543 | - | 236 268 620 | - | - | 9 |
|  | Cochleostomy | Injection | - | 64 160 180 204 237 238 239 270 271 272 298 299 308 336 367 455 476 507 508 510 544 545 546 622 | - | 81 101 181 333 368 369 374 411 412 413 454 509 | - | 88 506 621 623 | 40 |
|  |  | Micropump or Catheter | - | 40 51 65 274 305 307 477 | - | 84 275 277 278 304 306 335 366 372 | - | 68 | 17 |
|  |  | Cochlear Implant | - | 504 | - | - | - | - | 1 |
|  | Canalostomy | Injections | - | 234 295 472 613 614 677 678 679 | 14 17 22 27 80 | 21 329 615 616 | - | - | 17 |
| Other |  | Iontophoresis | - | 159 | 13 | - | - | - | 2 |
|  |  | Post-auricular injection | - | 453 | - | - | - | - | 1 |

Table 3: **References** of original studies for each therapeutic category arranged by disease.

| **Therapeutic Category** | **Subcategory** | **Class** | **Disease model** | | | | | | | |
| --- | --- | --- | --- | --- | --- | --- | --- | --- | --- | --- |
|  |  |  | **Meniere’s Disease** | **SSNHL** | **Ototoxicity** | **CI outcome improvement** | **Autoimmune inner ear disease** | **Genetic** | **Tinnitus** | **NIHL** |
| **Clinically used small molecules** |  | Corticosteroids | 53, 58, 79, 97, 117, 157, 176, 193, 291, 327, 356, 417, 441, 481, 494, 495, 498, 500, 536, 537, 541, 588, 589, 591, 593, 595, 661 | 1, 3, 5, 6, 11, 16, 17, 19, 21, 22, 23, 24, 27, 29, 31, 32, 35, 37, 38, 45, 46, 47, 48, 50, 55, 56, 59, 60, 62, 70, 73, 75, 76, 77, 78, 80, 90, 91, 92, 93, 94, 96, 105, 106, 107, 111, 112, 113, 125, 127, 130, 131, 133, 134, 135, 136, 137, 138, 146, 149, 151, 152, 153, 154, 155, 156, 169, 170, 171, 172, 173, 189, 190, 191, 192, 209, 210, 217, 218, 219, 220, 231, 232, 252, 253, 255, 256, 257, 258, 267, 288, 319, 320, 326, 328, 346, 362, 386, 387, 435, 439, 462, 463, 464, 488, 489, 490, 491, 527, 528, 529, 580, 581, 582, 647 | 123, 265, 289, 389, 396, 422, 424, 443, 482, 530, 578, 587, 592, 599, 639, 640, 643, 658, 667 | 235, 264, 275, 280, 296, 309, 335, 401, 432, 523, 542, 561, 600, 617, 620, 628 | - | - | 42, 49, 98, 139, 158, 197, 198, 324, 325, 391, 467, 585, 652 | 194, 208, 423, 436, 499, 524, 531, 556, 574, 635, 657, 660 |
|  |  | Aminoglycosides | 108, 114, 116, 126, 175, 186, 187, 196, 199, 200, 202, 203, 211, 223, 224, 226, 227, 228, 233, 260, 261, 262, 263, 266, 290, 292, 293, 294, 311, 321, 322, 323, 338, 348, 350, 351, 352, 353, 355, 357, 390, 392, 393, 394, 395, 397, 398, 399, 400, 402, 404, 405, 406, 410, 415, 419, 420, 427, 434, 437, 444, 445, 446, 447, 448, 449, 450, 458, 465, 466, 468, 470, 471, 492, 493, 497, 502, 533, 534, 535, 563, 569, 575, 586, 590, 596, 598, 634, 653, 654, 655, 659, 662, 663, 665 | - | - |  | - | - |  | - |
| **Experimental small molecules** | Therapeutic agents | Local anaesthetics | 7, 8, 150, 407 | - | - |  | - | - | 13, 25, 43, 115 | - |
|  |  | Bisphosphonates | - | - | 143, 148, 174, 247, 313, 347, 584 |  | - | - | - | - |
|  |  | Antioxidants | - | - | - |  | - | - | - | 484, 650 |
|  |  | Antivirals | 282 | - | - |  | - | - | - | - |
|  |  | Apoptosis Inhibitors | - | 460 | - |  | - | - | - | 122, 246, 259, 372, 382 |
|  |  | NMDA receptor antagonists |  | - | - |  | - | - | 339, 408, 461, 485, 576 | - |
|  | Toxic agents |  |  | - | - |  | - | - | - | - |
|  | Contrast media & dyes |  | 602, 604, 606 | - | - | 379 | 671 | - | - | - |
| **Biopharmaceuticals** | Protein based therapies | Neurotrophins | - | 343 | 33, 39, 124, 166, 167, 178, 179, 180, 244, 245, 268, 278, 279, 303, 304, 312, 333, 475, 557 | 564, 623 | - | - | - | 86, 128, 314, 518 |
|  |  | Monoclonal antibodies | - | - | - |  | 230, 469 | - | - | - |
|  | Gene correction therapies |  | - | - | 44, 64, 67, 81, 82, 160, 161, 205, 240, 242, 298, 329, 336, 358, 367, 413, 551, 631 | - | - | 411, 412, 455, 478, 512, 546, 549, 550, 558, 560, 615, 624, 629, 679 | - | 101, 369, 374, 438 |
|  | Cell therapies |  | - | - | 183, 236, 295, 509, 510, 543, 545, 553, 554 | - | - | 627 | - | 626 |
| **Other** |  |  | 4, 10, 145, 201 | 456 | 409, 483, 538, 539, 573, 577, 668, 673, 674, 675 | - | 144 | 619 | - | 181, 310, 430, 616 |
| **Multiple** |  |  | 14, 18, 20, 26, 34, 118, 425, 452, 501, 610 | - | 359, 361, 414, 570, 676 | - | 89 | - | 63, 99, 287 | - |
| **None** |  |  | - | - | - | - | - | - | - | 613 |

Table 4: **References** of original studies for each therapeutic category arranged by study model.

| **Therapeutic Category** | **Subcategory** | **Class** | **References** | | **Total Number** |
| --- | --- | --- | --- | --- | --- |
|  |  |  | **Human** | **Animal** |  |
| **Clinically used small molecules** |  | Corticosteroids | 41, 42, 49, 53, 58, 79, 97, 98, 108, 114, 116, 117, 126, 139, 157, 158, 175, 176, 186, 187, 193, 195, 196, 197, 198, 199, 200, 202, 203, 211, 222, 223, 224, 226, 227, 228, 233, 260, 261, 262, 263, 264, 266, 290, 291, 292, 293, 294, 296, 311, 321, 322, 323, 324, 325, 327, 338, 348, 350, 351, 352, 353, 355, 356, 357, 363, 390, 391, 392, 393, 394, 395, 397, 398, 399, 400, 401, 402, 404, 405, 406, 410, 415, 417, 419, 420, 421, 427, 434, 437, 441, 444, 445, 446, 447, 448, 449, 450, 458, 465, 466, 467, 468, 470, 471, 481, 492, 493, 494, 495, 496, 497, 498, 500, 502, 533, 534, 535, 536, 537, 541, 563, 569, 575, 585, 586, 588, 589, 590, 591, 593, 594, 595, 596, 598, 600, 628, 634, 652, 653, 654, 655, 656, 659, 661, 662, 663, 665, 667 | 57, 85, 95, 123, 188, 194, 207, 208, 213, 214, 225, 235, 265, 275, 276, 280, 281, 285, 289, 309, 315, 318, 335, 349, 354, 378, 383, 384, 385, 389, 396, 403, 422, 423, 424, 432, 436, 442, 443, 454, 474, 480, 482, 499, 504, 505, 517, 521, 522, 523, 524, 530, 531, 532, 542, 556, 561, 574, 578, 587, 592, 597, 599, 617, 620, 630, 632, 633, 635, 639, 640, 641, 643, 644, 651, 657, 658, 660, 664, 666 | 229 |
|  |  | Aminoglycosides | 1, 3, 5, 6, 11, 16, 17, 19, 22, 23, 24, 27, 29, 31, 32, 35, 37, 38, 45, 46, 47, 48, 50, 55, 56, 59, 60, 61, 62, 70, 73, 75, 76, 77, 78, 80, 90, 91, 92, 93, 94, 96, 105, 106, 107, 111, 112, 113, 125, 127, 130, 131, 132, 133, 134, 135, 136, 137, 138, 146, 149, 151, 152, 153, 154, 155, 156, 169, 170, 171, 172, 173, 189, 190, 191, 192, 209, 210, 217, 218, 219, 220, 231, 232, 252, 253, 255, 256, 257, 258, 267, 288, 319, 320, 326, 328, 346, 362, 386, 387, 435, 439, 462, 463, 464, 488, 489, 490, 491, 527, 528, 529, 580, 581, 582, 583, 647, 648 | 12, 21, 52, 54, 71, 74, 84, 103, 110, 120, 168, 251, 254, 302, 340, 341, 440, 503, 571, 649 | 138 |
| **Experimental small molecules** | Therapeutic agents | Local anesthetics | 7, 8, 13, 25, 43, 115, 150, 407 | 15, 215, 229, 342 | 12 |
|  |  | Bisphosphonates | 513 | - | 1 |
|  |  | Antioxidants | - | 121, 143, 148, 174, 221, 247, 313, 347, 388, 484, 584, 650 | 12 |
|  |  | Antivirals | 282 | 459 | 2 |
|  |  | Apoptosis Inhibitors | 259, 460 | 122, 246, 372, 377, 382, 520 | 8 |
|  |  | NMDA receptor antagonists | 339, 408, 461, 485, 576 | 477, 515 | 7 |
|  | Toxic agents |  | - | 28, 109, 605 | 3 |
|  | Contrast media & dyes |  | 360, 601, 604, 606, 670 | 30, 159, 216, 243, 283, 284, 331, 344, 345, 375, 379, 426, 453, 457, 479, 514, 568, 602, 611, 622 | 25 |
| **Biopharmaceuticals** | Protein based therapies | Neurotrophins | 343 | 33, 39, 86, 124, 128, 165, 166, 167, 177, 178, 179, 180, 184, 244, 245, 268, 277, 278, 279, 297, 301, 303, 304, 312, 314, 332, 333, 365, 366, 428, 475, 511, 518, 557, 564, 567, 623 | 38 |
|  |  | Monoclonal antibodies | 230, 469 | - | 2 |
|  | Gene correction therapies |  | - | 40, 44, 51, 64, 65, 66, 67, 81, 82, 88, 100, 101, 104, 119, 140, 160, 161, 162, 163, 205, 234, 237, 238, 240, 241, 242, 248, 270, 271, 273, 274, 298, 300, 329, 334, 336, 358, 367, 368, 369, 374, 411, 412, 413, 418, 429, 431, 438, 455, 472, 473, 478, 507, 508, 512, 546, 548, 549, 550, 551, 552, 558, 559, 560, 614, 615, 618, 624, 625, 629, 631, 677, 678, 679 | 74 |
|  | Cell therapies |  | - | 164, 182, 183, 204, 206, 236, 239, 272, 295, 299, 370, 476, 509, 510, 543, 544, 545, 553, 554, 621, 626, 627 | 22 |
| **Other** |  |  | 4, 10, 201, 371, 456, 577 | 2, 9, 36, 69, 102, 144, 145, 147, 181, 249, 305, 306, 307, 310, 373, 409, 416, 430, 433, 451, 483, 487, 516, 526, 538, 539, 565, 573, 607, 609, 616, 619, 636, 638, 668, 669, 671, 672, 673, 674, 675 | 47 |
| **Multiple** |  |  | 14, 18, 20, 26, 34, 63, 72, 99, 118, 425, 452, 501, 610 | 89, 142, 185, 287, 317, 359, 361, 414, 519, 555, 570, 603, 608, 612, 637, 676 | 29 |
| **None** |  |  | 129, 141, 316, 376 | 68, 83, 87, 212, 250, 269, 286, 308, 330, 337, 364, 380, 381, 486, 506, 525, 540, 547, 562, 566, 572, 579, 613, 642, 645, 646 | 30 |

Table 5:  **References** of original studies for each disease model arranged by study model.

| **Disease Model** | **References** | | **Total Number** |
| --- | --- | --- | --- |
|  | **Human** | **Animal** |  |
| **Meniere’s Disease** | 1, 3, 4, 5, 6, 7, 8, 10, 11, 14, 16, 17, 18, 19, 20, 22, 23, 24, 26, 27, 29, 31, 32, 34, 35, 37, 38, 45, 46, 47, 48, 50, 53, 55, 56, 58, 59, 60, 62, 70, 73, 75, 76, 77, 78, 79, 80, 90, 91, 92, 93, 94, 96, 97, 105, 106, 107, 111, 112, 113, 117, 118, 125, 127, 130, 131, 133, 134, 135, 136, 137, 138, 146, 149, 150, 151, 152, 153, 154, 155, 156, 157, 169, 170, 171, 172, 173, 176, 189, 190, 191, 192, 193, 201, 209, 210, 217, 218, 219, 220, 231, 232, 252, 253, 255, 256, 257, 258, 267, 282, 288, 291, 319, 320, 326, 327, 328, 346, 356, 362, 386, 387, 407, 417, 425, 435, 439, 441, 452, 462, 463, 464, 481, 488, 489, 490, 491, 494, 495, 498, 500, 501, 527, 528, 529, 536, 537, 541, 580, 581, 582, 588, 589, 591, 593, 595, 604, 606, 610, 647, 661 | 21, 145, 602 | 164 |
| **Sudden Sensorineural Hearing Loss** | 108, 114, 116, 126, 175, 186, 187, 196, 199, 200, 202, 203, 211, 223, 224, 226, 227, 228, 233, 260, 261, 262, 263, 266, 290, 292, 293, 294, 311, 321, 322, 323, 338, 343, 348, 350, 351, 352, 353, 355, 357, 390, 392, 393, 394, 395, 397, 398, 399, 400, 402, 404, 405, 406, 410, 415, 419, 420, 427, 434, 437, 444, 445, 446, 447, 448, 449, 450, 456, 458, 460, 465, 466, 468, 470, 471, 492, 493, 497, 502, 533, 534, 535, 563, 569, 575, 586, 590, 596, 598, 634, 653, 654, 655, 659, 662, 663, 665 |  | 98 |
| **Ototoxicity** | 577, 668 | 33, 39, 44, 64, 67, 81, 82, 123, 124, 143, 148, 160, 161, 166, 167, 174, 178, 179, 180, 183, 205, 236, 240, 242, 244, 245, 247, 265, 268, 278, 279, 289, 295, 298, 303, 304, 312, 313, 329, 333, 336, 347, 358, 359, 361, 367, 389, 396, 409, 413, 414, 422, 424, 443, 475, 482, 483, 509, 510, 530, 538, 539, 543, 545, 551, 553, 554, 557, 570, 573, 578, 584, 587, 592, 599, 631, 639, 640, 643, 658, 668, 673, 674, 675, 676 | 87 |
| **CI Outcome Improvement** | 264, 296, 401, 600, 628 | 235, 275, 280, 309, 335, 379, 432, 523, 542, 561, 564, 617, 620, 623 | 19 |
| **Autoimmune Inner ear disease** | 222, 230, 469, 671 | 89, 144 | 6 |
| **Genetic Hearing Loss** | - | 411, 412, 455, 478, 512, 546, 549, 550, 558, 560, 615, 619, 624, 627, 629, 679 | 16 |
| **Tinnitus** | 13, 25, 42, 43, 49, 63, 98, 99, 115, 139, 158, 197, 198, 324, 325, 339, 391, 408, 461, 467, 485, 576, 585, 652 | 287 | 25 |
| **NIHL** | 259 | 86, 101, 122, 128, 181, 194, 208, 246, 310, 314, 369, 372, 374, 382, 423, 430, 436, 438, 484, 499, 518, 524, 531, 556, 574, 613, 616, 626, 635, 650, 657, 660 | 33 |
| **Other** | 360, 496, 583, 648, 656 | 85, 102, 142, 182, 315, 354, 368, 377, 459, 472 | 15 |
| **Multiple** | 41, 61, 72, 132, 195, 363, 421, 594, 601 | 165, 184, 221, 277, 297, 301, 306, 332, 365, 370, 428 | 20 |
| **None** | 129, 141, 316, 371, 376, 513 | 2, 9, 12, 15, 28, 30, 36, 40, 51, 52, 54, 57, 65, 66, 68, 69, 71, 74, 83, 84, 87, 88, 95, 100, 103, 104, 109, 110, 119, 120, 121, 140, 147, 159, 162, 163, 164, 168, 177, 185, 188, 204, 206, 207, 212, 213, 214, 215, 216, 225, 229, 234, 237, 238, 239, 241, 243, 248, 249, 250, 251, 254, 269, 270, 271, 272, 273, 274, 276, 281, 283, 284, 285, 286, 299, 300, 302, 305, 307, 308, 317, 318, 330, 331, 334, 337, 340, 341, 342, 344, 345, 349, 364, 366, 373, 375, 378, 380, 381, 383, 384, 385, 388, 403, 416, 418, 426, 429, 431, 433, 440, 442, 451, 453, 454, 457, 473, 474, 476, 477, 479, 480, 486, 487, 503, 504, 505, 506, 507, 508, 511, 514, 515, 516, 517, 519, 520, 521, 522, 525, 526, 532, 540, 544, 547, 548, 552, 555, 559, 562, 565, 566, 567, 568, 571, 572, 579, 597, 603, 605, 607, 608, 609, 611, 612, 614, 618, 621, 622, 625, 630, 632, 633, 636, 637, 638, 641, 642, 644, 645, 646, 649, 651, 664, 666, 669, 671, 672, 677, 678 | 196 |

Table 6:  **References** of reviews arranged by study model.

| **Review Type** | **Animal models** | **Human models** | **Animal and human models** |
| --- | --- | --- | --- |
| **Narrative Review** | 4, 7, 8, 9, 10, 21, 28, 34, 36, 37, 42, 47, 49, 78, 93, 119, 12, 121, 123, 124, 136, 138, 141, 143, 145, 147, 148, 151, 158 | 3, 18, 19, 23, 26, 27, 30, 39, 41, 43, 50, 54, 56, 62, 63, 70, 73, 76, 83, 87, 89, 90, 94, 99, 100, 109, 129, 131, 132, 137, 139, 153, 154, 155, 156 | 1, 2, 5, 11, 14, 15, 16, 17, 20, 24, 25, 29, 31, 33, 35, 38, 40, 44, 46, 48, 51, 52, 55, 57, 59, 60, 61, 64, 66, 67, 68, 69, 71, 72, 74, 75, 77, 81, 82, 84, 85, 86, 88, 95, 97, 98, 101, 102, 104, 105, 106, 107, 110, 111, 117, 118, 122, 125, 126, 128, 133, 134, 135, 140, 142, 144, 157 |
|  |  |  |  |
| **Systematic review** | - | 12, 13, 22, 32, 45, 58, 79, 96, 103, 112, 113, 114, 115, 116, 127, 130, 146, 149 | - |
| **Systematic review and meta-analysis** | - | 6, 53, 65, 80, 91, 92, 109, 150, 152 | - |

References

Original Articles:

1. Schuknecht HF. Ablation therapy for the relief of Meniere’s disease. Laryngoscope. 1956;66(7):859–70.

2. Shenoi PM. Ototoxicity of absorbable gelatin sponge. Proc R Soc Med. 1973;66(2):193–6.

3. Beck C, Schmidt CL. 10 years of experience with intratympanally applied streptomycin (gentamycin) in the therapy of Morbus Meniere. Arch Otorhinolaryngol. 1978;221(2):149–52.

4. Longridge NS. The value of sodium chloride crystal application to the round window for Meniere’s disease. J Otolaryngol. 1982;11(4):265–6.

5. Silverstein H. Streptomycin treatment for Meniere’s disease. Ann Otol Rhinol Laryngol Suppl. 1984;112:44–8.

6. Silverstein H, Hyman SM, Feldbaum J, Silverstein D. Use of streptomycin sulfate in the treatment of Meniere’s disease. Otolaryngol Head Neck Surg. 1984;92(2):229–32.

7. Fradis M, Podoshin L, Ben-David J, Reiner B. Treatment of Meniere’s disease by intratympanic injection with lidocaine. Arch Otolaryngol. 1985;111(8):491–3.

8. Szabados E, Nagymajtenyi E, Ribari O, Boda K. Effect of transtympanally applied lidocaine on the cochleovestibular function. Acta Chir Hung. 1986;27(1):3–11.

9. Hunt MA, Miller SW, Nielson HC, Horn KM. Intratympanic injection of sodium arsanilate (atoxyl) solution results in postural changes consistent with changes described for labyrinthectomized rats. Behav Neurosci. 1987;101(3):427–8.

10. Yamazaki T, Hayashi M, Hayashi N, Kozaki H. Intratympanic gentamycin therapy for Meniere’s disease placed by tubal catheter with systemic isosorbide. Arch Otorhinolaryngol. 1988;245(3):170–4.

11. Moller C, Odkvist LM, Thell J, Larsby B, Hyden D. Vestibular and audiologic functions in gentamicin-treated Meniere’s disease. Am J Otol. 1988;9(5):383–91.

12. Proctor LR, el-Kashef Y. The use of streptomycin to induce unilateral ablation of vestibular function in the rat: a preliminary report. Am J Otolaryngol. 1989;10(3):188–97.

13. Laffree JB, Vermeij P, Hulshof JH. The effect of iontophoresis of lignocaine in the treatment of tinnitus. Clin Otolaryngol Allied Sci. 1989;14(5):401–4.

14. Shea JJ. Perfusion of the inner ear with streptomycin. Am J Otol. 1989;10(2):150–5.

15. Schmidt SH, Anniko M, Hellstrom S. Electrophysiological effects of the clinically used local anesthetics lidocaine, lidocaine-prilocaine and phenol on the rat’s inner ear. Eur Arch Otorhinolaryngol. 1990;248(2):87–94.

16. Laitakari K. Intratympanic gentamycin in severe Meniere’s disease. Clin Otolaryngol Allied Sci. 1990;15(6):545–8.

17. Norris CH, Amedee RG, Risey JA, Shea JJ. Selective chemical vestibulectomy. Am J Otol. 1990;11(6):395–400.

18. Yamazaki T, Hayashi M, Komatsuzaki A. Intratympanic gentamicin therapy for Meniere’s disease placed by a tubal catheter with systematic isosorbide. Acta Otolaryngol Suppl. 1991;481:613–6.

19. Magnusson M, Padoan S. Delayed onset of ototoxic effects of gentamicin in treatment of Meniere’s disease. Rationale for extremely low dose therapy. Acta Otolaryngol. 1991;111(4):671–6.

20. Itoh A, Sakata E. Treatment of vestibular disorders. Acta Otolaryngol Suppl. 1991;481:617–23.

21. Kimura RS, Lee KS, Nye CL, Trehey JA. Effects of systemic and lateral semicircular canal administration of aminoglycosides on normal and hydropic inner ears. Acta Otolaryngol. 1991;111(6):1021–30.

22. Shea JJ, Norris CH. Streptomycin perfusion of the labyrinth. Acta Otolaryngol Suppl. 1991;485:123–30.

23. Nedzelski JM, Schessel DA, Bryce GE, Pfleiderer AG. Chemical labyrinthectomy: local application of gentamicin for the treatment of unilateral Meniere’s disease. Am J Otol. 1992;13(1):18–22.

24. Nedzelski JM, Bryce GE, Pfleiderer AG. Treatment of Meniere’s disease with topical gentamicin: a preliminary report. J Otolaryngol. 1992;21(2):95–101.

25. Podoshin L, Fradis M, David Y Ben. Treatment of tinnitus by intratympanic instillation of lignocaine (lidocaine) 2 per cent through ventilation tubes. J Laryngol Otol. 2018;106:603–6.

26. Coles RR, Thompson AC, O’Donoghue GM. Intra-tympanic injections in the treatment of tinnitus. Clin Otolaryngol Allied Sci. 1992;17(3):240–2.

27. Monsell EM, Shelton C. Labyrinthotomy with streptomycin infusion: early results of a multicenter study. The LSI Multicenter Study Group. Am J Otol. 1992;13(5):415–6.

28. Brown JN, Miller JM, Altschuler RA, Nuttall AL. Osmotic pump implant for chronic infusion of drugs into the inner ear. Hear Res. 1993;70(2):167–72.

29. Nedzelski JM, Chiong CM, Fradet G, Schessel DA, Bryce GE, Pfleiderer AG. Intratympanic gentamicin instillation as treatment of unilateral Meniere’s disease: update of an ongoing study. Am J Otol. 1993;14(3):278–82.

30. Davies E, Gladstone HB, Williams H, Hradek G, Shah SB, Schindler RA. A model for long-term intracochlear administration of pharmacologic agents. Am J Otol. 1994;15(6):757–61.

31. Inoue H, Uchi Y, Nogami K, Uemura T. Low-dose intratympanic gentamicin treatment of Meniere’s disease. Eur Arch Otorhinolaryngol. 1994;251 Suppl:S12-4.

32. Pyykko I, Ishizaki H, Kaasinen S, Aalto H. Intratympanic gentamicin in bilateral Meniere’s disease. Otolaryngol Head Neck Surg. 1994;110(2):162–7.

33. Shah SB, Gladstone HB, Williams H, Hradek GT, Schindler RA. An extended study: protective effects of nerve growth factor in neomycin-induced auditory neural degeneration. Am J Otol. 1995;16(3):310–4.

34. Watanabe S, Kato I, Takahashi K, Yoshino K, Takeyama I. Indications and results of gentamycin injection into the middle ear of patients with meniere’s disease. Acta Otolaryngol Suppl. 1995;519:282–5.

35. Toth AA, Parnes LS. Intratympanic gentamicin therapy for Meniere’s disease: preliminary comparison of two regimens. J Otolaryngol. 1995;24(6):340–4.

36. Ito A, Nakashima T, Yanagita N. Effect of Topical Application of Nitroglycerin on Cochlear Blood Flow.

37. Kaasinen S, Pyykko I, Ishizaki H, Aalto H. Effect of intratympanically administered gentamicin on hearing and tinnitus in Meniere’s disease. Acta Otolaryngol Suppl. 1995;520 Pt 1:184–5.

38. Lange G. Transtympanic gentamycin in the treatment of Meniere’s disease. Rev Laryngol Otol Rhinol (Bord). 1995;116(2):151–2.

39. Youssef TF, Poe DS. Intratympanic gentamicin injection for the treatment of Meniere’s disease. Am J Otol. 1998;19(4):435–42.

40. Tribukait A, Bergenius J, Brantberg K. Subjective visual horizontal during follow-up after unilateral vestibular deafferentation with gentamicin. Acta Otolaryngol. 1998;118(4):479–87.

41. Harner SG, Kasperbauer JL, Facer GW, Beatty CW. Transtympanic gentamicin for Meniere’s syndrome. Laryngoscope. 1998;108(10):1446–9.

42. Staecker H, Kopke R, Malgrange B, Lefebvre P, Van de Water TR. NT-3 and/or BDNF therapy prevents loss of auditory neurons following loss of hair cells. Neuroreport. 1996;7(4):889–94.

43. Lalwani AK, Walsh BJ, Reilly PG, Muzyczka N, Mhatre AN. Development of in vivo gene therapy for hearing disorders: introduction of adeno-associated virus into the cochlea of the guinea pig. Gene Ther. 1996;3(7):588–92.

44. Silverstein H, Choo D, Rosenberg SI, Kuhn J, Seidman M, Stein I. Intratympanic steroid treatment of inner ear disease and tinnitus (preliminary report). Ear Nose Throat J. 1996;75(8):48–471.

45. Sakata, Itoh, Itoh. Treatment of Cochlear-Tinnitus with Dexamethasone Infusion into the Tympanic Cavity. Int Tinnitus J. 1996;2:129–35.

46. Laurikainen EA, Johansson RK, Kileny PR. Effects of intratympanically delivered lidocaine on the auditory system in humans. Ear Hear. 1996;17(1):49–54.

47. Weiss MA, Frisancho JC, Roessler BJ, Raphael Y. Viral-mediated gene transfer in the cochlea. Int J Dev Neurosci. 1997;15(4–5):577–83.

48. Rauch SD, Oas JG. Intratympanic gentamicin for treatment of intractable Meniere’s disease: a preliminary report. Laryngoscope. 1997;107(1):49–55.

49. Murofushi T, Halmagyi GM, Yavor RA. Intratympanic gentamicin in Meniere’s disease: results of therapy. Am J Otol. 1997;18(1):52–7.

50. Driscoll CL, Kasperbauer JL, Facer GW, Harner SG, Beatty CW. Low-dose intratympanic gentamicin and the treatment of Meniere’s disease: preliminary results. Laryngoscope. 1997;107(1):83–9.

51. Sala T. Transtympanic gentamicin in the treatment of Meniere’s disease. Auris Nasus Larynx. 1997;24(3):239–46.

52. Sakata, Ito, Itoh. Clinical Experiences of Steroid Targeting Therapy to Inner Ear for Control of Tinnitus. Int Tinnitus J. 1997;3(2):117–21.

53. Corsten M, Marsan J, Schramm D, Robichaud J. Treatment of intractable Meniere’s disease with intratympanic gentamicin: review of the University of Ottawa experience. J Otolaryngol. 1997;26(6):361–4.

54. Lalwani AK, Walsh BJ, Carvalho GJ, Muzyczka N, Mhatre AN. Expression of adeno-associated virus integrated transgene within the mammalian vestibular organs. Am J Otol. 1998;19(3):390–5.

55. Balough BJ, Hoffer ME, Derin W, O’Leary MJ, Brooker CR, Mark G. Kinetics of gentamicin uptake in the inner ear of Chinchilla langier after middle-ear administration in a sustained-release vehicle. Otolaryngol Neck Surg. 1998 Nov 17;119(5):427–31.

56. Arriaga MA, Goldman S. Hearing results of intratympanic steroid treatment of endolymphatic hydrops. Laryngoscope. 1998;108(11 Pt 1):1682–5.

57. Wanamaker HH, Gruenwald L, Damm KJ, Ogata Y, Slepecky N. Dose-related vestibular and cochlear effects of transtympanic gentamicin. Am J Otol. 1998;19(2):170–9.

58. McFeely WJ, Singleton GT, Rodriguez FJ, Antonelli PJ. Intratympanic gentamicin treatment for Meniere’s disease. Otolaryngol Head Neck Surg. 1998;118(5):589–96.

59. Pfleiderer AG. The current role of local intratympanic gentamicin therapy in the management of unilateral Meniere’s disease. Clin Otolaryngol Allied Sci. 1998;23(1):34–41.

60. Shirwany NA, Seidman MD, Tang W. Effect of transtympanic injection of steroids on cochlear blood flow, auditory sensitivity, and histology in the guinea pig. Am J Otol. 1998;19(2):230–5.

61. Silverstein H, Isaacson JE, Olds MJ, Rowan PT, Rosenberg S. Dexamethasone inner ear perfusion for the treatment of Meniere’s disease: a prospective, randomized, double-blind, crossover trial. Am J Otol. 1998;19(2):196–201.

62. Kaasinen S, Pyykko I, Ishizaki H, Aalto H. Intratympanic gentamicin in Meniere’s disease. Acta Otolaryngol. 1998;118(3):294–8.

63. Hicks G. Intratympanic and Round-Window Drug Therapy: Effect on Cochlear Tinnitus. Int Tinnitus J. 1998;4(2):144–7.

64. Staecker H, Gabaizadeh R, Federoff H, Van De Water TR. Brain-derived neurotrophic factor gene therapy prevents spiral ganglion degeneration after hair cell loss. Otolaryngol Head Neck Surg. 1998;119(1):7–13.

65. Wareing M, Mhatre AN, Pettis R, Han JJ, Haut T, P MH, et al. Cationic liposome mediated transgene expression in the guinea pig cochlea.

66. Derby ML, Sena-Esteves M, Breake¢eld XO, Corey DP. Gene transfer into the mammalian inner ear using HSV-1 and vaccinia virus vectors.

67. Yagi M, Magal E, Sheng Z, Ang KA, Raphael Y. Hair cell protection from aminoglycoside ototoxicity by adenovirus-mediated overexpression of glial cell line-derived neurotrophic factor. Hum Gene Ther. 1999;10(5):813–23.

68. Carvalho GJ, Lalwani AK. The effect of cochleostomy and intracochlear infusion on auditory brain stem response threshold in the guinea pig. Am J Otol. 1999;20(1):87–90.

69. Shimogori H, Yamashita H, Watanabe T, Nakamura S. A role of glucocorticoid receptors in the guinea pig vestibular system. Brain Res. 1999;851(1–2):258–60.

70. Silverstein H, Arruda J, Rosenberg SI, Deems D, Hester TO. Direct round window membrane application of gentamicin in the treatment of Meniere’s disease. Otolaryngol Head Neck Surg. 1999;120(5):649–55.

71. Wanamaker HH, Slepecky NB, Cefaratti LK, Ogata Y. Comparison of vestibular and cochlear ototoxicity from transtympanic streptomycin administration. Am J Otol. 1999;20(4):457–64.

72. Silverstein H. Use of a new device, the MicroWick, to deliver medication to the inner ear. Ear Nose Throat J. 1999;78(8):595–600.

73. Quaranta A, Aloisi A, De Benedittis G, Scaringi A. Intratympanic therapy for Meniere’s disease. High-concentration gentamicin with round-window protection. Ann N Y Acad Sci. 1999;884:410–24.

74. Hoffer ME, Balough BJ, Kopke RD, Henderson J, DeCicco M, Wester DC, et al. Morphologic changes in the inner ear of Chinchilla laniger after middle ear administration of gentamicin in a sustained-release vehicle. Otolaryngol Head Neck Surg. 1999;120(5):643–8.

75. Atlas JT, Parnes LS. Intratympanic gentamicin titration therapy for intractable Meniere’s disease. Am J Otol. 1999;20(3):357–63.

76. Eklund S, Pyykko I, Aalto H, Ishizaki H, Vasama JP. Effect of intratympanic gentamicin on hearing and tinnitus in Meniere’s disease. Am J Otol. 1999;20(3):350–6.

77. Minor LB. Intratympanic gentamicin for control of vertigo in Meniere’s disease: vestibular signs that specify completion of therapy. Am J Otol. 1999;20(2):209–19.

78. Pyykko I, Eklund S, Ishizaki H, Aalto H. Postural compensation after intratympanic gentamicin treatment of Meniere’s disease. J Vestib Res. 1999;9(1):19–26.

79. Sennaroglu L, Dini FM, Sennaroglu G, Gursel B, Ozkan S. Transtympanic dexamethasone application in Meniere’s disease: an alternative treatment for intractable vertigo. J Laryngol Otol. 1999;113(3):217–21.

80. Adair RA, Kerr AG. Streptomycin perfusion of the labyrinth in the treatment of Meniere’s disease: a modified technique. Clin Otolaryngol Allied Sci. 1999;24(1):55–7.

81. Suzuki M, Yagi M, Brown JN, Miller AL, Miller JM, Raphael Y. Effect of transgenic GDNF expression on gentamicin-induced cochlear and vestibular toxicity. Gene Ther. 2000;7(12):1046–54.

82. Yagi M, Kanzaki S, Kawamoto K, Shin B, Shah PP, Magal E, et al. Spiral Ganglion Neurons Are Protected from Degeneration by GDNF Gene Therapy. JARO. 2000;01:315–25.

83. Kho ST, Pettis RM, Mhatre AN, Lalwani AK. Cochlear microinjection and its effects upon auditory function in the guinea pig. Eur Arch Otorhinolaryngol. 2000;257(9):469–72.

84. Shimogori H, Yamashita H. Effectiveness and utility of chemical labyrinthectomy with streptomycin using osmotic pump. ORL J Otorhinolaryngol Relat Spec. 2000;62(2):60–2.

85. Shimogori H, Yamashita H. Efficacy of intracochlear administration of betamethasone on peripheral vestibular disorder in the guinea pig. Neurosci Lett. 2000;294(1):21–4.

86. Aarnisalo AA, Pirvola U, Liang XQ, Miller J, Ylikoski J. Apoptosis in auditory brainstem neurons after a severe noise trauma of the organ of Corti: intracochlear GDNF treatment reduces the number of apoptotic cells. ORL J Otorhinolaryngol Relat Spec. 2000;62(6):330–4.

87. Prieskorn DM, Miller JM. Technical report: chronic and acute intracochlear infusion in rodents. Hear Res. 2000;140(1–2):212–5.

88. Kho ST, Pettis RM, Mhatre AN, Lalwani AK. Safety of adeno-associated virus as cochlear gene transfer vector: analysis of distant spread beyond injected cochleae. Mol Ther. 2000;2(4):368–73.

89. Yang GS, Song HT, Keithley EM, Harris JP. Intratympanic immunosuppressives for prevention of immune-mediated sensorineural hearing loss. Am J Otol. 2000;21(4):499–504.

90. Hone SW, Nedzelski J, Chen J. Does intratympanic gentamicin treatment for Meniere’s disease cause complete vestibular ablation?. J Otolaryngol. 2000;29(2):83–7.

91. Kaplan DM, Nedzelski JM, Chen JM, Shipp DB. Intratympanic gentamicin for the treatment of unilateral Meniere’s disease. Laryngoscope. 2000;110(8):1298–305.

92. Adamonis J, Stanton SG, Cashman MZ, Mattan K, Nedzelski JM, Chen JM. Electrocochleography and gentamicin therapy for Meniere’s disease: a preliminary report. Am J Otol. 2000;21(4):534–42.

93. Thomsen J, Charabi S, Tos M. Preliminary results of a new delivery system for gentamicin to the inner ear in patients with Meniere’s disease. Eur Arch Otorhinolaryngol. 2000;257(7):362–5.

94. Charabi S, Thomsen J, Tos M. Round window gentamicin mu-catheter--a new therapeutic tool in Meniere’s disease. Acta Otolaryngol Suppl. 2000;543:108–10.

95. Chandrasekhar SS, Rubinstein RY, Kwartler JA, Gatz M, Connelly PE, Huang E, et al. Dexamethasone pharmacokinetics in the inner ear: comparison of route of administration and use of facilitating agents. Otolaryngol Head Neck Surg. 2000;122(4):521–8.

96. Longridge NS, Mallinson AI. Low-dose intratympanic gentamicin treatment for dizziness in Meniere’s disease. J Otolaryngol. 2000;29(1):35–9.

97. Hirvonen TP, Peltomaa M, Ylikoski J. Intratympanic and systemic dexamethasone for Meniere’s disease. ORL J Otorhinolaryngol Relat Spec. 2000;62(3):117–20.

98. Shulman A, Goldstein B. Intratympanic drug therapy with steroids for tinnitus control: a preliminary report. Int Tinnitus J. 2000;6(1):10–20.

99. DeLucchi E. Transtympanic pilocarpine in tinnitus. Int Tinnitus J. 2000;6(1):37–40.

100. Jero J, Tseng CJ, Mhatre AN, Lalwani AK. A surgical approach appropriate for targeted cochlear gene therapy in the mouse.

101. Kawamoto K, Kanzaki S, Yagi M, Stover T, Prieskorn DM, Dolan DF, et al. Gene-based therapy for inner ear disease. Noise Health. 2001;3(11):37–47.

102. Shimogori H, Yamashita H. Rapid correction of vestibular imbalance by intracochlear administration of ATP in a guinea pig model of unilateral peripheral vestibular disorder. Neurosci Lett. 2001;315(1–2):69–72.

103. Hibi T, Suzuki T, Nakashima T. Perilymphatic concentration of gentamicin administered intratympanically in guinea pigs. Acta Otolaryngol. 2001;121(3):336–41.

104. Jero J, Mhatre AN, Tseng CJ, Stern RE, Coling DE, Goldstein JA, et al. Cochlear Gene Delivery through an Intact Round Window Membrane in Mouse.

105. Schoendorf J, Neugebauer P, Michel O. Continuous intratympanic infusion of gentamicin via a microcatheter in Meniere’s disease. Otolaryngol Head Neck Surg. 2001;124(2):203–7.

106. Hoffer ME, Kopke RD, Weisskopf P, Gottshall K, Allen K, Wester D. Microdose gentamicin administration via the round window microcatheter: results in patients with Meniere’s disease. Ann N Y Acad Sci. 2001;942:46–51.

107. Hoffer ME, Kopke RD, Weisskopf P, Gottshall K, Allen K, Wester D, et al. Use of the round window microcatheter in the treatment of Meniere’s disease. Laryngoscope. 2001;111(11 Pt 1):2046–9.

108. Kopke RD, Hoffer ME, Wester D, O’Leary MJ, Jackson RL. Targeted topical steroid therapy in sudden sensorineural hearing loss. Otol Neurotol. 2001;22(4):475–9.

109. Praetorius M, Limberger A, Müller M, Lehner R, Schick B, Zenner H-P, et al. A Novel Microperfusion System for the Long-Term Local Supply of Drugs to the Inner Ear: Implantation and Function in the Rat Model. Vol. 6, Audiol Neurootol. 2001.

110. Hoffer ME, Allen K, Kopke RD, Weisskopf P, Gottshall K, Wester D. Transtympanic versus sustained-release administration of gentamicin: kinetics, morphology, and function. Laryngoscope. 2001;111(8):1343–57.

111. Quaranta A, Scaringi A, Aloidi A, Quaranta N, Salonna I. Intratympanic therapy for Meniere’s disease: effect of administration of low concentration of gentamicin. Acta Otolaryngol. 2001;121(3):387–92.

112. Harner SG, Driscoll CL, Facer GW, Beatty CW, McDonald TJ. Long-term follow-up of transtympanic gentamicin for Meniere’s syndrome. Otol Neurotol. 2001;22(2):210–4.

113. Soderman AC, Bergenius J, Bagger-Sjoback D, Tjell C, Langius A. Patients’ subjective evaluations of quality of life related to disease-specific symptoms, sense of coherence, and treatment in Meniere’s disease. Otol Neurotol. 2001;22(4):526–33.

114. Chandrasekhar SS. Intratympanic dexamethasone for sudden sensorineural hearing loss: clinical and laboratory evaluation. Otol Neurotol. 2001;22(1):18–23.

115. Sakata H, Kojima Y, Koyama S, Furuya N, Sakata E. Treatment of cochlear tinnitus with transtympanic infusion of 4% lidocaine into the tympanic cavity. Int Tinnitus J. 2001;7(1):46–50.

116. Gianoli GJ, Li JC. Transtympanic steroids for treatment of sudden hearing loss. Otolaryngol Head Neck Surg. 2001;125(3):142–6.

117. Barrs DM, Keyser JS, Stallworth C, McElveen JTJ. Intratympanic steroid injections for intractable Meniere’s disease. Laryngoscope. 2001;111(12):2100–4.

118. Sennaroglu L, Sennaroglu G, Gursel B, Dini FM. Intratympanic dexamethasone, intratympanic gentamicin, and endolymphatic sac surgery for intractable vertigo in Meniere’s disease. Otolaryngol Head Neck Surg. 2001;125(5):537–43.

119. Praetorius M, Knipper M, Schick B, Tan J, Limberger A, Carnicero E, et al. A novel vestibular approach for gene transfer into the inner ear. Audiol Neurootol. 2002;7(6):324–34.

120. Shepherd RK, Xu J. A multichannel scala tympani electrode array incorporating a drug delivery system for chronic intracochlear infusion. Hear Res. 2002;172(1–2):92–8.

121. Laurell G, Teixeira M, Sterkers O, Bagger-Sjo D, «ck B, Eksborg S, et al. Local administration of antioxidants to the inner ear Kinetics and distribution 1.

122. Wang J, Dib M, Lenoir M, Vago P, Eybalin M, Hameg A, et al. Riluzole rescues cochlear sensory cells from acoustic trauma in the guinea-pig. Neuroscience. 2002;111(3):635–48.

123. Himeno C, Komeda M, Izumikawa M, Takemura K, Yagi M, Weiping Y, et al. Intra-cochlear administration of dexamethasone attenuates aminoglycoside ototoxicity in the guinea pig. Hear Res. 2002;167(1–2):61–70.

124. Shinohara T, Bredberg G, Ulfendahl M, Pyykko I, Olivius NP, Kaksonen R, et al. Neurotrophic factor intervention restores auditory function in deafened animals. Proc Natl Acad Sci U S A. 2002;99(3):1657–60.

125. Seidman M. Continuous gentamicin therapy using an IntraEAR microcatheter for Meniere’s disease: a retrospective study. Otolaryngol Head Neck Surg. 2002;126(3):244–56.

126. Lefebvre PP, Staecker H. Steroid Perfusion of the Inner Ear for Sudden Sensorineural Hearing Loss after Failure of Conventional Therapy: A Pilot Study. Acta Otolaryngol. 2002 Feb 1;122(7):698–702.

127. Tonkin J, John Tonkin. New treatment for Meniere’s disease. Aust Fam Physician. 2002;31(8):749–50.

128. David EA, Jackson-Boeters L, Daley T, MacRae DL. Cochlear delivery of fibroblast growth factor 1 and its effects on apoptosis and cell cycling in noise-exposed guinea pig ears. J Otolaryngol. 2002;31(5):304–12.

129. Plontke SKR, Plinkert PK, Plinkert B, Koitschev A, Zenner H-P, Lowenheim H. Transtympanic endoscopy for drug delivery to the inner ear using a new microendoscope. Adv Otorhinolaryngol. 2002;59:149–55.

130. Becvarovski Z, Bojrab DI, Michaelides EM, Kartush JM, Zappia JJ, LaRouere MJ. Round window gentamicin absorption: an in vivo human model. Laryngoscope. 2002;112(9):1610–3.

131. De Waele C, Meguenni R, Freyss G, Zamith F, Bellalimat N, Vidal PP, et al. Intratympanic gentamicin injections for Meniere disease: vestibular hair cell impairment and regeneration. Neurology. 2002;59(9):1442–4.

132. Perez N, Martin E, Zubieta JL, Romero MD, Garcia-Tapia R. Benign paroxysmal positional vertigo in patients with Meniere’s disease treated with intratympanic gentamycin. Laryngoscope. 2002;112(6):1104–9.

133. Carey JP, Hirvonen T, Peng GCY, Della Santina CC, Cremer PD, Haslwanter T, et al. Changes in the angular vestibulo-ocular reflex after a single dose of intratympanic gentamicin for Meniere’s disease. Ann N Y Acad Sci. 2002;956:581–4.

134. Carey JP, Minor LB, Peng GCY, Della Santina CC, Cremer PD, Haslwanter T. Changes in the three-dimensional angular vestibulo-ocular reflex following intratympanic gentamicin for Meniere’s disease. J Assoc Res Otolaryngol. 2002;3(4):430–43.

135. Abou-Halawa AS, Poe DS. Efficacy of increased gentamicin concentration for intratympanic injection therapy in Meniere’s disease. Otol Neurotol. 2002;23(4):493–4.

136. Kaplan DM, Nedzelski JM, Al-Abidi A, Chen JM, Shipp DB. Hearing loss following intratympanic instillation of gentamicin for the treatment of unilateral Meniere’s disease. J Otolaryngol. 2002;31(2):106–11.

137. Yetiser S, Kertmen M. Intratympanic gentamicin in Meniere’s disease: the impact on tinnitus. Int J Audiol. 2002;41(6):363–70.

138. Marzo SJ, Leonetti JP. Intratympanic gentamicin therapy for persistent vertigo after endolymphatic sac surgery. Otolaryngol Head Neck Surg. 2002;126(1):31–3.

139. Cesarani A, Capobianco S, Soi D, Giuliano DA, Alpini D. Intratympanic dexamethasone treatment for control of subjective idiopathic tinnitus: our clinical experience. Int Tinnitus J. 2002;8(2):111–4.

140. Praetorius M, Baker K, Weich CM, Plinkert PK, Staecker H. Hearing preservation after inner ear gene therapy: the effect of vector and surgical approach. ORL J Otorhinolaryngol Relat Spec. 2003;65(4):211–4.

141. Paasche G, Gibson P, Averbeck T, Becker H, Lenarz T, Stover T. Technical report: modification of a cochlear implant electrode for drug delivery to the inner ear. Otol Neurotol. 2003;24(2):222–7.

142. Hakuba N, Matsubara A, Hyodo J, Taniguchi M, Maetani T, Shimizu Y, et al. A MPA / kainate-type glutamate receptor antagonist reduces progressive inner hair cell loss after transient cochlear ischemia. Vol. 979, Brain Research. 2003.

143. Ekborn A, Laurell G, Ehrsson H, Miller J. Intracochlear administration of thiourea protects against cisplatin-induced outer hair cell loss in the guinea pig. Hear Res. 2003;181(1–2):109–15.

144. Wang X, Truong T, Billings PB, Harris JP, Keithley EM. Blockage of immune-mediated inner ear damage by etanercept. Otol Neurotol. 2003;24(1):52–7.

145. Takeda T, Sawada S, Takeda S, Kitano H, Suzuki M, Kakigi A, et al. The effects of V2 antagonist (OPC-31260) on endolymphatic hydrops. Hear Res. 2003;182(1–2):9–18.

146. Light JP, Silverstein H, Jackson LE. Gentamicin perfusion vestibular response and hearing loss. Otol Neurotol. 2003;24(2):294–8.

147. Mills DM, Schmiedt RA. Metabolic presbycusis: differential changes in auditory brainstem and otoacoustic emission responses with chronic furosemide application in the gerbil. J Assoc Res Otolaryngol. 2004;5(1):1–10.

148. Teranishi M, Nakashima T. Effects of trolox, locally applied on round windows, on cisplatin-induced ototoxicity in guinea pigs. Int J Pediatr Otorhinolaryngol. 2003;67(2):133–9.

149. Perez N, Martin E, Garcia-Tapia R. Intratympanic gentamicin for intractable Meniere’s disease. Laryngoscope. 2003;113(3):456–64.

150. Adunka O, Moustaklis E, Weber A, May A, von Ilberg C, Gstoettner W, et al. Labyrinth anesthesia--a forgotten but practical treatment option in Meniere’s disease. ORL J Otorhinolaryngol Relat Spec. 2003;65(2):84–90.

151. Martin E, Perez N. Hearing loss after intratympanic gentamicin therapy for unilateral Meniere’s Disease. Otol Neurotol. 2003;24(5):800–6.

152. Atlas J, Parnes LS. Intratympanic gentamicin for intractable Meniere’s disease: 5-year follow-up. J Otolaryngol. 2003;32(5):288–93.

153. Bottrill I, Wills AD, Mitchell AL. Intratympanic gentamicin for unilateral Meniere’s disease: results of therapy. Clin Otolaryngol Allied Sci. 2003;28(2):133–41.

154. Wu IC, Minor LB. Long-term hearing outcome in patients receiving intratympanic gentamicin for Meniere’s disease. Laryngoscope. 2003;113(5):815–20.

155. Sala T. Meniere’s disease and gentamicin: preliminary results using the minimum effective dose and integrated therapy. Acta Otorhinolaryngol Ital. 2003;23(2):78–87.

156. Perez N, Martin E, Garcia-Tapia R. Results of vestibular autorotation testing at the end of intratympanic gentamicin treatment for Meniere’s disease. Acta Otolaryngol. 2003;123(4):506–14.

157. Hillman TM, Arriaga MA, Chen DA. Intratympanic steroids: do they acutely improve hearing in cases of cochlear hydrops?. Laryngoscope. 2003;113(11):1903–7.

158. Hoffer ME, Wester D, Kopke RD, Weisskopf P, Gottshall K. Transtympanic management of tinnitus. Otolaryngol Clin North Am. 2003;36(2):353–8.

159. Christodoulou P, Doxas PG, Papadakis CE, Maris T, Helidonis ES. Transtympanic iontophoresis of gadopentetate dimeglumine: Preliminary results. Otolaryngol Head Neck Surg. 2003;129:408–21.

160. Kawamoto K, Sha S-H, Minoda R, Izumikawa M, Kuriyama H, Schacht J, et al. Antioxidant Gene Therapy Can Protect Hearing and Hair Cells from Ototoxicity. 2003;

161. Nakaizumi T, Kawamoto K, Minoda R, Raphael Y. Adenovirus-mediated expression of brain-derived neurotrophic factor protects spiral ganglion neurons from ototoxic damage. Audiol Neurootol. 2004;9(3):135–43.

162. Han D, Yu Z, Fan E, Liu C, Liu S, Li Y, et al. Morphology of auditory hair cells in guinea pig cochlea after transgene expression.

163. Sugahara K, Shimogori H, Okuda T, Takemoto T, Yamashita H. Novel method for homogeneous gene transfer to the inner ear. Acta Otolaryngol Suppl. 2004;(553):19–22.

164. Tamura T, Nakagawa T, Iguchi F, Tateya I, Endo T, Kim T-S, et al. Transplantation of neural stem cells into the modiolus of mouse cochleae injured by cisplatin. Acta Otolaryngol Suppl. 2004;(551):65–8.

165. Yamagata T, Miller JM, Ulfendahl M, Olivius NP, Altschuler RA, Pyykko I, et al. Delayed neurotrophic treatment preserves nerve survival and electrophysiological responsiveness in neomycin-deafened guinea pigs. J Neurosci Res. 2004;78(1):75–86.

166. Gillespie LN, Clark GM, Marzella PL. Delayed neurotrophin treatment supports auditory neuron survival in deaf guinea pigs. Neuroreport. 2004;15(7):1121–5.

167. Wolters FLC, Klis SFL, Hamers FPT, de Groot JCMJ, Smoorenburg GF. Perilymphatic application of alpha-melanocyte stimulating hormone ameliorates hearing loss caused by systemic administration of cisplatin. Hear Res. 2004;189(1–2):31–40.

168. Sheppard WM, Wanamaker HH, Pack A, Yamamoto S, Slepecky N, York N. Direct round window application of gentamicin with varying delivery vehicles: A comparison of ototoxicity. 2004;

169. Suryanarayanan R, Cook JA. Long-term results of gentamicin inner ear perfusion in Meniere’s disease. J Laryngol Otol. 2004;118(7):489–95.

170. Lange G, Maurer J, Mann W. Long-term results after interval therapy with intratympanic gentamicin for Meniere’s disease. Laryngoscope. 2004;114(1):102–5.

171. Stokroos R, Kingma H. Selective vestibular ablation by intratympanic gentamicin in patients with unilateral active Meniere’s disease: a prospective, double-blind, placebo-controlled, randomized clinical trial. Acta Otolaryngol. 2004;124(2):172–5.

172. Migliaccio AA, Minor LB, Carey JP. Vergence-mediated modulation of the human angular vestibulo-ocular reflex is unaffected by canal plugging. Exp brain Res. 2008;186(4):581–7.

173. Hillman TA, Chen DA, Arriaga MA. Vestibular nerve section versus intratympanic gentamicin for Meniere’s disease. Laryngoscope. 2004;114(2):216–22.

174. Choe W-T, Chinosornvatana N, Chang KW. Prevention of cisplatin ototoxicity using transtympanic N-acetylcysteine and lactate. Otol Neurotol. 2004;25(6):910–5.

175. Ho HG-M, Lin H-C, Shu M-T, Yang C-C, Tsai H-T. Effectiveness of intratympanic dexamethasone injection in sudden-deafness patients as salvage treatment. Laryngoscope. 2004;114(7):1184–9.

176. Dodson KM, Woodson E, Sismanis A. Intratympanic steroid perfusion for the treatment of Meniere’s disease: a retrospective study. Ear Nose Throat J. 2004;83(6):394–8.

177. Endo T, Nakagawa T, Kita T, Iguchi F, Kim T-S, Tamura T, et al. Novel strategy for treatment of inner ears using a biodegradable gel. Laryngoscope. 2005;115(11):2016–20.

178. Noushi F, Richardson RT, Hardman J, Clark G, O’Leary S. Delivery of neurotrophin-3 to the cochlea using alginate beads. Otol Neurotol. 2005;26(3):528–33.

179. Ito J, Endo T, Nakagawa T, Kita T, Kim T-S, Iguchi F. A new method for drug application to the inner ear. ORL J Otorhinolaryngol Relat Spec. 2005;67(5):272–5.

180. Richardson RT, O’leary S, Wise A, Hardman J, Clark G. A single dose of neurotrophin-3 to the cochlea surrounds spiral ganglion neurons and provides trophic support. Hear Res. 2005;204:37–47.

181. Ruel J, Wang J, Pujol R, Hameg A, Dib M, Puel JL. Neuroprotective effect of riluzole in acute noise-induced hearing loss. Neuroreport. 2005;16(10):1087–90.

182. Hakuba N, Hata R, Morizane I, Feng G, Shimizu Y, Fujita K, et al. Neural stem cells suppress the hearing threshold shift caused by cochlear ischemia. Neuroreport. 2005;16(14):1545–9.

183. Hildebrand MS, Dahl HHM, Hardman J, Coleman B, Shepherd RK, De Silva MG. Survival of partially differentiated mouse embryonic stem cells in the scala media of the guinea pig cochlea. JARO - J Assoc Res Otolaryngol. 2005;6(4):341–54.

184. Shepherd RK, Coco A, Epp SB, Crook JM. Chronic depolarization enhances the trophic effects of brain-derived neurotrophic factor in rescuing auditory neurons following a sensorineural hearing loss. J Comp Neurol. 2005;486(2):145–58.

185. Chen Z, Kujawa SG, McKenna MJ, Fiering JO, Mescher MJ, Borenstein JT, et al. Inner ear drug delivery via a reciprocating perfusion system in the guinea pig. J Control Release. 2005 Jan 26;110(1):1–19.

186. Herr BD, Marzo SJ. Intratympanic steroid perfusion for refractory sudden sensorineural hearing loss. Otolaryngol Head Neck Surg. 2005;132(4):527–31.

187. Plontke S, Lowenheim H, Preyer S, Leins P, Dietz K, Koitschev A, et al. Outcomes research analysis of continuous intratympanic glucocorticoid delivery in patients with acute severe to profound hearing loss: basis for planning randomized controlled trials. Acta Otolaryngol. 2005;125(8):830–9.

188. Arnold W, Senn P, Hennig M, Michaelis C, Deingruber K, Scheler R, et al. Novel Slow-and Fast-Type Drug Release Round-Window Microimplants for Local Drug Application to the Cochlea: An Experimental Study in Guinea Pigs. Audiol Neurootol. 2005;10:53–63.

189. Perez N, Rama-Lopez J. Vestibular function at the end of intratympanic gentamicin treatment of patients with Meniere’s disease. J Vestib Res. 2005;15(1):49–58.

190. Lin FR, Migliaccio AA, Haslwanter T, Minor LB, Carey JP. Angular vestibulo-ocular reflex gains correlate with vertigo control after intratympanic gentamicin treatment for Meniere’s disease. Ann Otol Rhinol Laryngol. 2005;114(10):777–85.

191. Perez N, Boleas MS, Martin E. Distortion product otoacoustic emissions after intratympanic gentamicin therapy for unilateral Meniere’s disease. Audiol Neurootol. 2005;10(2):69–78.

192. Gouveris H, Lange G, Mann WJ. Intratympanic gentamicin treatment after endolymphatic sac surgery. Acta Otolaryngol. 2005;125(11):1180–3.

193. Garduno-Anaya MA, Couthino De Toledo H, Hinojosa-Gonzalez R, Pane-Pianese C, Rios-Castaneda LC. Dexamethasone inner ear perfusion by intratympanic injection in unilateral Meniere’s disease: a two-year prospective, placebo-controlled, double-blind, randomized trial. Otolaryngol Head Neck Surg. 2005;133(2):285–94.

194. El-Hennawi DM, El-Deen MHB, Abou-Halawa AS, Nadeem HS, Ahmed MR. Efficacy of intratympanic methylprednisolone acetate in treatment of drill-induced sensorineural hearing loss in guinea pigs. J Laryngol Otol. 2005;119(1):2–7.

195. Selivanova OA, Gouveris H, Victor A, Amedee RG, Mann W. Intratympanic dexamethasone and hyaluronic acid in patients with low-frequency and Meniere’s-associated sudden sensorineural hearing loss. Otol Neurotol. 2005;26(5):890–5.

196. Battista RA. Intratympanic dexamethasone for profound idiopathic sudden sensorineural hearing loss. Otolaryngol Head Neck Surg. 2005;132(6):902–5.

197. Yilmaz I, Yilmazer C, Erkan AN, Aslan SG, Ozluoglu LN. Intratympanic dexamethasone injection effects on transient-evoked otoacoustic emission. Am J Otolaryngol. 2005;26(2):113–7.

198. Araujo MFS, Oliveira CA, Bahmad FMJ. Intratympanic dexamethasone injections as a treatment for severe, disabling tinnitus: does it work?. Arch Otolaryngol Head Neck Surg. 2005;131(2):113–7.

199. Gouveris H, Selivanova O, Mann W. Intratympanic dexamethasone with hyaluronic acid in the treatment of idiopathic sudden sensorineural hearing loss after failure of intravenous steroid and vasoactive therapy. Eur Arch Oto-Rhino-Laryngology Head Neck. 2005 Jan 27;262(2):131–4.

200. Slattery WH, Fisher LM, Iqbal Z, Friedman RA, Liu N. Intratympanic steroid injection for treatment of idiopathic sudden hearing loss. Otolaryngol Head Neck Surg. 2005;133(2):251–9.

201. Rask-Andersen H, Friberg U, Johansson M, Stjernschantz J. Effects of intratympanic injection of latanoprost in Meniere’s disease: a randomized, placebo-controlled, double-blind, pilot study. Otolaryngol Head Neck Surg. 2005;133(3):441–3.

202. Banerjee A, Parnes LS. Intratympanic corticosteroids for sudden idiopathic sensorineural hearing loss. Otol Neurotol. 2005;26(5):878–81.

203. Lautermann J, Sudhoff H, Junker R. Transtympanic corticoid therapy for acute profound hearing loss. Eur Arch Otorhinolaryngol. 2005;262(7):587–91.

204. Matsuoka AJ, Kondo T, Miyamoto RT, Hashino E. In vivo and in vitro characterization of bone marrow-derived stem cells in the cochlea. Laryngoscope. 2006;116(8):1363–7.

205. Cooper LB, Chan DK, Roediger FC, Shaffer BR, Fraser JF, Musatov S, et al. AAV-mediated delivery of the caspase inhibitor XIAP protects against cisplatin ototoxicity. Otol Neurotol. 2006;27(4):484–90.

206. Hu Z, Ulfendahl M. Cell Replacement Therapy in the Inner Ear. Vol. 15, STEM CELLS AND DEVELOPMENT. 2006.

207. Hahn H, Kammerer B, DiMauro A, Salt AN, Plontke SK. Cochlear microdialysis for quantification of dexamethasone and fluorescein entry into scala tympani during round window administration. Hear Res. 2006 Feb 10;212(1–2):236–44.

208. Sendowski I, Abaamrane L, Raffin F, Cros A, Clarencon D. Therapeutic efficacy of intra-cochlear administration of methylprednisolone after acoustic trauma caused by gunshot noise in guinea pigs. Hear Res. 2006;221(1–2):119–27.

209. Hill SL 3rd, Digges ENB, Silverstein H. Long-term follow-up after gentamicin application via the Silverstein MicroWick in the treatment of Meniere’s disease. Ear Nose Throat J. 2006;85(8):494–8.

210. Flanagan S, Mukherjee P, Tonkin J. Outcomes in the use of intra-tympanic gentamicin in the treatment of Meniere’s disease. J Laryngol Otol. 2006;120(2):98–102.

211. Plontke SK, Zimmermann R, Zenner H-P, Lowenheim H. Technical note on microcatheter implantation for local inner ear drug delivery: surgical technique and safety aspects. Otol Neurotol. 2006;27(7):912–7.

212. Kopke RD, Wassel RA, Mondalek F, Grady B, Chen K, Liu J, et al. Magnetic nanoparticles: inner ear targeted molecule delivery and middle ear implant. Audiol Neurootol. 2006;11(2):123–33.

213. Naci Ozluoglu L, Yilmaz I, Akkuzu B, Haberal A. Buffered papaverine facilitates passage of intratympanic dexamethasone to the inner ear. Acta Otolaryngol. 2006;126(12):1260–5.

214. Liu H-J, Dong M-M, Chi F-L. Dexamethasone Pharmacokinetics in Guinea Pig Inner Ear Perilymph. ORL. 2006;68:93–8.

215. Magnusson AK, Tham R. Reversible and controlled peripheral vestibular loss by continuous infusion of ropivacaine (Narop ® ) into the round window niche of rats. Neurosci Lett. 2006;400:16–20.

216. Mynatt R, Hale SA, Gill RM, Plontke SK, Salt AN. Demonstration of a longitudinal concentration gradient along scala tympani by sequential sampling of perilymph from the cochlear apex. J Assoc Res Otolaryngol. 2006;7(2):182–93.

217. Horii A, Saika T, Uno A, Nishiike S, Mitani K, Nishimura M, et al. Factors relating to the vertigo control and hearing changes following intratympanic gentamicin for intractable Meniere’s disease. Otol Neurotol. 2006;27(6):896–900.

218. Bertino G, Durso D, Manfrin M, Casati L, Mira E. Intratympanic gentamicin in monolateral Meniere’s disease: our experience. Eur Arch Otorhinolaryngol. 2006;263(3):271–5.

219. Smith WK, Sandooram D, Prinsley PR. Intratympanic gentamicin treatment in Meniere’s disease: patients’ experiences and outcomes. J Laryngol Otol. 2006;120(9):730–5.

220. Takai Y, Murofushi T, Ushio M, Iwasaki S. Recovery of subjective visual horizontal after unilateral vestibular deafferentation by intratympanic instillation of gentamicin. J Vestib Res. 2006;16(1–2):69–73.

221. Takumida M, Anniko M. Protective effect of edaravone against the ototoxicity of Pseudomonas aeruginosa exotoxin A. Acta Otolaryngol. 2006;126(1):15–9.

222. Garcia-Berrocal JR, Ibanez A, Rodriguez A, Gonzalez-Garcia JA, Verdaguer JM, Trinidad A, et al. Alternatives to systemic steroid therapy for refractory immune-mediated inner ear disease: A physiopathologic approach. Eur Arch Otorhinolaryngol. 2006;263(11):977–82.

223. Roebuck J, Chang CYJ. Efficacy of steroid injection on idiopathic sudden sensorineural hearing loss. Otolaryngol Head Neck Surg. 2006;135(2):276–9.

224. Choung Y-H, Park K, Shin YR, Cho MJ. Intratympanic dexamethasone injection for refractory sudden sensorineural hearing loss. Laryngoscope. 2006;116(5):747–52.

225. Hargunani CA, Kempton JB, DeGagne JM, Trune DR. Intratympanic injection of dexamethasone: time course of inner ear distribution and conversion to its active form. Otol Neurotol. 2006;27(4):564–9.

226. Xenellis J, Papadimitriou N, Nikolopoulos T, Maragoudakis P, Segas J, Tzagaroulakis A, et al. Intratympanic steroid treatment in idiopathic sudden sensorineural hearing loss: a control study. Otolaryngol Head Neck Surg. 2006;134(6):940–5.

227. Dallan I, Bruschini L, Nacci A, Bruschini P, Traino C, Rognini F, et al. Transtympanic steroids as a salvage therapy in sudden hearing loss: preliminary results. ORL J Otorhinolaryngol Relat Spec. 2006;68(5):247–52.

228. Dallan I, Bruschini L, Nacci A, Fattorp B, Traino AC, Rognini F, et al. Transtympanic steroids in refractory sudden hearing loss. Personal experience. Acta Otorhinolaryngol Ital. 2006;26(1):14–9.

229. Arikan OK, Muluk NB, Budak B, Apan A, Budak G, Koc C. Effects of ropivacaine on transient-evoked otoacoustic emissions: a rabbit model. Eur Arch Otorhinolaryngol. 2006;263(5):421–5.

230. Van Wijk F, Staecker H, Keithley E, Lefebvre PP. Local perfusion of the tumor necrosis factor alpha blocker infliximab to the inner ear improves autoimmune neurosensory hearing loss. Audiol Neurootol. 2006;11(6):357–65.

231. Kasemsuwan L, Jariengprasert C, Chaturapatranont S. Transtympanic gentamicin treatment in Meniere’s disease: a preliminary report. J Med Assoc Thai. 2006;89(7):979–85.

232. Banerjee AS, Johnson IJM. Intratympanic gentamicin for Meniere’s disease: effect on quality of life as assessed by Glasgow benefit inventory. J Laryngol Otol. 2006;120(10):827–31.

233. Kakehata S, Sasaki A, Oji K, Futai K, Ota S, Makinae K, et al. Comparison of intratympanic and intravenous dexamethasone treatment on sudden sensorineural hearing loss with diabetes. Otol Neurotol. 2006;27(5):604–8.

234. Okano T, Nakagawa T, Kita T, Endo T, Ito J. Cell-gene delivery of brain-derived neurotrophic factor to the mouse inner ear. Mol Ther. 2006;14(6):866–71.

235. Ye Q, Tillein J, Hartmann R, Gstoettner W, Kiefer J. Application of a Corticosteroid (Triamcinolon) Protects Inner Ear Function after Surgical Intervention. Ear Hear. 2007 Jun;28(3):361–9.

236. Rejali D, Lee VA, Abrashkin KA, Humayun N, Swiderski DL, Raphael Y. Cochlear implants and ex vivo BDNF gene therapy protect spiral ganglion neurons. Hear Res. 2007;228(1–2):180–7.

237. Venail F, Wang J, Ruel J, Ballana E, Rebillard G, Eybalin M, et al. Coxsackie adenovirus receptor and alpha nu beta3/alpha nu beta5 integrins in adenovirus gene transfer of rat cochlea. Gene Ther. 2007;14(1):30–7.

238. Wenzel GI, Xia A, Funk E, Evans MB, Palmer DJ, Ng P, et al. Helper-dependent adenovirus-mediated gene transfer into the adult mouse cochlea. Otol Neurotol. 2007;28(8):1100–8.

239. Sharif S, Nakagawa T, Ohno T, Matsumoto M, Kita T, Riazuddin S, et al. The potential use of bone marrow stromal cells for cochlear cell therapy. Neuroreport. 2007;18(4):351–4.

240. Liu Y-H, Ke X-M, Qin Y, Gu Z-P, Xiao S-F. Adeno-associated virus-mediated Bcl-xL prevents aminoglycoside-induced hearing loss in mice. Chin Med J (Engl). 2007;120(14):1236–40.

241. Liu Y, Okada T, Nomoto T, Ke X, Kume A, Ozawa K, et al. Promoter effects of adeno-associated viral vector for transgene expression in the cochlea in vivo. Exp Mol Med. 2007;39(2):170–5.

242. Chan DK, Lieberman DM, Musatov S, Goldfein JA, Selesnick SH, Kaplitt MG. Protection against cisplatin-induced ototoxicity by adeno-associated virus-mediated delivery of the X-linked inhibitor of apoptosis protein is not dependent on caspase inhibition. Otol Neurotol. 2007;28(3):417–25.

243. Salt AN, Sirjani DB, Hartsock JJ, Gill RM, Plontke SK. Marker retention in the cochlea following injections through the round window membrane. Hear Res. 2007;232(1–2):78–86.

244. Miller JM, Le Prell CG, Prieskorn DM, Wys NL, Altschuler RA. Delayed neurotrophin treatment following deafness rescues spiral ganglion cells from death and promotes regrowth of auditory nerve peripheral processes: effects of brain-derived neurotrophic factor and fibroblast growth factor. J Neurosci Res. 2007;85(9):1959–69.

245. McGuinness SL, Shepherd RK. Exogenous BDNF rescues rat spiral ganglion neurons in vivo. Otol Neurotol. 2005;26(5):1064–72.

246. Coleman JKM, Littlesunday C, Jackson R, Meyer T. AM-111 protects against permanent hearing loss from impulse noise trauma. Hear Res. 2007;226(1–2):70–8.

247. Shimogori H, Takemoto T, Mikuriya T, Yamashita H. Edaravone protects the vestibular periphery from free radical-induced toxicity in response to perilymphatic application of (+/-)-alpha-amino-3-hydroxy-5-methyl-isoxazole-4-propionic acid. Eur J Pharmacol. 2007;554(2–3):223–8.

248. Maeda Y, Fukushima K, Kawasaki A, Nishizaki K, Smith RJH. Cochlear expression of a dominant-negative GJB2 R75W construct delivered through the round window membrane in mice. 2007;

249. Takeda T, Takeda S, Kakigi A, Okada T, Nishioka R, Taguchi D. A comparison of dehydration effects of V 2 -antagonist (OPC-31260) on the inner ear between systemic and round window applications. 2006;

250. Ge X, Jackson RL, Liu J, Harper EA, Hoffer ME, Wassel RA, et al. Distribution of PLGA nanoparticles in chinchilla cochleae. Otolaryngol Head Neck Surg. 2007;137(4):619–23.

251. Roehm P, Hoffer M, Balaban CD. Gentamicin uptake in the chinchilla inner ear. Hear Res. 2007;230(1–2):43–52.

252. Colletti V, Carner M, Colletti L. Auditory results after vestibular nerve section and intratympanic gentamicin for Meniere’s disease. Otol Neurotol. 2007;28(2):145–51.

253. Chung W-H, Chung KW, Kim JH, Cho Y-S, Hong SH. Effects of a single intratympanic gentamicin injection on Meniere’s disease. Acta Otolaryngol Suppl. 2007;(558):61–6.

254. Lyford-Pike S, Vogelheim C, Chu E, Della Santina CC, Carey JP. Gentamicin is primarily localized in vestibular type I hair cells after intratympanic administration. J Assoc Res Otolaryngol. 2007;8(4):497–508.

255. De Beer L, Stokroos R, Kingma H. Intratympanic gentamicin therapy for intractable Meniere’s disease. Acta Otolaryngol. 2007;127(6):605–12.

256. De Stefano A, Dispenza F, De Donato G, Caruso A, Taibah A, Sanna M. Intratympanic gentamicin: a 1-day protocol treatment for unilateral Meniere’s disease. Am J Otolaryngol. 2007;28(5):289–93.

257. Daldal A, Odabasi O, Serbetcioglu B. The protective effect of intratympanic dexamethasone on cisplatin-induced ototoxicity in guinea pigs. Otolaryngol Head Neck Surg. 2007;137(5):747–52.

258. Van Wijck F, Staecker H, Lefebvre PP. Topical steroid therapy using the Silverstein Microwick in sudden sensorineural hearing loss after failure of conventional treatment. Acta Otolaryngol. 2007;127(10):1012–7.

259. Bodmer D, Morong S, Stewart C, Alexander A, Chen JM, Nedzelski JM. Long-term vertigo control in patients after intratympanic gentamicin instillation for Meniere’s disease. Otol Neurotol. 2007;28(8):1140–4.

260. Kasemsuwan L, Jariengprasert C, Ruencharopen S, Orathai P. Low dose transtympanic gentamicin treatment for intractable Meniere’s disease: a prospective study. J Med Assoc Thai. 2007;90(2):327–34.

261. Helling K, Schonfeld U, Clarke AH. Treatment of Meniere’s disease by low-dosage intratympanic gentamicin application: effect on otolith function. Laryngoscope. 2007;117(12):2244–50.

262. Suckfuell M, Canis M, Strieth S, Scherer H, Haisch A. Intratympanic treatment of acute acoustic trauma with a cell-permeable JNK ligand: a prospective randomized phase I/II study. Acta Otolaryngol. 2007;127(9):938–42.

263. Haynes DS, O’Malley M, Cohen S, Watford K, Labadie RF. Intratympanic dexamethasone for sudden sensorineural hearing loss after failure of systemic therapy. Laryngoscope. 2007;117(1):3–15.

264. Kilic R, Safak MA, Oguz H, Kargin S, Demirci M, Samim E, et al. Intratympanic methylprednisolone for sudden sensorineural hearing loss. Otol Neurotol. 2007;28(3):312–6.

265. Fitzgerald DC, McGuire JF. Intratympanic steroids for idiopathic sudden sensorineural hearing loss. Ann Otol Rhinol Laryngol. 2007;116(4):253–6.

266. Plaza G, Herraiz C. Intratympanic steroids for treatment of sudden hearing loss after failure of intravenous therapy. Otolaryngol Head Neck Surg. 2007;137(1):74–8.

267. Bird PA, Begg EJ, Zhang M, Keast AT, Murray DP, Balkany TJ. Intratympanic versus intravenous delivery of methylprednisolone to cochlear perilymph. Otol Neurotol. 2007;28(8):1124–30.

268. Shepherd RK, Coco A, Epp SB. Neurotrophins and electrical stimulation for protection and repair of spiral ganglion neurons following sensorineural hearing loss. Hear Res. 2008;242(1–2):100–9.

269. Backhouse S, Coleman B, Shepherd R. Surgical access to the mammalian cochlea for cell-based therapies. Exp Neurol. 2008;214(2):193–200.

270. Ballana E, Wang J, Venail F, Estivill X, Puel JL, Arbonès ML, et al. Efficient and specific transduction of cochlear supporting cells by adeno-associated virus serotype 5. Neurosci Lett. 2008;

271. Konishi M, Kawamoto K, Izumikawa M, Kuriyama H, Yamashita T. Gene transfer into guinea pig cochlea using adeno-associated virus vectors. J Gene Med. 2008;10(6):610–8.

272. Bogaerts S, Douglas S, Corlette T, Pau H, Saunders D, McKay S, et al. Microsurgical access for cell injection into the mammalian cochlea. J Neurosci Methods. 2008;168(1):156–63.

273. Pietola L, Aarnisalo AA, Joensuu J, Pellinen R, Wahlfors J, Jero J. HOX-GFP and WOX-GFP lentivirus vectors for inner ear gene transfer. Acta Otolaryngol. 2008;128(6):613–20.

274. Tan BTG, Foong KH, Lee MMG, Ruan R. Polyethylenimine-mediated cochlear gene transfer in guinea pigs. Arch Otolaryngol - Head Neck Surg. 2008;134(8):884–91.

275. Vivero RJ, Joseph DE, Angeli S, He J, Chen S, Eshraghi AA, et al. Dexamethasone base conserves hearing from electrode trauma-induced hearing loss. Laryngoscope. 2008;118(11):2028–35.

276. Plontke SK, Biegner T, Kammerer B, Delabar U, Salt AN. Dexamethasone concentration gradients along scala tympani after application to the round window membrane. Otol Neurotol. 2008;29(3):401–6.

277. Song B, Li Y, Han D. Effects of delayed brain-derived neurotrophic factor application on cochlear pathology and auditory physiology in rats. Chin Med J (Engl). 2008;121(13):1189–96.

278. Reyes JH, O’Shea KS, Wys NL, Velkey JM, Prieskorn DM, Wesolowski K, et al. Glutamatergic neuronal differentiation of mouse embryonic stem cells after transient expression of neurogenin 1 and treatment with BDNF and GDNF: in vitro and in vivo studies. J Neurosci. 2008;28(48):12622–31.

279. Agterberg MJH, Versnel H, de Groot JCMJ, Smoorenburg GF, Albers FWJ, Klis SFL. Morphological changes in spiral ganglion cells after intracochlear application of brain-derived neurotrophic factor in deafened guinea pigs. Hear Res. 2008;244(1–2):25–34.

280. James DP, Eastwood H, Richardson RT, O’Leary SJ. Effects of round window dexamethasone on residual hearing in a Guinea pig model of cochlear implantation. Audiol Neurootol. 2008;13(2):86–96.

281. Yang J, Wu H, Zhang P, Hou DM, Chen J, Zhang SG. The pharmacokinetic profiles of dexamethasone and methylprednisolone concentration in perilymph and plasma following systemic and local administration. Acta Otolaryngol. 2008;128(5):496–504.

282. Guyot J-P, Maire R, Delaspre O. Intratympanic application of an antiviral agent for the treatment of Meniere’s disease. ORL J Otorhinolaryngol Relat Spec. 2008;70(1):21–7.

283. Mikulec AA, Hartsock JJ, Salt AN. Permeability of the round window membrane is influenced by the composition of applied drug solutions and by common surgical procedures. Otol Neurotol. 2008;29(7):1020–6.

284. Zou J, Saulnier P, Perrier T, Zhang Y, Manninen T, Toppila E, et al. Distribution of lipid nanocapsules in different cochlear cell populations after round window membrane permeation. J Biomed Mater Res B Appl Biomater. 2008;87(1):10–8.

285. Paulson DP, Abuzeid W, Jiang H, Oe T, O’Malley BW, Li D. A novel controlled local drug delivery system for inner ear disease. Laryngoscope. 2008;118(4):706–11.

286. Zou J, Asukas J, Inha T, Toppila E, Kellomaki M, Pyykko I. Biocompatibility of different biopolymers after being implanted into the rat cochlea. Otol Neurotol. 2008;29(5):714–9.

287. Guitton MJ, Dudai Y. Blockade of cochlear NMDA receptors prevents long-term tinnitus during a brief consolidation window after acoustic trauma. Neural Plast. 2007;2007:80904.

288. Enrique-Gonzaleza A, Sanchez-Ferrandiz N, Perez-Fernandez N. Disability in patients with Meniere’s disease following the use of two different treatment modalities: betahistine and intratympanic gentamicin. Rev Laryngol Otol Rhinol (Bord). 2008;129(4–5):249–54.

289. Hill GW, Morest DK, Parham K. Cisplatin-induced ototoxicity: effect of intratympanic dexamethasone injections. Otol Neurotol. 2008;29(7):1005–11.

290. Battaglia A, Burchette R, Cueva R. Combination therapy (intratympanic dexamethasone + high-dose prednisone taper) for the treatment of idiopathic sudden sensorineural hearing loss. Otol Neurotol. 2008;29(4):453–60.

291. Boleas-Aguirre MS, Lin FR, Della Santina CC, Minor LB, Carey JP. Longitudinal results with intratympanic dexamethasone in the treatment of Meniere’s disease. Otol Neurotol. 2008;29(1):33–8.

292. Lee H-S, Kim JM, Kim Y-J, Chung DH, Seo BS, Kim SH. Results of intratympanic dexamethasone injection as salvage treatment in idiopathic sudden hearing loss. J Otolaryngol Head Neck Surg. 2008;37(2):263–8.

293. Ahn JH, Han MW, Kim JH, Chung JW, Yoon TH. Therapeutic effectiveness over time of intratympanic dexamethasone as salvage treatment of sudden deafness. Acta Otolaryngol. 2008;128(2):128–31.

294. Alatas N. Use of intratympanic dexamethasone for the therapy of low frequency hearing loss. Eur Arch Otorhinolaryngol. 2009;266(8):1205–12.

295. Ahn KS, Jeon S-J, Jung J-Y, Kim YS, Kang JH, Shin S, et al. Isolation of embryonic stem cells from enhanced green fluorescent protein-transgenic mouse and their survival in the cochlea after allotransplantation. Cytotherapy. 2008;10(7):759–69.

296. Paasche G, Tasche C, Stover T, Lesinski-Schiedat A, Lenarz T. The long-term effects of modified electrode surfaces and intracochlear corticosteroids on postoperative impedances in cochlear implant patients. Otol Neurotol. 2009;30(5):592–8.

297. Scheper V, Paasche G, Miller JM, Warnecke A, Berkingali N, Lenarz T, et al. Effects of delayed treatment with combined GDNF and continuous electrical stimulation on spiral ganglion cell survival in deafened guinea pigs. J Neurosci Res. 2009;87(6):1389–99.

298. Shibata SB, Di Pasquale G, Cortez SR, Chiorini JA, Raphael Y. Gene transfer using bovine adeno-associated virus in the guinea pig cochlea. Gene Ther. 2009;16(8):990–7.

299. Ogita H, Nakagawa T, Lee KY, Inaoka T, Okano T, Kikkawa YS, et al. Surgical invasiveness of cell transplantation into the guinea pig cochlear modiolus. ORL J Otorhinolaryngol Relat Spec. 2009;71(1):32–9.

300. Praetorius M, Brough DE, Hsu C, Plinkert PK, Pfannenstiel SC, Staecker H. Adenoviral vectors for improved gene delivery to the inner ear. Hear Res. 2009;248(1–2):31–8.

301. Agterberg MJH, Versnel H, van Dijk LM, de Groot JCMJ, Klis SFL. Enhanced survival of spiral ganglion cells after cessation of treatment with brain-derived neurotrophic factor in deafened guinea pigs. J Assoc Res Otolaryngol. 2009;10(3):355–67.

302. Okuda T, Sugahara K, Shimogori H, Yamashita H. Inner ear changes with intracochlear gentamicin administration in Guinea pigs. Laryngoscope. 2004;114(4):694–7.

303. Radeloff A, Smolders JWT. Brain-derived neurotrophic factor treatment does not improve functional recovery after hair cell regeneration in the pigeon. Acta Otolaryngol. 2006;126(5):452–9.

304. Hu Z, Ulfendahl M, Prieskorn DM, Olivius P, Miller JM. Functional evaluation of a cell replacement therapy in the inner ear. Otol Neurotol. 2009;30(4):551–8.

305. Sewell WF, Borenstein JT, Chen Z, Fiering J, Handzel O, Holmboe M, et al. Development of a microfluidics-based intracochlear drug delivery device. Audiol Neurootol. 2009;14(6):411–22.

306. Fransson A, Jarlebark LE, Ulfendahl M. In vivo infusion of UTP and uridine to the deafened guinea pig inner ear: effects on response thresholds and neural survival. J Neurosci Res. 2009;87(7):1712–7.

307. Fiering J, Mescher MJ, Leary Swan EE, Holmboe ME, Murphy BA, Chen Z, et al. Local drug delivery with a self-contained, programmable, microfluidic system. Biomed Microdevices. 2009;11(3):571–8.

308. Scheper V, Wolf M, Scholl M, Kadlecova Z, Perrier T, Klok H-A, et al. Potential novel drug carriers for inner ear treatment: hyperbranched polylysine and lipid nanocapsules. Nanomedicine (Lond). 2009;4(6):623–35.

309. Maini S, Lisnichuk H, Eastwood H, Pinder D, James D, Richardson RT, et al. Targeted therapy of the inner ear. Audiol Neurootol. 2009;14(6):402–10.

310. Hori R, Nakagawa T, Sugimoto Y, Sakamoto T, Yamamoto N, Hamaguchi K, et al. Prostaglandin E receptor subtype EP4 agonist protects cochleae against noise-induced trauma. Neuroscience. 2009;160(4):813–9.

311. Plontke SK, Lowenheim H, Mertens J, Engel C, Meisner C, Weidner A, et al. Randomized, double blind, placebo controlled trial on the safety and efficacy of continuous intratympanic dexamethasone delivered via a round window catheter for severe to profound sudden idiopathic sensorineural hearing loss after failure of systemic the. Laryngoscope. 2009;119(2):359–69.

312. Song B-N, Li Y-X, Han D-M. Delayed electrical stimulation and BDNF application following induced deafness in rats. Acta Otolaryngol. 2009;129(2):142–54.

313. Horiike O, Shimogori H, Yamashita H. Effect of edaravone on streptomycin-induced vestibulotoxicity in the Guinea pig. Laryngoscope. 2004;114(9):1630–2.

314. Iwai K, Nakagawa T, Endo T, Matsuoka Y, Kita T, Kim T-S, et al. Cochlear protection by local insulin-like growth factor-1 application using biodegradable hydrogel. Laryngoscope. 2006;116(4):529–33.

315. Morawski K, Telischi FF, Bohorquez J, Niemczyk K. Preventing hearing damage using topical dexamethasone during reversible cochlear ischemia: an animal model. Otol Neurotol. 2009;30(6):851–7.

316. Hiraumi H, Nakagawa T, Ito J. Efficiency of a transtympanic approach to the round window membrane using a microendoscope. Eur Arch Otorhinolaryngol. 2009;266(3):367–71.

317. Mikulec AA, Plontke SK, Hartsock JJ, Salt AN. Entry of substances into perilymph through the bone of the otic capsule after intratympanic applications in guinea pigs: implications for local drug delivery in humans. Otol Neurotol. 2009;30(2):131–8.

318. Wang X, Dellamary L, Fernandez R, Harrop A, Keithley EM, Harris JP, et al. Dose-dependent sustained release of dexamethasone in inner ear cochlear fluids using a novel local delivery approach. Audiol Neurootol. 2009;14(6):393–401.

319. Kyrodimos E, Aidonis I, Sismanis A. Hearing results following intratympanic gentamicin perfusion for Meniere’s disease. J Laryngol Otol. 2009;123(4):379–82.

320. Nguyen KD, Minor LB, Della Santina CC, Carey JP. Vestibular function and vertigo control after intratympanic gentamicin for Meniere’s disease. Audiol Neurootol. 2009;14(6):361–72.

321. Ahn JH, Yoo MH, Yoon TH, Chung JW. Can intratympanic dexamethasone added to systemic steroids improve hearing outcome in patients with sudden deafness?. Laryngoscope. 2008;118(2):279–82.

322. Han C-S, Park J-R, Boo S-H, Jo J-M, Park K-W, Lee W-Y, et al. Clinical efficacy of initial intratympanic steroid treatment on sudden sensorineural hearing loss with diabetes. Otolaryngol Head Neck Surg. 2009;141(5):572–8.

323. Hong SM, Park CH, Lee JH. Hearing outcomes of daily intratympanic dexamethasone alone as a primary treatment modality for ISSHL. Otolaryngol Head Neck Surg. 2009;141(5):579–83.

324. Fransson A, Maruyama J, Miller JM, Ulfendahl M. Post-treatment effects of local GDNF administration to the inner ears of deafened guinea pigs. J Neurotrauma. 2010;27(9):1745–51.

325. Topak M, Sahin-Yilmaz A, Ozdoganoglu T, Yilmaz HB, Ozbay M, Kulekci M. Intratympanic methylprednisolone injections for subjective tinnitus. J Laryngol Otol. 2009;123(11):1221–5.

326. She W, Dai Y, Du X, Chen F, Ding X, Cui X. Treatment of subjective tinnitus: a comparative clinical study of intratympanic steroid injection vs. oral carbamazepine. Med Sci Monit. 2009;15(6):PI35-9.

327. Suryanarayanan R, Srinivasan VR, O’Sullivan G. Transtympanic gentamicin treatment using Silverstein MicroWick in Meniere’s disease patients: long term outcome. J Laryngol Otol. 2009;123(1):45–9.

328. Silverstein H, Farrugia M, Van Ess M. Dexamethasone inner ear perfusion for subclinical endolymphatic hydrops. Ear Nose Throat J. 2009;88(2):778–85.

329. Hsieh L-C, Lin H-C, Tsai H-T, Ko Y-C, Shu M-T, Lin L-H. High-dose intratympanic gentamicin instillations for treatment of Meniere’s disease: long-term results. Acta Otolaryngol. 2009;129(12):1420–4.

330. Pfannenstiel SC, Praetorius M, Plinkert PK, Brough DE, Staecker H. Bcl-2 gene therapy prevents aminoglycoside-induced degeneration of auditory and vestibular hair cells. Audiol Neurootol. 2009;14(4):254–66.

331. Zou J, Zhang W, Poe D, Qin J, Fornara A, Zhang Y, et al. MRI manifestation of novel superparamagnetic iron oxide nanoparticles in the rat inner ear. Nanomedicine (Lond). 2010;5(5):739–54.

332. Zou J, Sood R, Ranjan S, Poe D, Ramadan UA, Kinnunen PK, et al. Manufacturing and in vivo inner ear visualization of MRI traceable liposome nanoparticles encapsulating gadolinium. J Nanobiotechnology. 2010;8:32.

333. Meen E, Blakley B, Quddusi T. Does intracochlear brain-derived nerve growth factor improve auditory brainstem click thresholds in sensorineural hearing loss?. J Otolaryngol Head Neck Surg. 2010;39(3):232–5.

334. Lei L, Han D. Efficient transduction of spiral ganglion cells using adenovirus type 5 vector in the rat. Acta Otolaryngol. 2010;130(7):810–4.

335. Van De Water TR, Abi Hachem RN, Dinh CT, Bas E, Haake SM, Hoosien G, et al. Conservation of hearing and protection of auditory hair cells against trauma-induced losses by local dexamethasone therapy: molecular and genetic mechanisms. Cochlear Implants Int. 2010;11 Suppl 1:42–55.

336. Wise AK, Hume CR, Flynn BO, Jeelall YS, Suhr CL, Sgro BE, et al. Effects of Localized Neurotrophin Gene Expression on Spiral Ganglion Neuron Resprouting in the Deafened Cochlea. Mol Ther. 2010;18(6):1111–22.

337. Zhang Y, Zhang W, Johnston AH, Newman TA, Pyykko I, Zou J. Improving the visualization of fluorescently tagged nanoparticles and fluorophore-labeled molecular probes by treatment with CuSO(4) to quench autofluorescence in the rat inner ear. Hear Res. 2010;269(1–2):1–11.

338. She W, Dai Y, Du X, Yu C, Chen F, Wang J, et al. Hearing evaluation of intratympanic methylprednisolone perfusion for refractory sudden sensorineural hearing loss. Otolaryngol Head Neck Surg. 2010;142(2):266–71.

339. Wenzel GI, Warnecke A, Stover T, Lenarz T. Effects of extracochlear gacyclidine perfusion on tinnitus in humans: a case series. Eur Arch Otorhinolaryngol. 2010;267(5):691–9.

340. Xu L, Heldrich J, Wang H, Yamashita T, Miyamoto S, Li A, et al. A controlled and sustained local gentamicin delivery system for inner ear applications. Otol Neurotol. 2010;31(7):1115–21.

341. Saber A, Strand SP, Ulfendahl M. Use of the biodegradable polymer chitosan as a vehicle for applying drugs to the inner ear. Eur J Pharm Sci. 2010;39:110–5.

342. Horie RT, Sakamoto T, Nakagawa T, Tabata Y, Okamura N, Tomiyama N, et al. Sustained delivery of lidocaine into the cochlea using poly lactic/glycolic acid microparticles. Laryngoscope. 2010;120(2):377–83.

343. Nakagawa T, Sakamoto T, Hiraumi H, Kikkawa YS, Yamamoto N, Hamaguchi K, et al. Topical insulin-like growth factor 1 treatment using gelatin hydrogels for glucocorticoid-resistant sudden sensorineural hearing loss: a prospective clinical trial. BMC Med. 2010;8:76.

344. Zou J, Zhang W, Poe D, Zhang Y, Ramadan UA, Pyykko I. Differential passage of gadolinium through the mouse inner ear barriers evaluated with 4.7T MRI. Hear Res. 2010;259(1–2):36–43.

345. Zou J, Ramadan UA, Pyykko I. Gadolinium uptake in the rat inner ear perilymph evaluated with 4.7 T MRI: a comparison between transtympanic injection and gelatin sponge-based diffusion through the round window membrane. Otol Neurotol. 2010;31(4):637–41.

346. Katzenell U, Gordon M, Page M. Intratympanic gentamicin injections for the treatment of Meniere’s disease. Otolaryngol Head Neck Surg. 2010;143(5 Suppl 3):S24-9.

347. Lee JS, Kang SU, Hwang HS, Pyun JH, Choung YH, Kim CH. Epicatechin protects the auditory organ by attenuating cisplatin-induced ototoxicity through inhibition of ERK. Toxicol Lett. 2010;199:308–16.

348. Chen Y, Wen L, Hu P, Qiu J, Lu L, Qiao L. Endoscopic intratympanic methylprednisolone injection for treatment of refractory sudden sensorineural hearing loss and one case in pregnancy. J Otolaryngol Head Neck Surg. 2010;39(6):640–5.

349. Zhai F, Liu J-P, Dai C-F, Wang Q, Steyger PS. Evidence-based modification of intratympanic gentamicin injections in patients with intractable vertigo. Otol Neurotol. 2010;31(4):642–8.

350. Hunchaisri N, Chantapant S, Srinangyam N. Intratympanic dexamethasone for refractory sudden sensorineural hearing loss. J Med Assoc Thai. 2010;93(12):1406–14.

351. Raymundo IT, Bahmad FJ, Barros Filho J, Pinheiro TG, Maia NA, Oliveira CA. Intratympanic methylprednisolone as rescue therapy in sudden sensorineural hearing loss. Braz J Otorhinolaryngol. 2010;76(4):499–509.

352. Dallan I, De Vito A, Fattori B, Casani A Pietro, Panicucci E, Berrettini S, et al. Intratympanic methylprednisolone in refractory sudden hearing loss: a 27-patient case series with univariate and multivariate analysis. Otol Neurotol. 2010;31(1):25–30.

353. Filipo R, Covelli E, Balsamo G, Attanasio G. Intratympanic prednisolone therapy for sudden sensorineural hearing loss: A new protocol. Acta Otolaryngol. 2010;130(11):1209–13.

354. Worsoe L, Brandt CT, Lund SP, Ostergaard C, Thomsen J, Caye-Thomasen P. Intratympanic steroid prevents long-term spiral ganglion neuron loss in experimental meningitis. Otol Neurotol. 2010;31(3):394–403.

355. Kara E, Cetik F, Tarkan O, Surmelioglu O. Modified intratympanic treatment for idiopathic sudden sensorineural hearing loss. Eur Arch Otorhinolaryngol. 2010;267(5):701–7.

356. Herraiz C, Plaza G, Aparicio JM, Gallego I, Marcos S, Ruiz C. Transtympanic steroids for Meniere’s disease. Otol Neurotol. 2010;31(1):162–7.

357. Sharma K, Goswami SC, Baruah DK. Use of intratympanic steroid as a primary treatment for sudden sensorineural hearing loss. J Indian Med Assoc. 2010;108(3):148–55.

358. Mukherjea D, Jajoo S, Kaur T, Sheehan KE, Ramkumar V, Rybak LP. Transtympanic administration of short interfering (si)RNA for the NOX3 isoform of NADPH oxidase protects against cisplatin-induced hearing loss in the rat. Antioxid Redox Signal. 2010;13(5):589–98.

359. Saliba I, El Fata F, Ouelette V, Robitaille Y. Are intratympanic injections of N-acetylcysteine and methylprednisolone protective against Cisplatin-induced ototoxicity?. J Otolaryngol Head Neck Surg. 2010;39(3):236–43.

360. Suzuki H, Teranishi M, Naganawa S, Nakata S, Sone M, Nakashima T. Contrast-enhanced MRI of the inner ear after intratympanic injection of meglumine gadopentetate or gadodiamide hydrate. Acta Otolaryngol. 2011;131(2):130–5.

361. Nader M-E, Theoret Y, Saliba I. The role of intratympanic lactate injection in the prevention of cisplatin-induced ototoxicity. Laryngoscope. 2010;120(6):1208–13.

362. Silverstein H, Wazen J, Van Ess MJ, Daugherty J, Alameda YA. Intratympanic gentamicin treatment of patients with Meniere’s disease with normal hearing. Otolaryngol Head Neck Surg. 2010;142(4):570–5.

363. Robey AB, Morrow T, Moore GF. Systemic side effects of transtympanic steroids. Laryngoscope. 2010;120(SUPPL. 4):111.

364. Zhang Y, Zhang W, Johnston AH, Newman TA, Pyykk I, Zou J. Comparison of the distribution pattern of PEG-b-PCL polymersomes delivered into the rat inner ear via different methods. Acta Otolaryngol. 2011;131(12):1249–56.

365. Landry TG, Wise AK, Fallon JB, Shepherd RK. Spiral ganglion neuron survival and function in the deafened cochlea following chronic neurotrophic treatment. Hear Res. 2011;282(1–2):303–13.

366. Zhang L, Jiang H, Hu Z. Concentration-Dependent Effect of Nerve Growth Factor on Cell Fate Determination of Neural Progenitors.

367. Kilpatrick LA, Li Q, Yang J, Goddard JC, Fekete DM, Lang H. Adeno-associated virus-mediated gene delivery into the scala media of the normal and deafened adult mouse ear. Gene Ther. 2011;18(6):569–78.

368. Thaler M, Roy S, Fornara A, Bitsche M, Qin J, Muhammed M, et al. Visualization and analysis of superparamagnetic iron oxide nanoparticles in the inner ear by light microscopy and energy filtered TEM. Nanomedicine. 2011;7(3):360–9.

369. Wise AK, Tu T, Atkinson PJ, Flynn BO, Sgro BE, Hume C, et al. The effect of deafness duration on neurotrophin gene therapy for spiral ganglion neuron protection. Hear Res. 2011;278(1–2):69–76.

370. Wu J, Liu B, Fan J, Zhu Q, Wu J. Study of protective effect on rat cochlear spiral ganglion after blast exposure by adenovirus-mediated human β-nerve growth factor gene. Am J Otolaryngol Neck Med Surg. 2011;32:8–12.

371. Wise AK, Fallon JB, Neil AJ, Pettingill LN, Geaney MS, Skinner SJ, et al. Combining cell-based therapies and neural prostheses to promote neural survival. Neurotherapeutics. 2011;8(4):774–87.

372. Ibrahim HN, Bossard D, Jolly C, Truy E. Radiologic study of a disposable drug delivery intracochlear catheter. Otol Neurotol. 2011;32(2):217–22.

373. Abaamrane L, Raffin F, Schmerber S, Sendowski I. Intracochlear perfusion of leupeptin and z-VAD-FMK: influence of antiapoptotic agents on gunshot-induced hearing loss. Eur Arch Otorhinolaryngol. 2011;268(7):987–93.

374. Pararas EEL, Chen Z, Fiering J, Mescher MJ, Kim ES, McKenna MJ, et al. Kinetics of reciprocating drug delivery to the inner ear. J Control Release. 2011;152(2):270–7.

375. Zhai S-Q, Guo W, Hu Y-Y, Yu N, Chen Q, Wang J-Z, et al. Protective effects of brain-derived neurotrophic factor on the noise-damaged cochlear spiral ganglion. J Laryngol Otol. 2011;125(5):449–54.

376. Zou J, Yoshida T, Ramadan UA, Pyykko I. Dynamic enhancement of the rat inner ear after ultra-small-volume administration of Gd-DOTA to the medial wall of the middle ear cavity. ORL J Otorhinolaryngol Relat Spec. 2011;73(5):275–81.

377. Omotehara Y, Hakuba N, Hato N, Okada M, Gyo K. Protection against ischemic cochlear damage by intratympanic administration of AM-111. Otol Neurotol. 2011;32(9):1422–7.

378. Borden RC, Saunders JE, Berryhill WE, Krempl GA, Thompson DM, Queimado L. Hyaluronic Acid Hydrogel Sustains the Delivery of Dexamethasone across the Round Window Membrane. Audiol Neurotol. 2011;16:1–11.

379. King EB, Salt AN, Eastwood HT, O’Leary SJ. Direct entry of gadolinium into the vestibule following intratympanic applications in Guinea pigs and the influence of cochlear implantation. J Assoc Res Otolaryngol. 2011;12(6):741–51.

380. Zhang Y, Zhang W, Löbler M, Schmitz K-P, Saulnier P, Perrier T, et al. Inner ear biocompatibility of lipid nanocapsules after round window membrane application. Int J Pharm. 2010;404:211–9.

381. Zhang W, Zhang Y, Löbler M, Schmitz K-P, Ahmad A, Pyykkö I, et al. Nuclear entry of hyperbranched polylysine nanoparticles into cochlear cells. Int J Nanomedicine. 2011;6:535–46.

382. Arslan N, Oguz H, Demirci M, Safak MA, Islam A, Kaytez SK, et al. Combined intratympanic and systemic use of steroids for idiopathic sudden sensorineural hearing loss. Otol Neurotol. 2011;32(3):393–7.

383. Shim HJ, Song SJ, Choi AY, Hyung Lee R, Yoon SW. Comparison of various treatment modalities for acute tinnitus. Laryngoscope. 2011;121(12):2619–25.

384. Wycherly BJ, Thompkins JJ, Kim HJ. Early posttreatment audiometry underestimates hearing recovery after intratympanic steroid treatment of sudden sensorineural hearing loss. Int J Otolaryngol. 2011;2011:465831.

385. Bielefeld EC, Hangauer D, Henderson D. Protection from impulse noise-induced hearing loss with novel Src-protein tyrosine kinase inhibitors. Neurosci Res. 2011;71:348–54.

386. Salt AN, Hartsock J, Plontke S, Lebel C, Piu F. Distribution of Dexamethasone and Preservation of Inner Ear Function following Intratympanic Delivery of a Gel-Based Formulation. Audiol Neurotol. 2011;16:323–35.

387. Piu F, Wang X, Fernandez R, Dellamary L, Harrop A, Ye Q, et al. OTO-104: A sustained-release dexamethasone hydrogel for the treatment of otic disorders. Otol Neurotol. 2011;32(1):171–9.

388. Wang X, Dellamary L, Fernandez R, Ye Q, LeBel C, Piu F. Principles of inner ear sustained release following intratympanic administration. Laryngoscope. 2011 Jan 26;121(2):385–91.

389. Delgado LP, Rodrigo JF, Pena PA. Intratympanic gentamicin in Meniere’s disease: our experience. J Laryngol Otol. 2011;125(4):363–9.

390. Buki B, Platz M, Haslwanter T, Junger H, Avan P. Results of electrocochleography in Meniere’s disease after successful vertigo control by single intratympanic gentamicin injection. Audiol Neurootol. 2011;16(1):49–54.

391. Alagic Z, Goiny M, Canlon B. Protection against acoustic trauma by direct application of D-methionine to the inner ear. Acta Otolaryngol. 2011;131:802–8.

392. Parham K. Can intratympanic dexamethasone protect against cisplatin ototoxicity in mice with age-related hearing loss? Otolaryngol - Head Neck Surg. 2011;

393. Zhou Y, Zheng H, Zhang Q, Campione PA. Early transtympanic steroid injection in patients with “poor prognosis” idiopathic sensorineural sudden hearing loss. ORL J Otorhinolaryngol Relat Spec. 2011;73(1):31–7.

394. Fu Y, Zhao H, Zhang T, Chi F. Intratympanic dexamethasone as initial therapy for idiopathic sudden sensorineural hearing loss: Clinical evaluation and laboratory investigation. Auris Nasus Larynx. 2011;38(2):165–71.

395. Wang X, Fernandez R, Dellamary L, Harrop A, Ye Q, Lichter J, et al. Pharmacokinetics of Dexamethasone Solution following Intratympanic Injection in Guinea Pig and Sheep. Audiol Neurotol. 2011;16:233–41.

396. Park MK, Lee CK, Park KH, Lee JD, Lee CG, Lee BD. Simultaneous versus subsequent intratympanic dexamethasone for idiopathic sudden sensorineural hearing loss. Otolaryngol Head Neck Surg. 2011;145(6):1016–21.

397. Khaimook W, Jantarapattana K. Therapy of idiopathic sudden sensorineural hearing loss with intratympanic steroid injection. J Med Assoc Thai. 2011;94(12):1495–9.

398. Dispenza F, Amodio E, De Stefano A, Gallina S, Marchese D, Mathur N, et al. Treatment of sudden sensorineural hearing loss with transtympanic injection of steroids as single therapy: a randomized clinical study. Eur Arch Otorhinolaryngol. 2011;268(9):1273–8.

399. Verdonck J, Desloovere C. Intratympanic lidocaine instillation for Meniere’s disease. B-ENT. 2011;7(3):157–64.

400. Moon IS, Lee JD, Kim J, Hong S-J, Lee W-S. Intratympanic dexamethasone is an effective method as a salvage treatment in refractory sudden hearing loss. Otol Neurotol. 2011;32(9):1432–6.

401. Murphy D, Daniel SJ. Intratympanic dexamethasone to prevent cisplatin ototoxicity: a guinea pig model. Otolaryngol Head Neck Surg. 2011;145(3):452–7.

402. Gouveris H, Schuler-Schmidt W, Mewes T, Mann W. Intratympanic dexamethasone/hyaluronic acid mix as an adjunct to intravenous steroid and vasoactive treatment in patients with severe idiopathic sudden sensorineural hearing loss. Otol Neurotol. 2011;32(5):756–60.

403. Tsai Y-J, Liang J-G, Wu W-B, Ding Y-F, Chiang RP-Y, Wu S-M. Intratympanic injection with dexamethasone for sudden sensorineural hearing loss. J Laryngol Otol. 2011;125(2):133–7.

404. Dallan I, Fortunato S, Casani AP, Panicucci E, Berrettini S, Lenzi R, et al. Intratympanic methylprednisolone as first-line therapy in sudden sensorineural hearing loss: preliminary results from a case-control series. J Laryngol Otol. 2011;125(10):1004–8.

405. Wu H-P, Chou Y-F, Yu S-H, Wang C-P, Hsu C-J, Chen P-R. Intratympanic steroid injections as a salvage treatment for sudden sensorineural hearing loss: a randomized, double-blind, placebo-controlled study. Otol Neurotol. 2011;32(5):774–9.

406. Bird PA, Murray DP, Zhang M, Begg EJ. Intratympanic versus intravenous delivery of dexamethasone and dexamethasone sodium phosphate to cochlear perilymph. Otol Neurotol. 2011;32(6):933–6.

407. Rauch SD, Halpin CF, Antonelli PJ, Babu S, Carey JP, Gantz BJ, et al. Oral vs intratympanic corticosteroid therapy for idiopathic sudden sensorineural hearing loss: a randomized trial. JAMA. 2011;305(20):2071–9.

408. Muehlmeier G, Biesinger E, Maier H. Safety of intratympanic injection of AM-101 in patients with acute inner ear tinnitus. Audiol Neurootol. 2011;16(6):388–97.

409. Kaur T, Mukherjea D, Sheehan K, Jajoo S, Rybak LP, Ramkumar V. Short interfering RNA against STAT1 attenuates cisplatin-induced ototoxicity in the rat by suppressing inflammation. Cell Death Dis. 2011;2:e180.

410. Kakehata S, Sasaki A, Futai K, Kitani R, Shinkawa H. Daily short-term intratympanic dexamethasone treatment alone as an initial or salvage treatment for idiopathic sudden sensorineural hearing loss. Audiol Neurootol. 2011;16(3):191–7.

411. Akil O, Seal RP, Burke K, Wang C, Alemi A, During M, et al. Article Restoration of Hearing in the VGLUT3 Knockout Mouse Using Virally Mediated Gene Therapy. 2012;

412. Fukui H, Wong HT, Beyer LA, Case BG, Swiderski DL, Di Polo A, et al. BDNF gene therapy induces auditory nerve survival and fiber sprouting in deaf Pou4f3 mutant mice. Sci Rep. 2012;2:838.

413. Atkinson PJ, Wise AK, Flynn BO, Nayagam BA, Hume CR, O’Leary SJ, et al. Neurotrophin Gene Therapy for Sustained Neural Preservation after Deafness. Kirchmair R, editor. PLoS One. 2012 Dec 17;7(12):e52338.

414. Bas E, Van De Water TR, Gupta C, Dinh J, Vu L, Martinez-Soriano F, et al. Efficacy of three drugs for protecting against gentamicin-induced hair cell and hearing losses. Br J Pharmacol. 2012;166(6):1888–904.

415. Wang Y, Ren J, Lu Y, Yin T, Xie D. Evaluation of intratympanic dexamethasone for treatment of refractory sudden sensorineural hearing loss. J Zhejiang Univ Sci B. 2012;13(3):203–8.

416. Buckiová D, Ranjan S, Newman TA, Johnston AH, Sood R, Kinnunen PK, et al. Minimally invasive drug delivery to the cochlea through application of nanoparticles to the round window membrane. Nanomedicine. 2012;

417. Lambert PR, Nguyen S, Maxwell KS, Tucci DL, Lustig LR, Fletcher M, et al. A randomized, double-blind, placebo-controlled clinical study to assess safety and clinical activity of OTO-104 given as a single intratympanic injection in patients with unilateral Meniere’s disease. Otol Neurotol. 2012;33(7):1257–65.

418. Yu J, Xiang M, Wu H, Shen C. Intratympanic injection of shRNA-expressing lentivirus causes gene silencing in the inner ear in chicken. Neurosci Lett. 2012;510(2):132–7.

419. Lim HJ, Kim YT, Choi SJ, Lee J Bin, Park HY, Park K, et al. Efficacy of 3 different steroid treatments for sudden sensorineural hearing loss: a prospective, randomized trial. Otolaryngol Head Neck Surg. 2013;148(1):121–7.

420. Suzuki H, Hashida K, Nguyen K-H, Hohchi N, Katoh A, Koizumi H, et al. Efficacy of intratympanic steroid administration on idiopathic sudden sensorineural hearing loss in comparison with hyperbaric oxygen therapy. Laryngoscope. 2012;122(5):1154–7.

421. Barreto MA de SC, Silva IB de O, de Oliveira CACP, Bahmad FJ. Intratympanic corticotherapy and tinnitus control after sudden hearing loss. Int Tinnitus J. 2012;17(2):186–93.

422. Maeda Y, Fukushima K, Kariya S, Orita Y, Nishizaki K. Intratympanic dexamethasone up-regulates Fkbp5 in the cochleae of mice in vivo. Acta Otolaryngol. 2012;132(1):4–9.

423. Ozdogan F, Ensari S, Cakir O, Ozcan KM, Koseoglu S, Ozdas T, et al. Investigation of the cochlear effects of intratympanic steroids administered following acoustic trauma. Laryngoscope. 2012;122(4):877–82.

424. Calli C, Pinar E, Oncel S, Alper Bagriyanik H, Umut Sakarya E. Recovery of hearing in Cisplatin-induced ototoxicity in the Guinea pig with intratympanic dexamethasone. Indian J Otolaryngol Head Neck Surg. 2012;64(1):46–50.

425. Casani A Pietro, Piaggi P, Cerchiai N, Seccia V, Franceschini SS, Dallan I. Intratympanic treatment of intractable unilateral Meniere disease: gentamicin or dexamethasone? A randomized controlled trial. Otolaryngol Head Neck Surg. 2012;146(3):430–7.

426. Mood ZA, Daniel SJ. Use of a microendoscope for transtympanic drug delivery to the round window membrane in chinchillas. Otol Neurotol. 2012;33(8):1292–6.

427. Barriat S, van Wijck F, Staecker H, Lefebvre PP. Intratympanic steroid therapy using the Silverstein MicrowickTM for refractory sudden sensorineural hearing loss increases speech intelligibility. Audiol Neurootol. 2012;17(2):105–11.

428. Landry TG, Fallon JB, Wise AK, Shepherd RK. Chronic neurotrophin delivery promotes ectopic neurite growth from the spiral ganglion of deafened cochleae without compromising the spatial selectivity of cochlear implants. J Comp Neurol. 2013;521(12):2818–32.

429. Pan S, Wan J, Liu S, Zhang S, Xiong H, Zhou J, et al. Lentivirus carrying the Atoh1 gene infects normal rat cochlea. Neural Regen Res. 2013 Jun 15;8(17):1551–9.

430. Mizutari K, Fujioka M, Hosoya M, Bramhall N, Okano HJ, Okano H, et al. Notch Inhibition Induces Cochlear Hair Cell Regeneration and Recovery of Hearing after Acoustic Trauma. Neuron. 2013;77(1):58–69.

431. Wu N, Li M, Chen Z-T, Zhang X-B, Liu H-Z, Li Z, et al. In vivo delivery of Atoh1 gene to rat cochlea using a dendrimer-based nanocarrier. J Biomed Nanotechnol. 2013;9(10):1736–45.

432. Lee J, Ismail H, Lee JH, Kel G, O’Leary J, Hampson A, et al. Effect of both local and systemically administered dexamethasone on long-term hearing and tissue response in a Guinea pig model of cochlear implantation. Audiol Neurootol. 2013;18(6):392–405.

433. Jang CH, Cho YB, Choi H, Um J-Y, Wang P-C, Pak C. The effect of topically administered latanoprost on the cochlear blood flow and hearing. Int J Pediatr Otorhinolaryngol. 2013;77:981–5.

434. Li L, Ren J, Yin T, Liu W. Intratympanic dexamethasone perfusion versus injection for treatment of refractory sudden sensorineural hearing loss. Eur Arch Otorhinolaryngol. 2013;270(3):861–7.

435. Wasson J, Upile N, Pfleiderer A. Intratympanic gentamicin treatment for unilateral Meniere’s disease: long-term follow up of a proven regime. J Laryngol Otol. 2013;127(1):20–4.

436. Ming-Lung Li M-L, Lung-Cheng Lee L-C, Yuh-Ren Cheng Y-R, Ching-Hua Kuo C-H, Yuan-Fang Chou Y-F, Yuh-Shyang Chen Y-S, et al. A Novel Aerosol-Mediated Drug Delivery System for Inner Ear Therapy: Intratympanic Aerosol Methylprednisolone Can Attenuate Acoustic Trauma. IEEE Trans Biomed Eng. 2013 Sep;60(9):2450–60.

437. Chou Y-F, Chen P-R, Kuo I-J, Yu S-H, Wen Y-H, Wu H-P. Comparison of intermittent intratympanic steroid injection and near-continual transtympanic steroid perfusion as salvage treatments for sudden sensorineural hearing loss. Laryngoscope. 2013;123(9):2264–9.

438. Oishi N, Chen F-Q, Zheng H-W, Sha S-H. Intra-tympanic delivery of short interfering RNA into the adult mouse cochlea. Hear Res. 2013;296:36–41.

439. Paradis J, Hu A, Parnes LS. Endolymphatic sac surgery versus intratympanic gentamicin for the treatment of intractable Meniere’s disease: a retrospective review with survey. Otol Neurotol. 2013;34(8):1434–7.

440. Zhai F, Zhang R, Zhang T, Steyger PS, Dai C-F. Preclinical and Clinical Studies of Unrelieved Aural Fullness following Intratympanic Gentamicin Injection in Patients with Intractable Ménière’s Disease. Audiol Neurootol. 2013;18(5):297–306.

441. Martin Sanz E, Zschaeck C, Gonzalez M, Mato T, Rodriganez L, Barona R, et al. Control of vertigo after intratympanic corticoid therapy for unilateral Meniere’s disease: a comparison of weekly versus daily fixed protocols. Otol Neurotol. 2013;34(8):1429–33.

442. Grewal AS, Nedzelski JM, Chen JM, Lin VYW. Dexamethasone uptake in the murine organ of Corti with transtympanic versus systemic administration. J Otolaryngol Head Neck Surg. 2013;42:19.

443. Shafik AG, Elkabarity RH, Thabet MT, Soliman NB, Kalleny NK. Effect of intratympanic dexamethasone administration on cisplatin-induced ototoxicity in adult guinea pigs. Auris Nasus Larynx. 2013;40(1):51–60.

444. Koltsidopoulos P, Bibas A, Sismanis A, Tzonou A, Seggas I. Intratympanic and systemic steroids for sudden hearing loss. Otol Neurotol. 2013;34(4):771–6.

445. Labatut T, Daza MJ, Alonso A. Intratympanic steroids as primary initial treatment of idiopathic sudden sensorineural hearing loss. The Hospital Universitario Ramon y Cajal experience and review of the literature. Eur Arch Otorhinolaryngol. 2013;270(11):2823–32.

446. Filipo R, Attanasio G, Cagnoni L, Masci E, Russo FY, Cartocci G, et al. Long-term results of intratympanic prednisolone injection in patients with idiopathic sudden sensorineural hearing loss. Acta Otolaryngol. 2013;133(9):900–4.

447. Cvorovic L, Jovanovic MB, Milutinovic Z, Arsovic N, Djeric D. Randomized prospective trial of hyperbaric oxygen therapy and intratympanic steroid injection as salvage treatment of sudden sensorineural hearing loss. Otol Neurotol. 2013;34(6):1021–6.

448. Dispenza F, De Stefano A, Costantino C, Marchese D, Riggio F. Sudden sensorineural hearing loss: results of intratympanic steroids as salvage treatment. Am J Otolaryngol. 2013;34(4):296–300.

449. Baysal E, Tunc O, Baglam T, Durucu C, Oz A, Karatas ZA, et al. Systemic steroid versus combined systemic and intratympanic steroid treatment for sudden sensorineural hearing loss. J Craniofac Surg. 2013;24(2):432–4.

450. Gundogan O, Pinar E, Imre A, Ozturkcan S, Cokmez O, Yigiter AC. Therapeutic efficacy of the combination of intratympanic methylprednisolone and oral steroid for idiopathic sudden deafness. Otolaryngol Head Neck Surg. 2013;149(5):753–8.

451. Sozen E, Erol SB, Yildirim O, Coskun BU, Basak T, Kayhan FT, et al. Local and systemic effects of low-dose transtympanic methotrexate: in vivo animal study. J Laryngol Otol. 2013;127(2):148–52.

452. Gabra N, Saliba I. The effect of intratympanic methylprednisolone and gentamicin injection on Meniere’s disease. Otolaryngol Head Neck Surg. 2013;148(4):642–7.

453. Li J, Yu L, Xia R, Gao F, Luo W, Jing Y. Postauricular hypodermic injection to treat inner ear disorders: experimental feasibility study using magnetic resonance imaging and pharmacokinetic comparison. J Laryngol Otol. 2013;127(3):239–45.

454. Hütten M, Dhanasingh A, Hessler R, Stöver T, Esser K-H, Möller M, et al. In Vitro and In Vivo Evaluation of a Hydrogel Reservoir as a Continuous Drug Delivery System for Inner Ear Treatment. Sokolowski B, editor. PLoS One. 2014 Aug 8;9(8):e104564.

455. Yu Q, Wang Y, Chang Q, Wang J, Gong S, Li H, et al. Virally-expressed connexin26 restores gap junction function in the cochlea of conditional Gjb2 knockout mice HHS Public Access. Gene Ther. 2014;21(1):71–80.

456. Nakagawa T, Kumakawa K, Usami S, Hato N, Tabuchi K, Takahashi M, et al. A randomized controlled clinical trial of topical insulin-like growth factor-1 therapy for sudden deafness refractory to systemic corticosteroid treatment. BMC Med. 2014;12:219.

457. Zou J, Sood R, Zhang Y, Kinnunen PK, Pyykkö I. Pathway and morphological transformation of liposome nanocarriers after release from a novel sustained inner-ear delivery system. Nanomedicine. 2014;(14):2143–55.

458. Plontke SK, Glien A, Rahne T, Mader K, Salt AN. Controlled release dexamethasone implants in the round window niche for salvage treatment of idiopathic sudden sensorineural hearing loss. Otol Neurotol. 2014;35(7):1168–71.

459. Ward JA, Sidell DR, Nassar M, Reece AL, Choo DI. Safety of cidofovir by intratympanic delivery technique. Antivir Ther. 2014;19(1):97–105.

460. Suckfuell M, Lisowska G, Domka W, Kabacinska A, Morawski K, Bodlaj R, et al. Efficacy and safety of AM-111 in the treatment of acute sensorineural hearing loss: a double-blind, randomized, placebo-controlled phase II study. Otol Neurotol. 2014;35(8):1317–26.

461. van de Heyning P, Muehlmeier G, Cox T, Lisowska G, Maier H, Morawski K, et al. Efficacy and safety of AM-101 in the treatment of acute inner ear tinnitus--a double-blind, randomized, placebo-controlled phase II study. Otol Neurotol. 2014;35(4):589–97.

462. Casani AP, Cerchiai N, Navari E, Dallan I, Piaggi P, Sellari-Franceschini S. Intratympanic gentamicin for Meniere’s disease: short- and long-term follow-up of two regimens of treatment. Otolaryngol Head Neck Surg. 2014;150(5):847–52.

463. Quaglieri S, Gatti O, Rebecchi E, Manfrin M, Tinelli C, Mira E, et al. Intratympanic gentamicin treatment “as needed” for Meniere’s disease. Long-term analysis using the Kaplan-Meier method. Eur Arch Otorhinolaryngol. 2014;271(6):1443–9.

464. Bremer HG, van Rooy I, Pullens B, Colijn C, Stegeman I, van der Zaag-Loonen HJ, et al. Intratympanic gentamicin treatment for Meniere’s disease: a randomized, double-blind, placebo-controlled trial on dose efficacy - results of a prematurely ended study. Trials. 2014;15:328.

465. Kim SH, Jung SY, Kim MG, Byun JY, Park MS, Yeo SG. Comparison of steroid administration methods in patients with idiopathic sudden sensorineural hearing loss: a retrospective observational study. Clin Otolaryngol. 2015;40(3):183–90.

466. Park SH, Park C, Seo JY, Cho JH, Moon IS. How long should patients remain in the supine treatment position after intratympanic dexamethasone injection?. Laryngoscope. 2014;124(12):2807–10.

467. Albu S, Chirtes F. Intratympanic dexamethasone plus melatonin versus melatonin only in the treatment of unilateral acute idiopathic tinnitus. Am J Otolaryngol. 2014;35(5):617–22.

468. Filipo R, Attanasio G, Russo FY, Cartocci G, Musacchio A, De Carlo A, et al. Oral versus short-term intratympanic prednisolone therapy for idiopathic sudden hearing loss. Audiol Neurootol. 2014;19(4):225–33.

469. Derebery MJ, Fisher LM, Voelker CCJ, Calzada A. An open label study to evaluate the safety and efficacy of intratympanic golimumab therapy in patients with autoimmune inner ear disease. Otol Neurotol. 2014;35(9):1515–21.

470. Oue S, Jervis-Bardy J, Stepan L, Chong S, Shaw C-KL. Efficacy of low-dose intratympanic dexamethasone as a salvage treatment for idiopathic sudden sensorineural hearing loss: the Modbury Hospital experience. J Laryngol Otol. 2014;128 Suppl:S27-30.

471. Erdur O, Kayhan FT, Cirik AA. Effectiveness of intratympanic dexamethasone for refractory sudden sensorineural hearing loss. Eur Arch Otorhinolaryngol. 2014;271(6):1431–6.

472. Wang G-P, Guo J-Y, Peng Z, Liu Y-Y, Xie J, Gong S-S. Adeno-associated virus-mediated gene transfer targeting normal and traumatized mouse utricle. Gene Ther. 2014;21:958–66.

473. Kurioka T, Mizutari K, Niwa K, Fukumori T, Inoue M, Hasegawa M, et al. Hyaluronic acid pretreatment for Sendai virus-mediated cochlear gene transfer. Gene Ther. 2016;23(2):187–95.

474. Liu Y, Jolly C, Braun S, Janssen T, Scherer E, Steinhoff J, et al. Effects of a dexamethasone-releasing implant on cochleae: A functional, morphological and pharmacokinetic study. Hear Res. 2015;327:89–101.

475. Gillespie LN, Zanin MP, Shepherd RK. Cell-based neurotrophin treatment supports long-term auditory neuron survival in the deaf guinea pig. J Control Release. 2015;198:26–34.

476. Watada Y, Yamashita D, Toyoda M, Tsuchiya K, Hida N, Tanimoto A, et al. Magnetic resonance monitoring of superparamagnetic iron oxide (SPIO)-labeled stem cells transplanted into the inner ear. Neurosci Res. 2015;95:21–6.

477. Tandon V, Kang WS, Spencer AJ, Kim ES, Pararas EEL, McKenna MJ, et al. Microfabricated infuse-withdraw micropump component for an integrated inner-ear drug-delivery platform. Biomed Microdevices. 2015;17(2):37.

478. Chang Q, Wang J, Li Q, Kim Y, Zhou B, Wang Y, et al. Virally mediated Kcnq1 gene replacement therapy in the immature scala media restores hearing in a mouse model of human Jervell and Lange-Nielsen deafness syndrome. EMBO Mol Med. 2015;7:1077–86.

479. Kim D-K, Park S-N, Park K-H, Park CW, Yang K-J, Kim J-D, et al. Development of a drug delivery system for the inner ear using poly(amino acid)-based nanoparticles. Drug Deliv. 2015 Apr 3;22(3).

480. Sircoglou J, Gehrke M, Tardivel M, Siepmann F, Siepmann J, Vincent C. Trans-Oval-Window Implants, A New Approach for Drug Delivery to the Inner Ear: Extended Dexamethasone Release From Silicone-based Implants. Otol Neurotol. 2015;36(9):1572–9.

481. She W, Lv L, Du X, Li H, Dai Y, Lu L, et al. Long-term effects of intratympanic methylprednisolone perfusion treatment on intractable Meniere’s disease. J Laryngol Otol. 2015;129(3):232–7.

482. Sun C, Wang X, Zheng Z, Chen D, Wang X, Shi F, et al. A single dose of dexamethasone encapsulated in polyethylene glycol-coated polylactic acid nanoparticles attenuates cisplatin-induced hearing loss following round window membrane administration. Int J Nanomedicine. 2015;10:3567–79.

483. Wang J, Wang Y, Chen X, Zhang P, Shi Z, Wen L, et al. Histone deacetylase inhibitor sodium butyrate attenuates gentamicin-induced hearing loss in vivo. Am J Otolaryngol. 2015;36(2):242–8.

484. Gao G, Liu Y, Zhou C-H, Jiang P, Sun J-J. Solid lipid nanoparticles loaded with edaravone for inner ear protection after noise exposure. Chin Med J (Engl). 2015;128(2):203–9.

485. Staecker H, Maxwell KS, Morris JR, van de Heyning P, Morawski K, Reintjes F, et al. Selecting appropriate dose regimens for AM-101 in the intratympanic treatment of acute inner ear tinnitus. Audiol Neurootol. 2015;20(3):172–82.

486. Engmer Berglin C, Videhult Pierre P, Ekborn A, Bramer T, Edsman K, Hultcrantz M, et al. Local treatment of the inner ear: a study of three different polymers aimed for middle ear administration. Acta Otolaryngol. 2015;135(10):985–94.

487. Zou J, Hannula M, Misra S, Feng H, Labrador RH, Aula AS, et al. Micro CT visualization of silver nanoparticles in the middle and inner ear of rat and transportation pathway after transtympanic injection. J Nanobiotechnology. 2015;13:5.

488. Watson GJ, Nelson C, Irving RM. Is low-dose intratympanic gentamicin an effective treatment for Meniere’s disease: the Birmingham experience. J Laryngol Otol. 2015;129(10):970–3.

489. Nakache G, Migirov L, Trommer S, Drendel M, Wolf M, Henkin Y. Steroid-based treatments for patients with total sudden sensorineural hearing loss. Acta Otolaryngol. 2015;135(9):907–13.

490. Zulueta-Santos C, Berumen OD, Manrique-Huarte R, Perez-Fernandez N. The effect of intra-tympanic dexamethasone on the vestibular function in patients with recurrent vertigo. Acta Otolaryngol. 2015;135(12):1253–8.

491. Rah YC, Han JJ, Park J, Choi BY, Koo J-W. Management of intractable Meniere’s disease after intratympanic injection of gentamicin. Laryngoscope. 2015;125(4):972–8.

492. Liu B, Leng Y, Shi H, Zhou R, Liu J, Zhang W, et al. Modified titration intratympanic gentamicin injection for unilateral intractable Meniere’s disease. J Huazhong Univ Sci Technolog Med Sci. 2015;35(5):747–51.

493. Marques P, Manrique-Huarte R, Perez-Fernandez N. Single intratympanic gentamicin injection in Meniere’s disease: VOR change and prognostic usefulness. Laryngoscope. 2015;125(8):1915–20.

494. Alexander TH, Harris JP, Nguyen QT, Vorasubin N. Dose Effect of Intratympanic Dexamethasone for Idiopathic Sudden Sensorineural Hearing Loss: 24 mg/mL Is Superior to 10 mg/mL. Otol Neurotol. 2015;36(8):1321–7.

495. Yang HC, Cho YB, Jang CH, Cho H-H. Efficacy of Concomitant Intratympanic Steroid Injection for Sudden Deafness According to Initial Hearing Loss. Otol Neurotol. 2015;36(10):1604–9.

496. Ren H, Yin T, Lu Y, Kong W, Ren J. Intratympanic dexamethasone injections for refractory Meniere’ s disease. Int J Clin Exp Med. 2015;8(4):6016–23.

497. Albu S, Chirtes F, Trombitas V, Nagy A, Marceanu L, Babighian G, et al. Intratympanic dexamethasone versus high dosage of betahistine in the treatment of intractable unilateral Meniere disease. Am J Otolaryngol. 2015;36(2):205–9.

498. Tsai H-T, Hsueh N, Huang C-M, Lin H-C. Intratympanic steroid injection as a first-line therapy in uremia patients with sudden sensorineural hearing loss. Acta Otolaryngol. 2015;135(8):786–90.

499. Han MA, Back SA, Kim HL, Park SY, Yeo SW, Park SN. Therapeutic Effect of Dexamethasone for Noise-induced Hearing Loss: Systemic Versus Intratympanic Injection in Mice. Otol Neurotol. 2015;36(5):755–62.

500. Martin-Sanz E, Esteban-Sanchez J, Rodriganez-Riesco L, Sanz-Fernandez R. Transitory effect on endolymphatic hydrops of the intratympanic steroids for Meniere’s disease. Laryngoscope. 2015;125(5):1183–8.

501. Liu B, Zhang S, Leng Y, Zhou R, Liu J, Kong W. Intratympanic injection in delayed endolymphatic hydrops. Acta Otolaryngol. 2015;135(10):1016–21.

502. Gunel C, Basal Y, Toka A, Eryilmaz A, Kurt Omurlu I. Efficacy of low-dose intratympanic dexamethasone for sudden hearing loss. Auris Nasus Larynx. 2015;42(4):284–7.

503. Wise AK, Tan J, Wang Y, Caruso F, Shepherd RK. Improved Auditory Nerve Survival with Nanoengineered Supraparticles for Neurotrophin Delivery into the Deafened Cochlea. PLoS One. 2016;11(10):e0164867.

504. Salt AN, Hartsock JJ, Gill RM, King E, Kraus FB, Plontke SK. Perilymph pharmacokinetics of locally-applied gentamicin in the guinea pig. Hear Res. 2016;342:101–11.

505. Astolfi L, Simoni E, Giarbini N, Giordano P, Pannella M, Hatzopoulos S, et al. Cochlear implant and inflammation reaction: Safety study of a new steroid-eluting electrode. Hear Res. 2016;336:44–52.

506. Liu Y, Jolly C, Braun S, Stark T, Scherer E, Plontke SK, et al. In vitro and in vivo pharmacokinetic study of a dexamethasone-releasing silicone for cochlear implants. Eur Arch Otorhinolaryngol. 2016;273(7):1745–53.

507. Nguyen Y, Celerier C, Pszczolinski R, Claver J, Blank U, Ferrary E, et al. Superparamagnetic nanoparticles as vectors for inner ear treatments: Driving and toxicity evaluation. In: Acta Oto-Laryngologica. 2016. p. 402–8.

508. Shu Y, Tao Y, Li W, Shen J, Wang Z, Chen Z-Y. Adenovirus Vectors Target Several Cell Subtypes of Mammalian Inner Ear In Vivo. Neural Plast. 2016;2016:9409846.

509. Shu Y, Tao Y, Wang Z, Tang Y, Li H, Dai P, et al. Identification of Adeno-Associated Viral Vectors That Target Neonatal and Adult Mammalian Inner Ear Cell Subtypes. Hum Gene Ther. 2016;27(9):687–99.

510. Gokcan MK, Mulazimoglu S, Ocak E, Can P, Caliskan M, Besalti O, et al. Study of mouse induced pluripotent stem cell transplantation intoWistar albino rat cochleae after hair cell damage. Turkish J Med Sci. 2016;46(5):1603–10.

511. Barboza LCMJ, Lezirovitz K, Zanatta DB, Strauss BE, Mingroni-Netto RC, Oiticica J, et al. Transplantation and survival of mouse inner ear progenitor/stem cells in the organ of Corti after cochleostomy of hearing-impaired guinea pigs: preliminary results. Brazilian J Med Biol Res = Rev Bras Pesqui medicas e Biol. 2016;49(4):e5064.

512. Chien WW, Isgrig K, Roy S, Belyantseva IA, Drummond MC, May LA, et al. Gene Therapy Restores Hair Cell Stereocilia Morphology in Inner Ears of Deaf Whirler Mice. Mol Ther. 2016;24(1):17–25.

513. Kang WS, Nguyen K, Mckenna CE, Sewell WF, Mckenna MJ, Jung DH. Intracochlear Drug Delivery through the Oval Window in Fresh Cadaveric Human Temporal Bones HHS Public Access. Otol Neurotol. 2016;37(3):218–22.

514. Plontke SK, Hartsock JJ, Gill RM, Salt AN. Intracochlear Drug Injections through the Round Window Membrane: Measures to Improve Drug Retention. Audiol Neurotol. 2016;21(2):72–9.

515. Tandon V, Kang WS, Robbins TA, Spencer AJ, Kim ES, Mckenna MJ, et al. Microfabricated Reciprocating Micropump for Intracochlear Drug Delivery with Integrated Drug/Fluid Storage and Electronically Controlled Dosing HHS Public Access. Lab Chip. 2016;16(5):829–46.

516. Lichtenhan JT, Hartsock J, Dornhoffer JR, Donovan KM, Salt AN. Drug delivery into the cochlear apex: Improved control to sequentially affect finely spaced regions along the entire length of the cochlear spiral. J Neurosci Methods. 2016;273:201–9.

517. Lavigne P, Lavigne F, Saliba I. Sustained Inner Ear Steroid Delivery via Bioabsorbable Stent. Otolaryngol Neck Surg. 2016 Oct 22;155(4):649–53.

518. Suzuki J, Corfas G, Liberman MC. Round-window delivery of neurotrophin 3 regenerates cochlear synapses after acoustic overexposure. Sci Rep. 2016;6:24907.

519. Sidell D, Ward JA, Pordal A, Quimby C, Nassar M, Choo DI. Combination therapies using an intratympanic polymer gel delivery system in the guinea pig animal model: A safety study. Int J Pediatr Otorhinolaryngol. 2016;84:132–6.

520. Takeda H, Kurioka T, Kaitsuka T, Tomizawa K, Matsunobu T, Hakim F, et al. Protein transduction therapy into cochleae via the round window niche in guinea pigs. Mol Ther - Methods Clin Dev. 2016;3:16055.

521. El Kechai N, Mamelle E, Nguyen Y, Huang N, Nicolas V, Chaminade P, et al. Hyaluronic acid liposomal gel sustains delivery of a corticoid to the inner ear. J Control Release. 2016;226:248–57.

522. Yu D, Sun C, Zheng Z, Wang X, Chen D, Wu H, et al. Inner ear delivery of dexamethasone using injectable silk-polyethylene glycol (PEG) hydrogel. Int J Pharm. 2016;503(1–2):229–37.

523. Honeder C, Zhu C, Schopper H, Gausterer JC, Walter M, Landegger LD, et al. Effects of sustained release dexamethasone hydrogels in hearing preservation cochlear implantation. Hear Res. 2016;341:43–9.

524. Harrop-Jones A, Wang X, Fernandez R, Dellamary L, Ryan AF, LeBel C, et al. The Sustained-Exposure Dexamethasone Formulation OTO-104 Offers Effective Protection against Noise-Induced Hearing Loss. Audiol Neurootol. 2016;21(1):12–21.

525. Wen X, Ding S, Cai H, Wang J, Wen L, Yang F, et al. Nanomedicine strategy for optimizing delivery to outer hair cells by surface-modified poly(lactic/glycolic acid) nanoparticles with hydrophilic molecules. Int J Nanomedicine. 2016;11:5959–69.

526. Youm I, Musazzi UM, Gratton MA, Murowchick JB, Youan BBC. Label-Free Ferrocene-Loaded Nanocarrier Engineering for In Vivo Cochlear Drug Delivery and Imaging. J Pharm Sci. 2016;105(10):3162–71.

527. Celis-Aguilar E, Hinojosa-Gonzalez R, Vales-Hidalgo O, Coutinho-Toledo H. Refractory episodic vertigo: role of intratympanic gentamicin and vestibular evoked myogenic potentials. Braz J Otorhinolaryngol. 2016;82(6):668–73.

528. Sam G, Chung DW, van der Hoeven R, Verweij S, Becker M. The effect of intratympanic gentamicin for treatment of Meniere’s disease on lower frequency hearing. Int J Clin Pharm. 2016;38(4):780–3.

529. Junet P, Karkas A, Dumas G, Quesada JL, Schmerber S. Vestibular results after intratympanic gentamicin therapy in disabling Meniere’s disease. Eur Arch Otorhinolaryngol. 2016;273(10):3011–8.

530. Ozel HE, Ozdogan F, Gurgen SG, Esen E, Genc S, Selcuk A. Comparison of the protective effects of intratympanic dexamethasone and methylprednisolone against cisplatin-induced ototoxicity. J Laryngol Otol. 2016;130(3):225–34.

531. Heinrich U-R, Strieth S, Schmidtmann I, Stauber R, Helling K. Dexamethasone prevents hearing loss by restoring glucocorticoid receptor expression in the guinea pig cochlea. Laryngoscope. 2016;126(1):E29-34.

532. Ozel HE, Ozdogan F, Gulsen Gurgen S, Esen E, Selcuk A, Genc S. Effect of transtympanic betamethasone delivery to the inner ear. Eur Arch Otorhinolaryngol. 2016;273(10):3053–61.

533. Swachia K, Sharma D, Singh J. Efficacy of oral vs. intratympanic corticosteroids in sudden sensorineural hearing loss. J Basic Clin Physiol Pharmacol. 2016;27(4):371–7.

534. Suzuki H, Koizumi H, Ohkubo J-I, Hohchi N, Ikezaki S, Kitamura T. Hearing outcome does not depend on the interval of intratympanic steroid administration in idiopathic sudden sensorineural hearing loss. Eur Arch Otorhinolaryngol. 2016;273(10):3101–7.

535. Berjis N, Soheilipour S, Musavi A, Hashemi SM. Intratympanic dexamethasone injection vs methylprednisolone for the treatment of refractory sudden sensorineural hearing loss. Adv Biomed Res. 2016;5:111.

536. Liu B, Leng Y, Zhou R, Liu J, Liu D, Zhang S-L, et al. Intratympanic steroids injection is effective for the treatment of drop attacks with Meniere’s disease and delayed endolymphatic hydrops: A retrospective study. Medicine (Baltimore). 2016;95(52):e5767.

537. Atrache Al Attrache N, Krstulovic C, Perez Guillen V, Morera Perez C, Perez Garrigues H. Response Over Time of Vertigo Spells to Intratympanic Dexamethasone Treatment in Meniere’s Disease Patients. J Int Adv Otol. 2016;12(1):92–7.

538. Kocak HE, Taskin U, Aydin S, Oktay MF, Altinay S, Celik DS, et al. Effects of ozone (O3) therapy on cisplatin-induced ototoxicity in rats. Eur Arch Otorhinolaryngol. 2016;273(12):4153–9.

539. Bekmez Bilmez ZE, Aydin S, Sanli A, Altintoprak N, Demir MG, Atalay Erdogan B, et al. Oxytocin as a protective agent in cisplatin-induced ototoxicity. Cancer Chemother Pharmacol. 2016;77(4):875–9.

540. Zhu BZ, Saleh J, Isgrig KT, Cunningham LL, Chien WW. Hearing Loss after Round Window Surgery in Mice Is due to Middle Ear Effusion. Audiol Neurotol. 2017;21(6):356–64.

541. Wick CC, Manzoor NF, McKenna C, Semaan MT, Megerian CA, C.C. W, et al. Long-term outcomes of endolymphatic sac shunting with local steroids for Meniere’s disease. Am J Otolaryngol. 2017;38(3):285–90.

542. Scheper V, Hessler R, Hutten M, Wilk M, Jolly C, Lenarz T, et al. Local inner ear application of dexamethasone in cochlear implant models is safe for auditory neurons and increases the neuroprotective effect of chronic electrical stimulation. PLoS One. 2017;12(8):e0183820.

543. Konerding WS, Janssen H, Hubka P, Tornøe J, Mistrik P, Wahlberg L, et al. Encapsulated cell device approach for combined electrical stimulation and neurotrophic treatment of the deaf cochlea. Hear Res. 2017;350:110–21.

544. Matsuoka AJ, Sayed ZA, Stephanopoulos N, Berns EJ, Wadhwani AR, Morrissey ZD, et al. Creating a stem cell niche in the inner ear using self-assembling peptide amphiphiles. PLoS One. 2017;12(12):e0190150.

545. Lee MY, Hackelberg S, Green KL, Lunghamer KG, Kurioka T, Loomis BR, et al. Survival of human embryonic stem cells implanted in the guinea pig auditory epithelium. Sci Rep. 2017;7:46058.

546. Gyorgy B, Sage C, Indzhykulian AA, Scheffer DI, Brisson AR, Tan S, et al. Rescue of Hearing by Gene Delivery to Inner-Ear Hair Cells Using Exosome-Associated AAV. Mol Ther. 2017;25(2):379–91.

547. Dai C, Lehar M, Sun DQ, Rvt LS, Carey JP, MacLachlan T, et al. Rhesus Cochlear and Vestibular Functions Are Preserved After Inner Ear Injection of Saline Volume Sufficient for Gene Therapy Delivery. JARO - J Assoc Res Otolaryngol. 2017;18(4):601–17.

548. Landegger LD, Pan B, Askew C, Wassmer SJ, Gluck SD, Galvin A, et al. A synthetic AAV vector enables safe and efficient gene transfer to the mammalian inner ear. Nat Biotechnol. 2017;35(3):280–4.

549. Emptoz A, Michel V, Lelli A, Akil O, Boutet de Monvel J, Lahlou G, et al. Local gene therapy durably restores vestibular function in a mouse model of Usher syndrome type 1G. Proc Natl Acad Sci U S A. 2017;114(36):9695–700.

550. Geng R, Omar A, Gopal SR, Chen DH-C, Stepanyan R, Basch ML, et al. Modeling and Preventing Progressive Hearing Loss in Usher Syndrome III. Sci Rep. 2017;7(1):13480.

551. Pfingst BE, Colesa DJ, Swiderski DL, Hughes AP, Strahl SB, Sinan M, et al. Neurotrophin Gene Therapy in Deafened Ears with Cochlear Implants: Long-term Effects on Nerve Survival and Functional Measures. J Assoc Res Otolaryngol. 2017 Dec 3;18(6):731–50.

552. Duarte M, Kanumuri V, Landegger L, Tarabichi O, Stankovic K, Brown M. Novel vector-mediated delivery of opsins to the cochlea. Otolaryngol - Head Neck Surg (United States). 2017;157(1 Supplement 1):P253–4.

553. Schendzielorz P, Vollmer M, Rak K, Wiegner A, Nada N, Radeloff K, et al. Adipose-derived stromal cells enhance auditory neuron survival in an animal model of sensory hearing loss. Cytotherapy. 2017;19(10):1197–207.

554. Chen J, Guan L, Zhu H, Xiong S, Zeng L, Jiang H, et al. Transplantation of mouse-induced pluripotent stem cells into the cochlea for the treatment of sensorineural hearing loss. Acta Otolaryngol. 2017;137(11):1136–42.

555. Lichtenhan JT, Hirose K, Buchman CA, Duncan RK, Salt AN. Direct administration of 2-Hydroxypropyl- Beta-Cyclodextrin into Guinea pig cochleae: Effects on physiological and histological measurements. Zeng F-G, editor. PLoS One. 2017 Apr 6;12(4):e0175236.

556. Muller M, Tisch M, Maier H, Lowenheim H. Reduction of permanent hearing loss by local glucocorticoid application : Guinea pigs with acute acoustic trauma. HNO. 2017;65(Suppl 1):59–67.

557. Fransson A. Structural changes in the inner ear over time studied in the experimentally deafened guinea pig. J Neurosci Res. 2017;95(3):869–75.

558. Pan B, Askew C, Galvin A, Heman-Ackah S, Asai Y, Indzhykulian A, et al. Gene therapy restores auditory and vestibular function in a mouse model for usher syndrome type 1C. Mol Ther. 2017;25(5 Supplement 1):352.

559. Shi X, Wu N, Zhang Y, Guo W, Lin C, Yang S. Adeno-associated virus transformation into the normal miniature pig and the normal guinea pigs cochlea via scala tympani. Acta Otolaryngol. 2017;1–7.

560. Pan B, Askew C, Galvin A, Heman-Ackah S, Asai Y, Indzhykulian AA, et al. Gene therapy restores auditory and vestibular function in a mouse model of Usher syndrome type 1c. Nat Biotechnol. 2017 Mar 6;35(3):264–72.

561. Mamelle E, Kechai N El, Granger B, Sterkers O, Bochot A, Agnely F, et al. Effect of a liposomal hyaluronic acid gel loaded with dexamethasone in a guinea pig model after manual or motorized cochlear implantation. Eur Arch Otorhinolaryngol. 2017;274(2):729–36.

562. Murillo-Cuesta S, Vallecillo NN, Cediel R, Celaya AM, Lassaletta L, Varela-Nieto I, et al. A Comparative Study of Drug Delivery Methods Targeted to the Mouse Inner Ear: Bullostomy Versus Transtympanic Injection. J Vis Exp. 2017;(121).

563. Kostal M, Drsata J, Blaha M, Lanska M, Chrobok V. Rheopheresis in treatment of idiopathic sensorineural sudden hearing loss. J Otolaryngol - Head Neck Surg. 2017;46(1):50.

564. Gur H, Alimoglu Y, Duzenli U, Korkmaz S, Inan S, Olgun L, et al. The effect of local application of insulin-like growth factor for prevention of inner-ear damage caused by electrode trauma. J Laryngol Otol. 2017;131(3):245–52.

565. Acioglu E, Yigit O, Onur F, Atas A, Server EA, Kara E. Ototoxicity associated with topical administration of diclofenac sodium as an otic drop: An experimental animal study. Int J Pediatr Otorhinolaryngol. 2017;98:110–5.

566. Zou J, Ostrovsky S, Israel L, Feng H, Kettunen M, Lellouche J. Efficient penetration of ceric ammonium nitrate oxidant-stabilized gamma-maghemite nanoparticles through the oval and round windows into the rat inner ear as demonstrated by MRI. J Biomed Mater Res - Part B Appl Biomater. 2017;105(7):1883–91.

567. Sale PJP, Uschakov A, Saief T, Rowe DP, Abbott CJ, Luu CD, et al. Cannula-based drug delivery to the guinea pig round window causes a lasting hearing loss that may be temporarily mitigated by BDNF. Hear Res. 2017;356:104–15.

568. Zou J, Feng H, Sood R, Kinnunen PKJ, Pyykko I. Biocompatibility of Liposome Nanocarriers in the Rat Inner Ear After Intratympanic Administration. Nanoscale Res Lett. 2017;12(1):372.

569. Loader B, Seemann R, Atteneder C, Sterrer E, Franz P. Sealing of the round and oval window niches with triamcinolone-soaked fascia as salvage surgical therapy in sudden sensorineural hearing loss. Acta Otolaryngol. 2017;137(9):923–7.

570. Martin-Saldana S, Palao-Suay R, Aguilar MR, Ramirez-Camacho R, San Roman J. Polymeric nanoparticles loaded with dexamethasone or alpha-tocopheryl succinate to prevent cisplatin-induced ototoxicity. Acta Biomater. 2017;53:199–210.

571. King EB, Shepherd RK, Brown DJ, Fallon JB. Gentamicin Applied to the Oval Window Suppresses Vestibular Function in Guinea Pigs. J Assoc Res Otolaryngol. 2017;18(2):291–9.

572. Kurabi A, Beasley KA, Chang L, McCann J, Pak K, Ryan AF, et al. Peptides actively transported across the tympanic membrane: Functional and structural properties. PLoS One. 2017;12(2):e0172158.

573. Edizer DT, Yigit O, Cinar Z, Gul M, Kara E, Yigitcan B, et al. Protective role of intratympanic nigella sativa oil against gentamicin induced hearing loss. Int J Pediatr Otorhinolaryngol. 2017;97:83–8.

574. Arnoldner C, Gausterer J, Schopper H, Honeder C, Nieratschker M. Evaluation of sustained release steroid-hydrogels in a model for noise-induced hearing loss. Otolaryngol - Head Neck Surg (United States). 2017;157(1 Supplement 1):P117.

575. Rogha M. Therapeutic effect of intra-tympanic dexamethasone-hyaluronic acid combination in sudden sensorineural hearing loss. Iran J Otorhinolaryngol. 2017;29(5):255–60.

576. Staecker H, Morelock M, Kramer T, Chrbolka P, Ahn JH, Meyer T, et al. Safety of Repeated-Dose Intratympanic Injections with AM-101 in Acute Inner Ear Tinnitus. Otolaryngol Head Neck Surg. 2017;157(3):478–87.

577. Meyer F, Rolland V, Bairati I, Guitton M, Fortin A. Randomized controlled trial to test the efficacy of transtympanic injections of a sodium thiosulfate gel to prevent cisplatin-induced ototoxicity. J Clin Oncol. 2017;35(15 Supplement 1).

578. Ramaswamy B, Roy S, Apolo AB, Shapiro B, Depireux DA, B. R, et al. Magnetic nanoparticle mediated steroid delivery mitigates cisplatin induced hearing loss. Front Cell Neurosci. 2017;11:268.

579. Cai H, Liang Z, Huang W, Wen L, Hui C, Liang Z, et al. Engineering PLGA nano-based systems through understanding the influence of nanoparticle properties and cell-penetrating peptides for cochlear drug delivery. Int J Pharm. 2017;532(1):55–65.

580. Ardic FN, Tumkaya F, Aykal K, Cabuk B, F.N. A, F. T, et al. Selective Window Application of Gentamicin+ Dexamethasone in Meniere’s Disease. J Int Adv Otol. 2017;13(2):243–6.

581. Liu Y, Renk E, Rauch S. Efficacy of Intratympanic Gentamicin in Meniere’s Disease With and Without Migraine. Otol Neurotol. 2017;38(7):1005–9.

582. Liu H, Zhang T, Wu Q, Zhang Y, Dai C. End-point indicators of low-dose intra-tympanic gentamicin in management of Ménière’s disease. Acta Otolaryngol. 2017 Feb 9;137(2):136–43.

583. North H, Freeman S, Wadeson A, Rutherford S, King A, Hammerbeck-Ward C. The efficacy of pre-operative intratympanic gentamicin in alleviating post-operative vestibular symptoms following surgical removal of vestibular schwannomas. J Laryngol Otol. 2017;131(6):E2.

584. Aydin S, Demir M, Oguztuzun S, Altintoprak N, Bilmez E, Gul A. GSTP1 levels in cisplatin-induced rat cochlea after alpha lipoic acid and oxytocin treatment. Indian J Otol. 2017;23(4):237–40.

585. Shim HJ, Lee ES, An YH, Kim DH, H.J. S, E.S. L, et al. Comparison of Long-Term Outcome of Intratympanic Dexamethasone Therapy between Acute Noise-Induced Tinnitus and Acute Idiopathic Tinnitus. J Int Adv Otol. 2017;13(1):53–60.

586. Choo O-S, Yang SM, Park HY, Lee J Bin, Jang JH, Choi SJ, et al. Differences in clinical characteristics and prognosis of sudden low- and high-frequency hearing loss. Laryngoscope. 2017 Aug 1;127(8):1878–84.

587. Dinh CT, Chen S, Dinh J, Goncalves S, Bas E, Padgett K, et al. Effects of Intratympanic Dexamethasone on High-Dose Radiation Ototoxicity In Vivo. Otol Neurotol. 2017;38(2):180–6.

588. Beyea J, Instrum R, Agrawal S. Intratympanic Dexamethasone in the Treatment of Meniere’s Disease: A Comparison of Two Techniques. Otol Neurotol. 2017;38(6):e173–8.

589. Dabiri Satri S, Gharibi R, Nejadian F, Yazdani N, Hoseinabadi R, Rezazadeh N, et al. Intratympanic injection of dexamethasone and electrocochleographic data in cases of definite one sided refractory meniere’s disease. Iran J Otorhinolaryngol. 2017;29(92):121–5.

590. Dai Y, Lu L, Hou J, Yang X, Li H, Yang Y, et al. Intratympanic methylprednisolone perfusion as a salvage treatment for profound idiopathic sudden sensorineural hearing loss. J Laryngol Otol. 2017;131(5):404–10.

591. Jumaily M, Faraji F. Intratympanic Triamcinolone and Dexamethasone in the Treatment of Meniere’s Syndrome. Otol Neurotol. 2017;38(3):386–91.

592. Punagi A, Pratama A, Kadir A, Savitri E. Light microscope study of intratympanic methylprednisolone effect on cisplatin induced ototoxicity. Trends Med Res. 2017;12(2):65–70.

593. Masoumi E, Dabiri S, Ashtiani M, Erfanian R, Sohrabpour S, Yazdani N, et al. Methylprednisolone versus dexamethasone for control of vertigo in patients with definite Meniere’s disease. Iran J Otorhinolaryngol. 2017;29(6):341–6.

594. Topf MC, Hsu DW, Adams DR, Zhan T, Pelosi S, Willcox TO, et al. Rate of tympanic membrane perforation after intratympanic steroid injection. Am J Otolaryngol. 2017;38(1):21–5.

595. Y. L, B. L, R. Z, J. L, D. L, S.-L. Z, et al. Repeated courses of intratympanic dexamethasone injection are effective for intractable Meniere’s disease. Acta Otolaryngol. 2017;137(2):154–60.

596. Yoo MH, Lim WS, Park JH, Kwon JK, Lee T-H, An Y-H, et al. Simultaneous versus Sequential Intratympanic Steroid Treatment for Severe-to-Profound Sudden Sensorineural Hearing Loss. Audiol Neurotol. 2016;21(6):399–405.

597. Omichi R, Maeda Y, Kariya S. Steroid upregulates fkbp5 expression in mice cochleae [presentation withdrawn]. Otolaryngol - Head Neck Surg (United States). 2017;157(1 Supplement 1):P261.

598. Okada M, Hato N, Nishio S-Y, Kitoh R, Ogawa K, Kanzaki S, et al. The effect of initial treatment on hearing prognosis in idiopathic sudden sensorineural hearing loss: a nationwide survey in Japan. Acta Otolaryngol. 2017;137(sup565):S30–3.

599. Gul A, Sengul E, Yilmaz B, Ozkurt FE, Akdag M, Keles A, et al. The protective effect of intratympanic dexamethasone on streptomycin ototoxicity in rats. Ear Nose Throat J. 2017;96(6):E12.

600. Kuthubutheen J, Joglekar S, Smith L, Friesen L, Smilsky K, Millman T, et al. The Role of Preoperative Steroids for Hearing Preservation Cochlear Implantation: Results of a Randomized Controlled Trial. Audiol Neurootol. 2017;22(4–5):292–302.

601. Sun W, Guo P, Ren T, Wang W. Magnetic resonance imaging of intratympanic gadolinium helps differentiate vestibular migraine from Meniere disease. Laryngoscope. 2017;127(10):2382–8.

602. Wang F, Lyu H, Zhao M, Sha Y, Zhang F, Cheng Y, et al. Assessment of Cochlea Endolymphatic Hydrops Using 3-D FLAIR and 3-D Real IR Sequence in Guinea Pigs via 3T MRI After Intratympanic Gadolinium: A Histopathological Comparison. Otol Neurotol. 2017;38(4):585–90.

603. Guneri EA, Olgun Y, Aslier M, Nuti D, Kirkim G, Mungan S, et al. Cochlear and Vestibular Effects of Combined Intratympanic Gentamicin and Dexamethasone. J Int Adv Otol. 2017 May 29;13(1):47–52.

604. Tunon Gomez M, Lobo Duro DR, Brea Alvarez B, Garcia-Berrocal JR. Diagnosis of endolymphatic hydrops by means of 3 T magnetic resonance imaging after intratympanic administration of gadolinium. Diagnostico del hidrops endolinfatico Median Reson Magn 3T tras Adm gadolinio intratimpanico. 2017;59(2):159–65.

605. Callejo A, Durochat A, Bressieux S, Saleur A, Chabbert C, Domenech Juan I, et al. Dose-dependent cochlear and vestibular toxicity of trans-tympanic cisplatin in the rat. Neurotoxicology. 2017;60:1–9.

606. Iwasa Y, Tsukada K, Fukuoka H. Evaluation of delayed endolymphatic hydrops using 3t magnetic resonance imaging. Otolaryngol - Head Neck Surg (United States). 2017;157(1 Supplement 1):P245–6.

607. Eren SB, Dogan R, Yenigun A, Veyseller B, Tugrul S, Ozturan O, et al. Evaluation of ototoxicity of intratympanic administration of Methotrexate in rats. Int J Pediatr Otorhinolaryngol. 2017;100:132–6.

608. Abayli C, Kul Y, Koten M, Karasalihoglu AR, Tas A, Yagiz R, et al. Possible Ototoxic Effects of Topical Rifamycin Application: An Electrophysiological and Ultrastructural Study. Turkish Arch Otorhinolaryngol. 2017;55(3):99–104.

609. Nhan C, Bezdjian A, Saha S, Prakash S, Nguyen LHP, Daniel SJ. Safety of transtympanic application of probiotics in a chinchilla animal model. J Otolaryngol - Head Neck Surg. 2017;46(1):63.

610. Ul Shamas I. Short term results of intra tympanic gentamicin and dexamethasone on hearing and tinnitus in meniere’s disease: A case control study. Int Tinnitus J. 2017;21(1):21–3.

611. Belhassen S, Alzahrani M, Nader M-E, Gaboury L, Saliba I. Study of Methylene Blue Ototoxicity in the Guinea Pig. J Clin Med Res. 2017;9(11):900–6.

612. Vural A, Sahin MI, Aydin M, Gundogdu R, Arli T, Okuducu H, et al. The Effect of Nystatin Solution on Otoacoustic Emissions in Rats. J Int Adv Otol. 2017;13(1):105–9.

613. Suzuki J, Hashimoto K, Xiao R, Vandenberghe LH, Liberman MC. Cochlear gene therapy with ancestral AAV in adult mice: complete transduction of inner hair cells without cochlear dysfunction. Sci Rep. 2017;7:45524.

614. Guo J-Y, Liu Y-Y, Qu T-F, Peng Z, Xie J, Wang G-P, et al. Cochleovestibular gene transfer in neonatal mice by canalostomy. Neuroreport. 2017;28(11):682–8.

615. Isgrig K, Shteamer JW, Belyantseva IA, Drummond MC, Fitzgerald TS, Vijayakumar S, et al. Gene Therapy Restores Balance and Auditory Functions in a Mouse Model of Usher Syndrome. Mol Ther. 2017;25(3):780–91.

616. Yamaguchi T, Yoneyama M, Ogita K. Calpain inhibitor alleviates permanent hearing loss induced by intense noise by preventing disruption of gap junction-mediated intercellular communication in the cochlear spiral ligament. Eur J Pharmacol. 2017;803:187–94.

617. Lyu A-R, Kim DH, Lee SH, Shin D-S, Shin S-A, Park Y-H. Effects of dexamethasone on intracochlear inflammation and residual hearing after cochleostomy: A comparison of administration routes. PLoS One. 2018;13(3):e0195230.

618. Yoshimura H, Shibata SB, Ranum PT, Smith RJH. Enhanced viral-mediated cochlear gene delivery in adult mice by combining canal fenestration with round window membrane inoculation. Sci Rep. 2018;8(1):2980.

619. Gao X, Tao Y, Lamas V, Huang M, Yeh WH, Pan B, et al. Treatment of autosomal dominant hearing loss by in vivo delivery of genome editing agents. Nature. 2018;553(7687):217–21.

620. Chambers S, Newbold C, Stathopoulos D, Needham K, Miller C, Risi F, et al. Protecting against electrode insertion trauma using dexamethasone. Cochlear Implants Int. 2018;1–11.

621. Schulze J, Sasse S, Prenzler N, Staecker H, Mellott AJ, Roemer A, et al. Microenvironmental support for cell delivery to the inner ear. Hear Res. 2018;

622. Ayoob AM, Peppi M, Tandon V, Langer R, Borenstein JT. A fluorescence-based imaging approach to pharmacokinetic analysis of intracochlear drug delivery. Hear Res. 2018;

623. Fransson A, Tornoe J, Wahlberg LU, Ulfendahl M. The feasibility of an encapsulated cell approach in an animal deafness model. J Control Release. 2018;270:275–81.

624. Gyorgy B, Meijer E, Indzhykulian A, Volak B, Leroy S, Maguire C. A single neonatal injection of PHP.B-AAV9-CLRN1 rescues hearing in a model of usher syndrome type IIIA. Mol Ther. 2018;26(5 Supplement 1):170–1.

625. Akil O, Blits B, Lustig LR, Leake PA. Virally Mediated Overexpression of Glial-Derived Neurotrophic Factor Elicits Age- and Dose-Dependent Neuronal Toxicity and Hearing Loss. Hum Gene Ther. 2018;

626. Peyvandi AA, Abbaszadeh H-A, Roozbahany NA, Pourbakht A, Khoshsirat S, Niri HH, et al. Deferoxamine promotes mesenchymal stem cell homing in noise-induced injured cochlea through PI3K/AKT pathway. Cell Prolif. 2018;51(2):e12434.

627. Chen J, Hong F, Zhang C, Li L, Wang C, Shi H, et al. Differentiation and transplantation of human induced pluripotent stem cell-derived otic epithelial progenitors in mouse cochlea. Stem Cell Res Ther. 2018;9(1):230.

628. Prenzler NK, Salcher R, Timm M, Gaertner L, Lenarz T, Warnecke A, et al. Intracochlear administration of steroids with a catheter during human cochlear implantation: a safety and feasibility study. Drug Deliv Transl Res. 2018;8(5):1191–9.

629. Dulon D, Papal S, Patni P, Cortese M, Vincent PF, Tertrais M, et al. Clarin-1 gene transfer rescues auditory synaptopathy in model of Usher syndrome. J Clin Invest. 2018;128(8):3382–401.

630. Pierstorff E, Chen S, Chaparro MP, Cortez JMJ, Chen Y-J, Ryu SY, et al. A Polymer-Based Extended Release System for Stable, Long-term Intracochlear Drug Delivery. Otol Neurotol. 2018;39(9):1195–202.

631. Wrobel C, Dieter A, Huet A, Keppeler D, Duque-Afonso C, Vogl C, et al. Optogenetic stimulation of cochlear neurons activates the auditory pathway and restores auditory-driven behavior in deaf adult gerbils. Sci Transl Med. 2018;10(449):eaao0540.

632. Creber NJ, Eastwood HT, Hampson AJ, Tan J, O’leary SJ. A comparison of cochlear distribution and glucocorticoid receptor activation in local and systemic dexamethasone drug delivery regimes. 2018;

633. Dormer NH, Nelson-Brantley J, Staecker H, Berkland CJ. Evaluation of a transtympanic delivery system in Mus musculus for extended release steroids A R T I C L E I N F O. 2018;

634. Zanetti D, Di Berardino F, Nassif N. Intratympanic steroid delivery by an indwelling catheter in refractory severe sudden sensorineural hearing loss. Auris Nasus Larynx. 2018;45(2):227–33.

635. Mamelle E, El Kechai N, Adenis V, Nguyen Y, Sterkers O, Agnely F, et al. Assessment of the efficacy of a local steroid rescue treatment administered 2 days after a moderate noise-induced trauma in guinea pig. Acta Otolaryngol. 2018;138(7):610–6.

636. Dai J, Long W, Liang Z, Wen L, Yang F, Chen G. A novel vehicle for local protein delivery to the inner ear: injectable and biodegradable thermosensitive hydrogel loaded with PLGA nanoparticles. Drug Dev Ind Pharm. 2018;44(1):89–98.

637. Creber NJ, Eastwood HT, Hampson AJ, Tan J, O’Leary SJ, N.J. C, et al. Adjuvant agents enhance round window membrane permeability to dexamethasone and modulate basal to apical cochlear gradients. Eur J Pharm Sci. 2018;

638. Kayyali MN, Wooltorton JRA, Ramsey AJ, Lin M, Chao TN, Tsourkas A, et al. A novel nanoparticle delivery system for targeted therapy of noise-induced hearing loss. J Control Release. 2018;279:243–50.

639. Yang K-J, Son J, Jung SY, Yi G, Yoo J, Kim D-K, et al. Optimized phospholipid-based nanoparticles for inner ear drug delivery and therapy. Biomaterials. 2018;171:133–43.

640. Martin-Saldana S, Palao-Suay R, Aguilar MR, Garcia-Fernandez L, Arevalo H, Trinidad A, et al. pH-sensitive polymeric nanoparticles with antioxidant and anti-inflammatory properties against cisplatin-induced hearing loss. J Control Release. 2018;270:53–64.

641. Li W, Hartsock JJ, Dai C, Salt AN. Permeation Enhancers for Intratympanically-applied Drugs Studied Using Fluorescent Dexamethasone as a Marker. Otol Neurotol. 2018;39(5):639–47.

642. Kurihara S, Fujioka M, Yoshida T, Koizumi M, Ogawa K, Kojima H, et al. A Surgical Procedure for the Administration of Drugs to the Inner Ear in a Non-Human Primate Common Marmoset (Callithrix jacchus). J Vis Exp. 2018;(132).

643. Cer, Vantes B, Arana L, Murillo-Cuesta S, Bruno M, Alkorta I. Glucocorticoids combined with solid lipid nanovesicles efficiently protect otic cells from damage. FEBS Open Bio. 2018;8(Supplement 1):236.

644. Shimoji M, Ramaswamy B, Shukoor MI, Benhal P, Broda A, Kulkarni S, et al. Toxicology study for magnetic injection of prednisolone into the rat cochlea. Eur J Pharm Sci. 2018;

645. Ding S, Xie S, Chen W, Wen L, Wang J, Yang F, et al. Is oval window transport a royal gate for nanoparticle delivery to vestibule in the inner ear? Eur J Pharm Sci. 2018;

646. Zhang L, Xu Y, Cao W, Xie S, Wen L, Chen G. Understanding the translocation mechanism of PLGA nanoparticles across round window membrane into the inner ear: A guideline for inner ear drug delivery based on nanomedicine. Int J Nanomedicine. 2018;13:479–92.

647. Buki B, Junger H. Intratympanal gentamicin in Meniere’s disease: Effects on individual semicircular canals. Auris Nasus Larynx. 2018;45(1):170–4.

648. Amiraraghi N, Gaggini M, Crowther J, Locke R, Hastings L, Taylor W. Prehabilitation with intratympanic gentamycin in vestibular schwannoma patients and postoperative contralateral benefits: A prospective study. J Neurol Surgery, Part B Skull Base. 2018;79(Supplement 1).

649. Wu Q, Zhang Y, Dai C, Kong Y. The Degeneration of the Vestibular Efferent Neurons After Intratympanic Gentamicin Administration. J Histochem Cytochem. 2018;66(1):801–12.

650. Fetoni AR, Eramo SLM, Di Pino A, Rolesi R, Paciello F, Grassi C, et al. The Antioxidant Effect of Rosmarinic Acid by Different Delivery Routes in the Animal Model of Noise-Induced Hearing Loss. Otol Neurotol. 2018;39(3):378–86.

651. Wang Y, Han L, Diao T, Jing Y, Wang L, Zheng H, et al. A comparison of systemic and local dexamethasone administration: From perilymph/cochlea concentration to cochlear distribution. Hear Res. 2018;370:1–10.

652. Lee H-KH-J, Kim M-B, Yoo S-Y, Park SN, Nam E-C, Moon IS, et al. Clinical effect of intratympanic dexamethasone injection in acute unilateral tinnitus: A prospective, placebo-controlled, multicenter study. Laryngoscope. 2018;128(1):184–8.

653. Suzuki H, Wakasugi T, Kitamura T, Koizumi H, Do BH, Ohbuchi T. Comparison of 2 and 4 Intratympanic Steroid Injections in the Treatment of Idiopathic Sudden Sensorineural Hearing Loss. Ann Otol Rhinol Laryngol. 2018;127(4):235–40.

654. Sun H, Qiu X, Hu J. Comparison of intratympanic dexamethasone therapy and hyperbaric oxygen therapy for the salvage treatment of refractory high-frequency sudden sensorineural hearing loss. Am J Otolaryngol - Head Neck Med Surg. 2018;39(5):531–5.

655. Demirhan H, Gokduman A, Hamit B, Yurekli Altindag M. Contribution of intratympanic steroids in the primary treatment of sudden hearing loss*. Acta Otolaryngol. 2018;138(7):648–51.

656. Park J, Kim S, Kim M. Effect of intratympanic steroid injection in light cupula. Acta Otolaryngol. 2018;138(9):769–74.

657. Gumrukcu SS, Topaloglu I, Salturk Z, Tutar B, Atar Y, Berkiten G, et al. Effects of intratympanic dexamethasone on noise-induced hearing loss: An experimental study. Am J Otolaryngol. 2018;39(1):71–3.

658. Tas A, Bulut E, Tas M, Yagiz R, Turan P, Huseyinoglu A. Effects of intratympanic steroid on cisplatin ototoxicity: An electrophysiological and ultrastructural study. UHOD - Uluslararasi Hematol Derg. 2018;28(2):104–11.

659. Ashtiani MK, Firouzi F, Bastaninejad S, Dabiri S, Nasirmohtaram S, Saeedi N, et al. Efficacy of systemic and intratympanic corticosteroid combination therapy versus intratympanic or systemic therapy in patients with idiopathic sudden sensorineural hearing loss: a randomized controlled trial. Eur Arch Otorhinolaryngol. 2018;275(1):89–97.

660. Zhu C, Gausterer JC, Schopper H, Nieratschker M, Saidov N, Ahmadi N, et al. Evaluation of Sustained-Release Steroid Hydrogels in a Guinea Pig Model for Noise-Induced Hearing Loss. Audiol Neurootol. 2018;23(2):73–81.

661. Morgan A, Ismail E. Intratympanic Injections of Dexamethasone in Delayed Endolymphatic Hydrops: A Prospective Clinical Study. ORL. 2018;80(1):19–27.

662. Gulustan F, Yazici Z, Alakhras W, Erdur O, Acipayam H, Kufeciler L. Intratympanic steroid injection and hyperbaric oxygen therapy for the treatment of refractory sudden hearing loss. Braz J Otorhinolaryngol. 2018;84(1):28–33.

663. Hara J, Zhang J, Gandhi K, Flaherty A, Barber W, Leung M, et al. Oral and intratympanic steroid therapy for idiopathic sudden sensorineural hearing loss. Laryngoscope Investig Otolaryngol. 2018;3(2):73–7.

664. Lee JJ, Jang JH, Choo O-S, Lim HJ, Choung Y-H. Steroid intracochlear distribution differs by administration method: Systemic versus intratympanic injection. Laryngoscope. 2018;128(1):189–94.

665. Tsounis M, Psillas G, Tsalighopoulos M, Vital V, Maroudias N, Markou K. Systemic, intratympanic and combined administration of steroids for sudden hearing loss. A prospective randomized multicenter trial. Eur Arch Otorhinolaryngol. 2018;275(1):103–10.

666. Topcuoglu T, Kocyigit M, Bulut E, Ortekin SG, Kanter M, Yagiz R, et al. The effects of experimental intratympanic steroid administration on organ of corti type 1 spiral ganglion. Int Arch Otorhinolaryngol. 2018;22(2):171–6.

667. Nasr W, Abdelhady M, Abd Elbary M. Treatment of cisplatin-induced ototoxicity by intra-tympanic corticosteroid injection. Indian J Otol. 2018;24(1):33–7.

668. Teitz T, Fang J, Goktug AN, Bonga JD, Diao S, Hazlitt RA, et al. CDK2 inhibitors as candidate therapeutics for cisplatin- and noise-induced hearing loss. J Exp Med. 2018 Apr 2;215(4):1187–203.

669. Dogan R, Eren SB, Gedik O, Yenigun A, Aydin MS, Eser B, et al. Different concentrations of mesna application have an effect on the internal ear?. Int J Pediatr Otorhinolaryngol. 2018;109:31–5.

670. Lobo D, Tunon M, Villarreal I, Brea B, Garcia-Berrocal JR, D. L, et al. Intratympanic gadolinium magnetic resonance imaging supports the role of endolymphatic hydrops in the pathogenesis of immune-mediated inner-ear disease. J Laryngol Otol. 2018;132(6):554–9.

671. Salihoglu M, Dogru S, Cesmeci E, Caliskan H, Kurt O, Kucukodaci Z, et al. Ototoxicity of boric acid powder in a rat animal model. Braz J Otorhinolaryngol. 2018;84(3):332–7.

672. Schroeder R, Audlin J, Luo J. Pharmacokinetics of sodium thiosulfate in Guinea pig perilymph following middle ear application. J Otol. 2018;13(2):54–8.

673. Naples J, Cox R, Bonaiuto G, Parham K, J. N, R. C, et al. Prestin as an Otologic Biomarker of Cisplatin Ototoxicity in a Guinea Pig Model. Otolaryngol - Head Neck Surg (United States). 2018;158(3):541–6.

674. Ghosh S, Sheth S, Sheehan K, Mukherjea D, Dhukhwa A, Borse V, et al. The endocannabinoid/cannabinoid receptor 2 system protects against cisplatin-induced hearing loss. Front Cell Neurosci. 2018;12:271.

675. Arslan IB, Aslan GG, Mercan GC, Vatansever S, Cukurova I, Gokalp S, et al. Therapeutic and protective effects of autologous serum in amikacin-induced ototoxicity. J Laryngol Otol. 2018;132(1):33–40.

676. Sheehan K, Sheth S, Mukherjea D, Rybak LP, Ramkumar V, K. S, et al. Trans-Tympanic Drug Delivery for the Treatment of Ototoxicity. J Vis Exp. 2018;(133).

677. Guo J-Y, He L, Qu T-F, Liu Y-Y, Liu K, Wang G-P, et al. Canalostomy As a Surgical Approach to Local Drug Delivery into the Inner Ears of Adult and Neonatal Mice. J Vis Exp. 2018;(135).

678. Tao Y, Huang M, Shu Y, Ruprecht A, Wang H, Tang Y, et al. Delivery of Adeno-Associated Virus Vectors in Adult Mammalian Inner-Ear Cell Subtypes Without Auditory Dysfunction. Hum Gene Ther. 2018 Apr 1;29(4):492–506.

679. Isgrig K, Chien WW. Posterior Semicircular Canal Approach for Inner Ear Gene Delivery in Neonatal Mouse. J Vis Exp. 2018;(133).

Reviews:

1. McCall AA, Swan EEL, Borenstein JT, Sewell WF, Kujawa SG, McKenna MJ. Drug delivery for treatment of inner ear disease: current state of knowledge. Ear Hear. 2010;31(2):156–65.

2. Banerjee A, Parnes LS. The biology of intratympanic drug administration and pharmacodynamics of round window drug absorption. Otolaryngol Clin North Am. 2004;37(5):1035–51.

3. Chuang-Chuang A, Baeza MA. Are intratympanic corticosteroids effective for Menieres disease?. ?Son Ef los corticoides intratimpanicos en la Enferm Meniere? 2017;17(Supplement1):e6863.

4. Luebke AE, Rova C, Von Doersten PG, Poulsen DJ. Adenoviral and AAV-mediated gene transfer to the inner ear: role of serotype, promoter, and viral load on in vivo and in vitro infection efficiencies. Adv Otorhinolaryngol. 2009;66:87–98.

5. Ahmed H, Shubina-Oleinik O, Holt JR. Emerging Gene Therapies for Genetic Hearing Loss. J Assoc Res Otolaryngol. 2017;18(5):649–70.

6. Hu A, Parnes LS. Intratympanic Steroids for Inner Ear Disorders: A Review. Audiol Neurotol. 2009;14:373–82.

7. Lalwani AK, Mhatre AN. Cochlear gene therapy. Ear Hear. 2003;24(4):342–8.

8. Lalwani AK, Mhatre AN. Cochlear Gene Therapy. Adv Otorhinolaryngol Basel, Karger. 2000;56:275–8.

9. Lalwani AK, Jero J, Mhatre AN. Developments in Cochlear Gene Therapy. Adv Otorhinolaryngol Basel, Karger. 2002;61:28–33.

10. Lalwani AK, Jero J, Mhatre AN. Current issues in cochlear gene transfer. Audiol Neurootol. 2002;7(3):146–51.

11. Wise AK, Gillespie LN. Drug delivery to the inner ear. J Neural Eng. 2012;9(6):65002.

12. Liebau A, Pogorzelski O, Salt AN, Plontke SK. Hearing Changes After Intratympanic Steroids for Secondary (Salvage) Therapy of Sudden Hearing Loss: A Meta-Analysis Using Mathematical Simulations of Drug Delivery Protocols. Otol Neurotol. 2018;39(7):803–15.

13. Liebau A, Pogorzelski O, Salt AN, Plontke SK, A. L, O. P, et al. Hearing Changes After Intratympanically Applied Steroids for Primary Therapy of Sudden Hearing Loss: A Meta-analysis Using Mathematical Simulations of Drug Delivery Protocols. Otol Neurotol. 2017;38(1):19–30.

14. Salt AN, Plontke SK. Pharmacokinetic principles in the inner ear: Influence of drug properties on intratympanic applications. Hear Res. 2018;368:28–40.

15. Salt AN, Plontke SK. Principles of local drug delivery to the inner ear. Audiol Neurootol. 2009;14(6):350–60.

16. Salt AN, Plontke SKR. Local inner-ear drug delivery and pharmacokinetics. Drug Discov Today. 2005 Jan 26;10(19):1299–306.

17. Salt AN, Hirose K. Communication pathways to and from the inner ear and their contributions to drug delivery. Hear Res. 2018;362:25–37.

18. Straube A. Pharmacology of vertigo/nystagmus/oscillopsia. Curr Opin Neurol. 2005;18(1):11–4.

19. Blakley BW. Update on intratympanic gentamicin for Meniere’s disease. Laryngoscope. 2000;110(2 Pt 1):236–40.

20. Kesser BW, Hashisaki GT, Holt JR. Gene transfer in human vestibular epithelia and the prospects for inner ear gene therapy. Laryngoscope. 2008;118(5):821–31.

21. Choo DI, Tawfik KO, Martin DM, Raphael Y. Inner ear manifestations in CHARGE: Abnormalities, treatments, animal models, and progress toward treatments in auditory and vestibular structures. Am J Med Genet Part C Semin Med Genet. 2017 Dec 1;175(4):439–49.

22. Diamond C, O’Connell DA, Hornig JD, Liu R. Systematic review of intratympanic gentamicin in Meniere’s disease. J Otolaryngol. 2003 Dec;32(6):351–61.

23. Hobson CE, Alexander TH, Harris JP. Primary treatment of idiopathic sudden sensorineural hearing loss with intratympanic dexamethasone. Curr Opin Otolaryngol Head Neck Surg. 2016;24(5):407–12.

24. Garnham C, Reetz G, Jolly C, Miller J, Salt A, Beal F. Drug delivery to the cochlea after implantation: Consideration of the risk factors. Cochlear Implants Int. 2005;6(SUPPL. 1):12–4.

25. Jolly C, Garnham C, Mirzadeh H, Truy E, Martini A, Kiefer J, et al. Electrode features for hearing preservation and drug delivery strategies. Adv Otorhinolaryngol. 2010;67:28–42.

26. Darlington CL, Smith PF. Drug treatments for tinnitus. Prog Brain Res. 2007;166:249–62.

27. Cunningham L. Hearing loss in adults. N Engl J Med. 2017;377(25):2465–73.

28. Maiorana CR, Staecker H. Advances in inner ear gene therapy: exploring cochlear protection and regeneration. Curr Opin Otolaryngol Head Neck Surg. 2005;13(5):308–12.

29. Borkholder DA. State-of-the-art mechanisms of intracochlear drug delivery. Curr Opin Otolaryngol Head Neck Surg. 2008;16(5):472–7.

30. Assimakopoulos D, Patrikakos G. Treatment of Meniere’s disease by intratympanic gentamicin application. J Laryngol Otol. 2003;117(1):10–6.

31. Bagger-Sjoback D. Effect of streptomycin and gentamicin on the inner ear. Ann N Y Acad Sci. 1997;830:120–9.

32. Lai D, Zhao F, Jalal N, Zheng Y. Intratympanic glucocorticosteroid therapy for idiopathic sudden hearing loss: Meta-analysis of randomized controlled trials. Med (United States). 2017;96(50):e8955.

33. Barrs DM. Intratympanic corticosteroids for Meniere’s disease and vertigo. Otolaryngol Clin North Am. 2004;37(5):955–v.

34. Martin DM, Raphael Y. Gene-based diagnostic and treatment methods for tinnitus. Int Tinnitus J. 2003;9(1):3–10.

35. Schoo DP, Tan GX, Ehrenburg MR, Pross SE, Ward BK, Carey JP. Intratympanic (IT) Therapies for Meniere’s Disease: Some Consensus Among the Confusion. Curr Otorhinolaryngol Rep. 2017;5(2):132–41.

36. Poe DS, Pyykko I. Nanotechnology and the treatment of inner ear diseases. Wiley Interdiscip Rev Nanomed Nanobiotechnol. 2011;3(2):212–21.

37. Kim D-K. Nanomedicine for Inner Ear Diseases: A Review of Recent In Vivo Studies. Biomed Res Int. 2017;2017:3098230.

38. Swan EEL, Mescher MJ, Sewell WF, Tao SL, Borenstein JT. Inner ear drug delivery for auditory applications. Adv Drug Deliv Rev. 2008 Jan 26;60(15):1583–99.

39. Monsell EM, Cass SP, Rybak LP. Therapeutic use of aminoglycosides in Meniere’s disease. Otolaryngol Clin North Am. 1993;26(5):737–46.

40. Chirtes F, Albu S. An overview of pharmacology and clinical aspects concerning the therapy of cochleo-vestibular syndromes by intratympanic drug delivery. Clujul Med. 2013;86(3):185–91.

41. Fazel M, Jedlowski P, Cravens R, Erstad B. Evaluation and Treatment of Acute and Subacute Hearing Loss: A Review of Pharmacotherapy. Pharmacotherapy. 2017;37(12):1600–16.

42. Mohammadian F, Eatemadi A, Daraee H. Inner ear drug delivery using liposomes. Cell Mol Biol (Noisy-le-grand). 2017;63(1):28–33.

43. Brookes GB. The pharmacological treatment of Meniere’s disease. Clin Otolaryngol Allied Sci. 1996;21(1):3–11.

44. Chen G, Zhang X, Yang F, Mu L. Disposition of nanoparticle-based delivery system via inner ear administration. Curr Drug Metab. 2010;11(10):886–97.

45. Li H, Feng G, Wang H, Feng Y. Intratympanic Steroid Therapy as a Salvage Treatment for Sudden Sensorineural Hearing Loss After Failure of Conventional Therapy: A Meta-analysis of Randomized, Controlled Trials. Clin Ther. 2014;37(1):178–87.

46. Staecker H, Rodgers B. Developments in delivery of medications for inner ear disease. Expert Opin Drug Deliv. 2013 Oct 8;10(5):639–50.

47. Staecker H, Brough DE, Praetorius M, Baker K. Drug delivery to the inner ear using gene therapy. Otolaryngol Clin North Am. 2004;37(5):1091–108.

48. Sun H, Huang A, Cao S. Current status and prospects of gene therapy for the inner ear. Hum Gene Ther. 2011;22(11):1311–22.

49. Takeda H, Dondzillo A, Randall JA, Gubbels SP. Challenges in Cell-Based Therapies for the Treatment of Hearing Loss. Trends Neurosci. 2018;

50. Grant IL, Welling DB. The treatment of hearing loss in Meniere’s disease. Otolaryngol Clin North Am. 1997;30(6):1123–44.

51. Pyykkö I, Zou J, Schrott-Fischer A, Glueckert R, Kinnunen P. An Overview of Nanoparticle Based Delivery for Treatment of Inner Ear Disorders. In Humana Press, New York, NY; 2016. p. 363–415.

52. Pyykko I, Zou J, Zhang W, Zhang Y. Nanoparticle-based delivery for the treatment of inner ear disorders. Curr Opin Otolaryngol Head Neck Surg. 2011;19(5):388–96.

53. Seggas I, Koltsidopoulos P, Bibas A, Tzonou A, Sismanis A. Intratympanic steroid therapy for sudden hearing loss: a review of the literature. Otol Neurotol. 2011;32(1):29–35.

54. Carey J. Intratympanic gentamicin for the treatment of Meniere’s disease and other forms of peripheral vertigo. Otolaryngol Clin North Am. 2004;37(5):1075–90.

55. Claes J, Van de Heyning PH. A review of medical treatment for Meniere’s disease. Acta Otolaryngol Suppl. 2000;544:34–9.

56. Sharon JD, Trevino C, Schubert MC, Carey JP. Treatment of Meniere’s Disease. Curr Treat Options Neurol. 2015;17(4):341.

57. Devare J, Gubbels S, Raphael Y, J. D, S. G, Y R. Outlook and future of inner ear therapy. Hear Res. 2018;

58. Borenstein JT. Intracochlear drug delivery systems. Expert Opin Drug Deliv. 2011;8(9):1161–74.

59. Ng JH, Ho RCM, Cheong CSJ, Ng A, Yuen HW, Ngo RYS. Intratympanic steroids as a salvage treatment for sudden sensorineural hearing loss? A meta-analysis. Eur Arch Otorhinolaryngol. 2015;272(10):2777–82.

60. Hao J, Li SK. Inner ear drug delivery: Recent advances, challenges, and perspective. Eur J Pharm Sci. 2018;

61. Kuthubutheen J, Smith L, Hwang E, Lin V. Preoperative steroids for hearing preservation cochlear implantation: A review. Cochlear Implants Int. 2016;17(2):63–74.

62. Hendricks JL, Chikar JA, Crumling MA, Raphael Y, Martin DC. Localized cell and drug delivery for auditory prostheses. Hear Res. 2008;242(1–2):117–31.

63. Nevoux J, Barbara M, Dornhoffer J, Gibson W, Kitahara T, Darrouzet V. International consensus (ICON) on treatment of Meniere’s disease. Eur Ann Otorhinolaryngol Head Neck Dis. 2018;135(1 Supplement):S29–32.

64. Nevoux J, Franco-Vidal V, Bouccara D, Parietti-Winkler C, Uziel A, Chays A, et al. Diagnostic and therapeutic strategy in Meniere’s disease. Guidelines of the French Otorhinolaryngology-Head and Neck Surgery Society (SFORL). Eur Ann Otorhinolaryngol Head Neck Dis. 2017;134(6):441–4.

65. Light JP, Silverstein H. Transtympanic perfusion: indications and limitations. Curr Opin Otolaryngol Head Neck Surg. 2004;12(5):378–83.

66. Phillips J, Westerberg B. Intratympanic steroids for Meniere’s disease or syndrome. Cochrane Database Syst Rev. 2011;(7):CD008514.

67. Wang J, Puel J-L. Toward Cochlear Therapies. Physiol Rev. 2018;98(4):2477–522.

68. Doyle KJ, Bauch C, Battista R, Beatty C, Hughes GB, Mason J, et al. Intratympanic steroid treatment: a review. Otol Neurotol. 2004;25(6):1034–9.

69. Hoffmann KK, Silverstein H. Inner ear perfusion: indications and applications. Curr Opin Otolaryngol Head Neck Surg. 2003;11(5):334–9.

70. Dodson KM, Sismanis A. Intratympanic perfusion for the treatment of tinnitus.

71. Mader K, Lehner E, Liebau A, Plontke SK, K. M, E. L, et al. Controlled drug release to the inner ear: Concepts, materials, mechanisms, and performance. Hear Res. 2018;

72. Nguyen K, Kempfle JS, Jung DH, McKenna CE, K. N, J.S. K, et al. Recent advances in therapeutics and drug delivery for the treatment of inner ear diseases: a patent review (2011-2015). Expert Opin Ther Pat. 2017;27(2):191–202.

73. Jackson LE, Silverstein H. Chemical perfusion of the inner ear. Otolaryngol Clin North Am. 2002;35(3):639–53.

74. Li L, Chao T, Brant J, O’Malley BJ, Tsourkas A, Li D, et al. Advances in nano-based inner ear delivery systems for the treatment of sensorineural hearing loss. Adv Drug Deliv Rev. 2017;108:2–12.

75. Bianchi LM, Raz Y. Methods for providing therapeutic agents to treat damaged spiral ganglion neurons. Curr Drug Targets CNS Neurol Disord. 2004;3(3):195–9.

76. Odkvist LM, Bergenius J, Möller C. When and how to use gentamicin in the treatment of Menière’s disease. Acta Otolaryngol Suppl. 1997;526(November):54–7.

77. Lustig LR, Akil O. Cochlear gene therapy. Curr Opin Neurol. 2012;25(1):57–60.

78. Wang L, Kempton JB, Brigande J V. Gene Therapy in Mouse Models of Deafness and Balance Dysfunction. Front Mol Neurosci. 2018;11:300.

79. Huon L-K, Fang T-Y, Wang P-C. Outcomes of intratympanic gentamicin injection to treat Meniere’s disease. Otol Neurotol. 2012;33(5):706–14.

80. Barreto MA de SC, Ledesma ALL, de Oliveira CACP, Bahmad FJ. Intratympanic corticosteroid for sudden hearing loss: does it really work?. Braz J Otorhinolaryngol. 2016;82(3):353–64.

81. Parker MA. Biotechnology in the Treatment of Sensorineural Hearing Loss: Foundations and Future of Hair Cell Regeneration. J Speech Lang Hear Res. 2011;54(6):1709.

82. Holley MC. Application of new biological approaches to stimulate sensory repair and protection. Br Med Bull. 2002;63:157–69.

83. Seidman MD, Vivek P. Intratympanic treatment of hearing loss with novel and traditional agents. Otolaryngol Clin North Am. 2004;37(5):973–90.

84. Seidman MD, Van De Water TR. Pharmacologic manipulation of the labyrinth with novel and traditional agents delivered to the inner ear. Ear Nose Throat J. 2003;82(4):276–passim.

85. Diensthuber M, Stover T. Strategies for a regenerative therapy of hearing loss. Strateg fur eine Regen Ther der Schwerhorigkeit. 2018;66(Suppl 1):39–46.

86. Duan M, Venail F, Spencer N, Mezzina M. Treatment of peripheral sensorineural hearing loss: gene therapy. Gene Ther. 2004;11 Suppl 1:S51-6.

87. Hoffer ME, Balough BJ, Gottshall KR. Delivery of drugs to the inner ear. Curr Opin Otolaryngol Head Neck Surg. 2006;14(5):329–31.

88. Hoffer ME, Balough BJ, Gottshall KR, Allen K, Weisskopf P, Wester D, et al. Sustained-release devices in inner ear medical therapy. Otolaryngol Clin North Am. 2004;37(5):1053–60.

89. Crowson MG, Hertzano R, Tucci DL. Emerging Therapies for Sensorineural Hearing Loss. Otol Neurotol. 2017;

90. Hamid M, Trune D. Issues, indications, and controversies regarding intratympanic steroid perfusion. Curr Opin Otolaryngol Head Neck Surg. 2008;16(5):434–40.

91. Syed MI, Ilan O, Nassar J, Rutka JA. Intratympanic therapy in Meniere’s syndrome or disease: up to date evidence for clinical practice. Clin Otolaryngol. 2015;40(6):682–90.

92. Alles MJRC, der Gaag MA, Stokroos RJ. Intratympanic steroid therapy for inner ear diseases, a review of the literature. Eur Arch Otorhinolaryngol. 2006;263(9):791–7.

93. Wareing MJ, Lalwani AK. Cochlear gene therapy: current perspectives. Int J Pediatr Otorhinolaryngol. 1999;49 Suppl 1:S27-30.

94. Schwaber MK. Transtympanic gentamicin perfusion for the treatment of Meniere’s disease. Otolaryngol Clin North Am. 2002;35(2):287–vi.

95. Rivolta MN. New strategies for the restoration of hearing loss: challenges and opportunities. Br Med Bull. 2013;105:69–84.

96. Marques P, Dias C, Perez-Fernandez N. Instrumental head impulse test changes after intratympanic gentamicin for unilateral definite Meniere’s disease: A systematic review and meta-analysis. Auris Nasus Larynx. 2018;45(5):943–51.

97. Peppi, M, Marie, A, Belline, C, Borenstein J. Intracochlear drug delivery systems: a novel approach whose time has come. Expert Opin Drug Deliv. 2018;15:319–24.

98. Ralli M, Rolesi R, Anzivino R, Turchetta R, Fetoni AR. Acquired sensorineural hearing loss in children: current research and therapeutic perspectives. Sordita Infant acquisita stato dell’arte della Ric e Prospett Ter. 2017;37(6):500–8.

99. Strupp M, Brandt T. Pharmacological advances in the treatment of neuro-otological and eye movement disorders. Curr Opin Neurol. 2006;19(1):33–40.

100. Suckfull M. Perspectives on the pathophysiology and treatment of sudden idiopathic sensorineural hearing loss. Dtsch Arztebl Int. 2009;106(41):669–76.

101. Lee MY, Park Y-H. Potential of Gene and Cell Therapy for Inner Ear Hair Cells. Biomed Res Int. 2018;2018:8137614.

102. El Kechai N, Agnely F, Mamelle E, Nguyen Y, Ferrary E, Bochot A. Recent advances in local drug delivery to the inner ear. Int J Pharm. 2015 Dec 5;494(1):83–101.

103. El Sabbagh NG, Sewitch MJ, Bezdjian A, Daniel SJ. Intratympanic dexamethasone in sudden sensorineural hearing loss: A systematic review and meta-analysis. Laryngoscope. 2017;127(8):1897–908.

104. Jongkamonwiwat N, Zine A, Rivolta MN. Stem cell based therapy in the inner ear: appropriate donor cell types and routes for transplantation. Curr Drug Targets. 2010;11(7):888–97.

105. Patel NP, Mhatre AN, Lalwani AK. Biological therapy for the inner ear. Expert Opin Biol Ther. 2004;4(11):1811–9.

106. Yamamoto N, Nakagawa T, Ito J. Application of insulin-like growth factor-1 in the treatment of inner ear disorders. Front Pharmacol. 2014;5:208.

107. Bird PA, Bergin MJ. Pharmacological Issues in Hearing Rehabilitation. Adv Otorhinolaryngol. 2018;81:114–22.

108. Smith PF, Darlington CL. Drug treatments for subjective tinnitus: serendipitous discovery versus rational drug design. Curr Opin Investig Drugs. 2005;6(7):712–6.

109. Lavigne P, Lavigne F, Saliba I. Intratympanic corticosteroids injections: a systematic review of literature. Eur Arch Otorhinolaryngol. 2016;273(9):2271–8.

110. Lefebvre PP, Staecker H, Van De Water T, Moonen G, Malgrange B. Microperfusion of the inner ear. Acta Otorhinolaryngol Belg. 2002;56(4):365–8.

111. Lefebvre PP, Staecker H, Van de Water T, Moonen G, Malgrange B. Pharmacologic treatment of inner ear: from basic science to the patient. Acta Otorhinolaryngol Belg. 2002;56(1):45–9.

112. Vlastarakos P V, Iacovou E, Nikolopoulos TP. Is gentamycin delivery via sustained-release vehicles a safe and effective treatment for refractory Meniere’s disease? A critical analysis of published interventional studies. Eur Arch Oto-Rhino-Laryngology. 2017 Mar 3;274(3):1309–15.

113. Vlastarakos P V, Papacharalampous G, Maragoudakis P, Kampessis G, Maroudias N, Candiloros D, et al. Are intra-tympanically administered steroids effective in patients with sudden deafness? Implications for current clinical practice. Eur Arch Otorhinolaryngol. 2012;269(2):363–80.

114. Qiang Q, Wu X, Yang T, Yang C, Sun H, Q. Q, et al. A comparison between systemic and intratympanic steroid therapies as initial therapy for idiopathic sudden sensorineural hearing loss: a meta-analysis. Acta Otolaryngol. 2017;137(6):598–605.

115. Crane RA, Camilon M, Nguyen S, Meyer TA. Steroids for treatment of sudden sensorineural hearing loss: a meta-analysis of randomized controlled trials. Laryngoscope. 2015;125(1):209–17.

116. Cohen-Kerem R, Kisilevsky V, Einarson TR, Kozer E, Koren G, Rutka JA. Intratympanic gentamicin for Meniere’s disease: a meta-analysis. Laryngoscope. 2004;114(12):2085–91.

117. Frisina RD, Budzevich M, Zhu X, Martinez G V, Walton JP, Borkholder DA. Animal model studies yield translational solutions for cochlear drug delivery. Hear Res. 2018;

118. Rudman J, Mei C, Bressler S, Blanton S. Precision medicine in hearing loss. J Genet Genomics. 2018;45(2):99–109.

119. Kopke R, Staecker H, Lefebvre P, Malgrange B, Moonen G, Ruben RJ, et al. Effect of neurotrophic factors on the inner ear: clinical implications. Acta Otolaryngol. 1996;116(2):248–52.

120. Mittal R, Nguyen D, Patel AP, Debs LH, Mittal J, Yan D, et al. Recent advancements in the regeneration of auditory hair cells and hearing restoration. Front Mol Neurosci. 2017;10:236.

121. Mittal R, Jung HD, Mittal J, Eshraghi AA, R. M, H.D. J, et al. A perspective on stem cell therapy for ear disorders. J Cell Physiol. 2018;233(3):1823–4.

122. Ruan RS, Soh KB, Yeoh KH. What you need to know--hearing loss and inner ear diseases--can they be cured?. Singapore Med J. 1999;40(1):60–1.

123. Sacheli R, Delacroix L, Vandenackerveken P, Nguyen L, Malgrange B. Gene transfer in inner ear cells: a challenging race. Gene Ther. 2013;20(3):237–47.

124. Richardson, Rachael, Atkinson P. Atoh1 gene therapy in the cochlea for hair cell regeneration. Expert Opin Biol Ther. 2015;15(3):417–30.

125. Richardson RT, Wise AK, Andrew JK, O’Leary SJ. Novel drug delivery systems for inner ear protection and regeneration after hearing loss. Expert Opin Drug Deliv. 2008;5(10):1059–76.

126. Richardson RT, Noushi F, O’Leary S. Inner ear therapy for neural preservation. Audiol Neurootol. 2006;11(6):343–56.

127. Spear SA, Schwartz SR. Intratympanic steroids for sudden sensorineural hearing loss: a systematic review. Otolaryngol Head Neck Surg. 2011;145(4):534–43.

128. Barriat S, Poirrier A, Malgrange B, Lefebvre P. Hearing preservation in cochlear implantation and drug treatment. Adv Otorhinolaryngol. 2010;67:6–13.

129. Rauch SD. Intratympanic steroids for sensorineural hearing loss. Otolaryngol Clin North Am. 2004;37(5):1061–74.

130. Chia SH, Gamst AC, Anderson JP, Harris JP. Intratympanic gentamicin therapy for Meniere’s disease: a meta-analysis. Otol Neurotol. 2004;25(4):544–52.

131. Hellstrom S, Odkvist L. Pharmacologic labyrinthectomy. Otolaryngol Clin North Am. 1994;27(2):307–15.

132. Marzo SJ. Intratympanic therapies for sensorineural hearing loss and vertigo. ORL Head Neck Nurs. 2003;21(3):9–13.

133. Plontke SK, Götze G, Rahne T, Liebau A. Intracochlear drug delivery in combination with cochlear implants Current aspects. HNO. 2017;65:19–28.

134. Bowe SN, Jacob A. Round window perfusion dynamics: implications for intracochlear therapy. Curr Opin Otolaryngol Head Neck Surg. 2010;18(5):377–85.

135. Plontke S. Therapy of hearing disorders - conservative procedures. GMS Curr Top Otorhinolaryngol Head Neck Surg. 2005;4:Doc01.

136. Seidman. Glutamate Antagonists, Steroids, and Antioxidants as Therapeutic Options for Hearing Loss and Tinnitus and the Use of an Inner Ear Drug Delivery System. Int Tinnitus J. 1998;4(2):148–54.

137. Meyer T. Intratympanic treatment for tinnitus: a review. Noise Health. 2013;15(63):83–90.

138. Nakagawa T, Ito J. Local drug delivery to the inner ear using biodegradable materials. Ther Deliv. 2011;2(6):807–14.

139. Nakagawa T, Ito J. Drug delivery systems for the treatment of sensorineural hearing loss. Acta Otolaryngol Suppl. 2007;(557):30–5.

140. Okano T, Kelley MW. Stem cell therapy for the inner ear: recent advances and future directions. Trends Amplif. 2012;16(1):4–18.

141. Van de Water TR, Staecker H, Halterman MW, Federoff HJ. Gene therapy in the inner ear. Mechanisms and clinical implications. Ann N Y Acad Sci. 1999;884:345–60.

142. Musazzi UM, Franzé S, Cilurzo F, Franze S, Cilurzo F, Franzé S, et al. Innovative pharmaceutical approaches for the management of inner ear disorders. Drug Deliv Transl Res. 2017 May 1;1–14.

143. Valente F, Astolfi L, Simoni E, Danti S, Franceschini V, Chicca M, et al. Nanoparticle drug delivery systems for inner ear therapy: An overview. J Drug Deliv Sci Technol. 2017;39:28–35.

144. Agrahari V, Agrahari V, Mitra AK. Inner ear targeted drug delivery: what does the future hold?. Ther Deliv. 2017;8(4):179–84.

145. Berryhill WE, Graham MD. Chemical and physical labyrinthectomy for Meniere’s disease. Otolaryngol Clin North Am. 2002;35(3):675–82.

146. Garavello W, Galluzzi F, Gaini RM, Zanetti D. Intratympanic steroid treatment for sudden deafness: a meta-analysis of randomized controlled trials. Otol Neurotol. 2012;33(5):724–9.

147. Chien WW, Monzack EL, McDougald DS, Cunningham LL. Gene therapy for sensorineural hearing loss. Ear Hear. 2015;36(1):1–7.

148. Zhang W, Kim SM, Wang W, Cai C, Feng Y, Kong W, et al. Cochlear Gene Therapy for Sensorineural Hearing Loss: Current Status and Major Remaining Hurdles for Translational Success. Front Mol Neurosci. 2018;11:221.

149. Han X, Yin X, Du X, Sun C. Combined Intratympanic and Systemic Use of Steroids as a First-Line Treatment for Sudden Sensorineural Hearing Loss: A Meta-Analysis of Randomized, Controlled Trials. Otol Neurotol. 2017;38(4):487–95.

150. Gao Y, Liu D. Combined intratympanic and systemic use of steroids for idiopathic sudden sensorineural hearing loss: a meta-analysis. Eur Arch Oto-Rhino-Laryngology. 2016;273(11):3699–711.

151. Park Y. Stem Cell Therapy for Sensorineural Hearing Loss, Still Alive? J Audiol Otol. 2015;19(2):63–7.

152. Zenner H-P, Delb W, Kröner-Herwig B, Jäger B, Peroz I, Hesse G, et al. A multidisciplinary systematic review of the treatment for chronic idiopathic tinnitus. Eur Arch Oto-Rhino-Laryngology. 2017 May 19;274(5):2079–91.

153. Bear ZW, Mikulec AA. Intratympanic steroid therapy for treatment of idiopathic sudden sensorineural hearing loss. Mo Med. 2014;111(4):352–6.

154. Kitahara T. Evidence of surgical treatments for intractable Meniere’s disease. Auris Nasus Larynx. 2018;45(3):393–8.

155. Yetişer S. Intratympanic gentamicin for intractable Meniere’s disease - a review and analysis of audiovestibular impact. Int Arch Otorhinolaryngol. 2018;22(2):190–4.

156. Patel M. Intratympanic corticosteroids in Meniere’s disease: A mini-review. J Otol. 2017;12(3):117–24.

157. Brigande J. Hearing in the mouse of Usher. Nat Biotechnol. 2017;35(3):216–8.

158. Kingwell K. Hearing loss: Vector overcomes barrier to gene therapy delivery. Nat Rev Drug Discov. 2017;16(4):238–9.
